# Hypertensive disorders of pregnancy and childhood neurodevelopment: A systematic review and meta-analysis

**DOI:** 10.1371/journal.pmed.1004558

**Published:** 2025-09-10

**Authors:** Jessica A. Atkinson, Hannah G. Gordon, Stephen Tong, Susan P. Walker, Parinaz Mehdipour, Anthea C. Lindquist, Roxanne M. Hastie

**Affiliations:** 1 Perinatal Epidemiology Group, Department of Obstetrics, Gynaecology, and Newborn Health, University of Melbourne, Melbourne, Victoria, Australia; 2 Mercy Perinatal, Mercy Hospital for Women, Heidelberg, Victoria, Australia; 3 Faculty of Health, Deakin University, Burwood, Victoria, Australia; University of Manchester, UNITED KINGDOM OF GREAT BRITAIN AND NORTHERN IRELAND

## Abstract

**Background:**

Hypertensive disorders of pregnancy may be associated with an increased risk of adverse neurodevelopmental outcomes for the child, though no recent comprehensive meta-analyses exist. The aim of this study was to conduct a systematic review and meta-analysis examining the association between hypertensive disorders of pregnancy and child neurodevelopmental disabilities, intelligence, and educational outcomes.

**Methods and findings:**

A search was conducted of MEDLINE, CINAHL, Web of Science, and PsycINFO databases from inception until 18 September 2024. Reference lists of included papers were also screened. Observational studies and secondary analyses of randomized trials reporting neurodevelopmental, cognitive, or educational outcomes for children born following hypertensive disorders of pregnancy against a reference population (unaffected pregnancies) were included. Two reviewers independently screened records, extracted data, and assessed quality of studies using Preferred Reporting Items for Systematic Reviews and Meta-Analyses. Studies reporting similar outcomes were pooled using a random-effects meta-analysis model. Outcomes included autism, attention-deficit/hyperactivity disorder, cerebral palsy, global developmental delay, intellectual disability, intelligence quotient, and educational attainment. Results were reported as odds ratios (OR) or mean difference (MD) with corresponding 95% confidence intervals (CI). After screening 13,419 records, 121 studies reporting outcomes of 29,649,667 offspring were included. We included 85 cohort studies, 30 case-control studies, four cross-sectional studies, and two secondary analyses of randomized trials. Compared with unaffected pregnancies, hypertensive disorders of pregnancy were associated with an increased unadjusted likelihood of autism spectrum disorder (OR 1.65 (95% CI [1.49,1.83]); *p* < 0.001; *n* = 26,727,500), attention-deficit/hyperactivity disorder (OR 1.27 (95% CI [1.21,1.33]); *p* < 0.001; *n* = 12,987,737), intellectual disability (OR 1.77 (95% CI [1.31,2.38]); *p* < 0.001; *n* = 10,718,504), global developmental delay (OR 1.77 (95% CI [1.21,2.59]); *p* < 0.001; *n* = 2,961,195), and reduced mean intelligence (MD −2.20 95% CI [−3.35,-1.06]); *p* < 0.001; *n* = 1,150,664). Associations between hypertension and autism spectrum disorder and global developmental delay were no longer significant after adjusting for gestational age and birthweight. Results for intelligence quotient remained significant when adjusting for birthweight, but not gestational age. Adjusted analyses for attention-deficit/hyperactivity disorder and intellectual disability could not be performed due to a lack of suitable studies. In sensitivity analyses, results were unchanged after exclusion of papers at high risk of bias. This study is limited by a lack of constituent papers which adjusted for confounding and mediating factors, a high amount of heterogeneity among included studies, and possible publication bias for some outcomes.

**Conclusions:**

Hypertensive disorders of pregnancy are potentially associated with adverse neurodevelopmental and cognitive outcomes among affected offspring. While the mechanisms driving these associations are not clear, these results highlight a group of children that will benefit from early intervention and support to improve their neurodevelopmental outcomes.

## Author summary

**Why was this study done?:**

- Hypertensive disorders of pregnancy may be associated with adverse childhood neurodevelopmental outcomes, but no recent meta-analyses have examined the association comprehensively.

**What did the researchers do and find?:**

- We conducted a systematic review and meta-analysis, pooling data from 121 studies reporting neurodevelopmental outcomes for over 29 million children following hypertensive disorders of pregnancy.
- Hypertensive disorders of pregnancy were associated with an unadjusted increased likelihood of childhood autism spectrum disorder, attention-deficit/hyperactivity disorder, global developmental delay, intellectual disability, and reduced mean intelligence scores.
- There was no evidence of clinically meaningful associations between hypertensive disorders of pregnancy and any adverse neurodevelopmental outcomes when adjusting for gestational age at birth or birthweight.

**What do these findings mean?:**

- These findings demonstrate a potential association between hypertensive disorders of pregnancy and adverse childhood neurodevelopmental outcomes.
- We have identified a group of children who may benefit from early intervention and support to improve long-term neurodevelopmental outcomes.
- Further research is required to assess the mediating effects of gestational age and birthweight on these associations.

## Abstract 3

Jessica A Atkinson and colleagues conduct a systematic review and meta-analysis to examine the association between hypertensive disorders of pregnancy and child neurodevelopmental disabilities, intelligence, and educational outcomes.

## Introduction

Hypertensive disorders of pregnancy, including preeclampsia, gestational hypertension, and chronic hypertension, affect up to 10% of pregnancies globally [1]. Complications arising from these disorders include fetal growth restriction, preterm birth, and, in severe cases, maternal or neonatal death [2]. The long-term outcomes for affected women are clear—mothers have a lifelong increased risk of stroke, cardiovascular disease, metabolic syndromes, and premature death [3]. However, the outcomes for their children are not well understood.

Despite increasing prevalence in recent decades, the origins of neurodevelopmental disorders are not well-defined [4,5]. It has been proposed that the prenatal environment may play a large role in the development of neurodevelopmental disability [6]. During gestation, the fetal brain undergoes rapid growth which can be interrupted by factors such as inflammation and oxidative stress; both of which are excessive among women with hypertensive disorders of pregnancy [2,6].

Indeed, observational data suggests that hypertensive disorders of pregnancy may be associated with neurodevelopmental disorders, including autism spectrum disorder, attention-deficit/hyperactivity disorder, and cognitive delay [7–15]. However, there have been no recent systematic reviews examining the association with broader developmental or educational outcomes. Nor have there been any meta-analyses examining the effects of mediating factors on these associations. The aim of this study was therefore to conduct a comprehensive and contemporaneous systematic review and meta-analysis to examine the impact of hypertensive disorders of pregnancy on childhood and adolescent neurodevelopmental outcomes.

## Methods

Our study follows the Preferred Reporting Items for Systematic Reviews and Meta-Analyses (PRISMA) guidelines (Table A in S1 Appendix) [16]. This study was prospectively registered at the International Register of Systematic Reviews (PROSPERO; CRD42023442501).

### Search strategy

We conducted a search of MEDLINE, CINAHL, Web of Science Core Collections, and PsycINFO databases from inception until 18 September 2024. Databases were searched for the keywords, “preeclampsia”, “gestational hypertension”, “hypertension”, “pregnancy”, “obstetrics”, “neurodevelopment”, “education”, “intelligence”, “disability”, “offspring”, and “child”. The full search strategy is presented in Table B in S1 Appendix. Additional eligible studies were identified by manually screening the reference lists of included papers.

### Study selection criteria

We included secondary analyses of randomized trials and observational data from cohort, case-control, and cross-sectional studies. Papers were included if they reported outcomes of offspring born to mothers who experienced (1) preeclampsia; (2) gestational hypertension; or (3) chronic hypertension during pregnancy, compared with normotensive women.

Studies were eligible if they reported any of the following outcomes: (1) a diagnosis of neurodevelopmental disability; (2) intelligence quotient (IQ) measured using a validated tool; (3) global developmental delay and/or its components measured using a validated tool; or (4) educational achievement measured using standardized testing. Review papers, case reports or case series, conference papers, theses or dissertations, and abstracts only were excluded. Publications in languages other than English were translated and assessed for eligibility.

### Data extraction

Studies identified by the search strategy were uploaded into the review software, Covidence [17], and duplicates were removed. Two authors (JA and HG) independently screened titles and abstracts and completed full-text review.

Data extraction was independently performed by two reviewers (JA and HG) with discrepancies resolved by consultation with a third reviewer (RH). Extracted data included title, author(s), study design, year and country, inclusion and exclusion criteria, population characteristics, outcome/s of interest, and available demographics of children at birth and at assessment (Table C in S1 Appendix). REDCap data management software hosted at the University of Melbourne was used to facilitate data extraction [18,19].

### Risk of bias evaluation

Two authors (JA and HG) independently determined risk of bias for each included study. The Newcastle–Ottawa Scale (NOS) was used to determine risk of bias for observational studies and secondary analyses of randomized trials [20]. Observational studies were rated ‘good’ quality if they scored ≥7 stars of a possible 9; ‘fair’ quality if they scored ≥4 and ≤6 stars; and ‘poor’ quality if they scored ≤3 stars (Table E in S1 Appendix). Publication bias was visualized using funnel plots (Fig E in S1 Appendix).

### Data synthesis

For binary outcomes, we calculated pooled odds ratios (ORs) and 95% confidence intervals (CIs) using the restricted maximum likelihood (REML) approach to random-effects meta-analysis [21]. For continuous outcomes, we calculated mean difference (MD) and 95% CI using REML. For pooled estimates, the *I*^*2*^ statistic was used to quantify heterogeneity. *P-*valued were two-sided and significance was set at *p < 0.05*.

Unadjusted estimates were calculated as the primary analysis. Where possible, raw data were extracted from each paper and used for meta-analysis. Where raw data were unavailable, reported univariate estimates (OR or MD) were included in the meta-analysis. Adjusted estimates were extracted as secondary outcomes where appropriate data were available.

Outcomes of interest included (1) autism spectrum disorder; (2) attention-deficit/hyperactivity disorder; (3) cerebral palsy; (4) intellectual disability; (5) global developmental delay and its sub-components; (6) mean intelligence score; and (7) educational achievement.

For longitudinal studies reporting outcomes at different time points, we avoided double-counting of participants by extracting only the most recent assessment performed. Where papers reported multiple hypertensive disorders of pregnancy (e.g., preeclampsia and gestational hypertension) and could not be combined, these cohorts were analyzed separately.

### Subgroup and sensitivity analyses

We conducted subgroup analyses by (1) type of hypertensive disorder (preeclampsia, gestational hypertension, or chronic hypertension); and (2) age at assessment (infant [0–2 years]; child [3–5 years]; primary school age [6–11 years]; adolescent [12–17 years]; or adult [≥18 years]) (Table H in S1 Appendix). We performed sensitivity analyses by excluding studies deemed to be at high risk of bias (scoring poor/fair), as suggested by Higgins and colleagues [22].

There are several potentially important mediators of the association between hypertensive disorders of pregnancy and neurodevelopmental disability. Two factors we considered were gestational age and birthweight. Children born preterm (<37 weeks’ gestation) and even early-term (37–38 weeks’ gestation) are at increased risk of poor neurodevelopmental outcomes compared with their full-term (39–40 weeks’ gestation) born peers [23–26]. Importantly, hypertensive disorders of pregnancy often necessitate earlier birth [27,28]. Additionally, low birthweight is associated with adverse neurodevelopment, and children born following hypertensive disorders of pregnancy are at increased risk of being growth-restricted or of low birthweight [27–30].

We therefore conducted two prespecified sensitivity analyses among papers which reported results adjusted for (a) gestational age at birth, and (b) birthweight (Table 2). These analyses only included studies which had reported multivariable ORs or MDs adjusting for gestational age at birth or low birthweight.

**Table 1:** Unadjusted associations between hypertensive disorders of pregnancy and neurodevelopmental disability, stratified by type of hypertensive disorder.

Outcome	Papers (*n*)	References	Hypertensive (*n*)	Normotensive (*n*)	I^2^ (%)	Pooled Effect Size (95% CI)
**Autism spectrum disorder** ^a^						
Any hypertensive disorder	41	[33,35,36,43,46–48,50,51,55,58,59,62–64,67,70,75,82,84–87,89,90,92,97,100,103,110,113,115,116,122,123,132,138,140,141,144,147]	830,701	25,896,799	94.26	1.65 (1.49, 1.83)
Preeclampsia	25	[33,47,48,50,51,55,58,59,70,75,85,87,92,97,103,110,113,115,123,132,138,140,141,144,147]	567,392	15,889,329	96.20	1.73 (1.47, 2.04)
Gestational hypertension	9	[36,43,47,63,67,85,116,141,144]	42,950	4,540,432	39.37	1.44 (1.24, 1.67)
Chronic hypertension	9	[43,55,85,86,110,113,140,141,144]	90,808	11,285,088	88.66	1.46 (1.14, 1.88)
Unspecified hypertension	12	[35,46,62,64,82,84,86,89,90,97,100,122]	129,551	3,935,523	87.81	1.60 (1.37, 1.86)
**Attention-deficit/hyperactivity disorder (ADHD)** ^a^						
Any hypertensive disorder	19	[34,40,44,46,49,55,61,65,70,72,76,87,98,102,117,121,132,133,141]	504,402	12,483,335	82.23	1.27 (1.21, 1.33)
Preeclampsia	14	[34,40,49,55,65,70,72,76,87,98,102,132,133,141]	305,918	10,589,077	22.50	1.27 (1.24, 1.30)
Gestational hypertension	2	[121,141]	27,659	4,318,211	0.00	1.49 (1.39, 1.60)
Chronic hypertension	2	[55,141]	43,829	5,155,721	99.17	1.06 (0.57, 1.98)
Unspecified hypertension	5	[44,46,49,61,117]	126,996	1,881,130	91.89	1.28 (1.05, 1.55)
**Cerebral palsy** ^a^						
Any hypertensive disorder	18	[32,55,57,77,104,115,120,130,132,134,135,137,149,151]	91,310	2,485,997	95.56	1.28 (0.76, 2.13)
Preeclampsia	13	[55,57,77,104,111,112,114,115,120,130,132,134,151]	81,101	2,476,939	96.00	1.58 (0.80, 3.14)
Gestational hypertension	0	–	–	–	–	–
Chronic hypertension	3	[55,114,149]	7,982	851,081	81.12	1.12 (0.28, 4.50)
Unspecified hypertension	5	[32,108,135,137,149]	2,227	8,687	77.70	0.86 (0.47, 1.60)
**Intellectual disability** ^a^						
Any hypertensive disorder	12	[46,55,59,79,87,95,132,135,139,141,143,151]	395,813	10,322,691	98.49	1.77 (1.31, 2.38)
Preeclampsia	9	[55,59,79,87,132,139,141,143,151]	271,053	9,041,734	98.15	1.81 (1.33, 2.45)
Gestational hypertension	2	[141,143]	30,254	4,402,266	79.97	1.38 (0.92, 2.09)
Chronic hypertension	3	[55,141,143]	50,113	5,252,976	99.26	2.22 (0.59, 8.35)
Unspecified hypertension	3	[46,95,135]	44,393	1,280,957	92.14	1.92 (0.70, 5.30)
**Global developmental delay** ^a^						
Any hypertensive disorder	32	[37,39,52,54–56,60,62,66,78,81,87,88,90,91,93,99,101,105,108,126,128,130,131,134,135,140,142,145,151]	85,518	2,876,195	99.58	1.77 (1.21, 2.59)
Preeclampsia	18	[55,56,60,66,74,81,87,88,91,101,105,128,130,131,134,140,145,151]	56,278	2,098,393	96.04	1.25 (1.02, 1.53)
Gestational hypertension	7	[56,66,74,81,91,93,128]	15,006	912,337	85.23	1.32 (1.09, 1.61)
Chronic hypertension	7	[55,56,74,81,91,128,140]	10,258	1,031,930	66.32	1.42 (1.16, 1.73)
Unspecified hypertension	14	[37,39,52,54,62,78,80,91,99,108,126,135,142]	3,976	39,950	98.16	2.73 (1.25, 5.96)
**Intelligence quotient (IQ)** ^b^						
Any hypertensive disorder	12	[41,46,60,69,94,96,106,107,112,127,146,151]	45,221	1,105,423	99.97	−2.20 (−3.35, −1.06)
Preeclampsia	8	[60,69,94,96,106,112,127,146]	1,564	50,212	99.97	−3.19 (−4.66, −1.71)
Gestational hypertension	3	[69,94,146]	603	12,966	0.00	−1.55 (−2.64, −0.45)
Chronic hypertension	0	–	–	–	–	–
Unspecified hypertension	4	[41,46,107,150]	43,054	1,042,245	82.30	1.55 (−4.21, 7.31)

^a^Effect size reported as odds ratio (95% confidence interval).

^b^Effect size reported as mean difference (95% confidence interval).

**Table 2:** Association between hypertensive disorders of pregnancy and neurodevelopmental disability, limited to studies which adjusted for key mediating factors.

Outcome	No. studies	References	No. participants	*I*^2^ (%)	Unadjusted effect estimate (95% CI)	Adjusted effect estimate (95% CI)
**Adjusted for gestational age at birth**						
Autism spectrum disorder^a^	3	[35,84,85]	22,100	0.00	1.21 (1.02, 1.43)	1.17 (0.98, 1.39)
Attention-deficit/hyperactivity disorder^a^	1	[65]	12,622	–	3.32 (1.72, 6.41)^*^	2.77 (1.42, 5.39)^*^
Cerebral palsy^a^	3	[111,114,120]	459,440	89.44	1.87 (0.52, 6.68)	1.32 (0.48, 3.67)
Intellectual disability^a^	1	[79]	80,876	–	1.55 (1.31, 1.83)^*^	1.58 (1.34, 1.87)^*^
Global developmental delay^a^	4	[74,81,126,131]	30,674	73.01	1.27 (0.90, 1.79)	1.16 (0.85, 1.60)
Intelligence quotient (IQ)^b^	3	[46,136,146]	1,088,785	0.00	−1.58 (−3.29, 0.14)	−1.81 (−3.80, 0.19)
**Adjusted for birthweight**						
Autism spectrum disorder^a^	1	[84]	2,488	–	1.83 (0.58, 5.76)^*^	1.60 (0.89, 2.87)^*^
Attention-deficit/hyperactivity disorder^a^	1	[102]	84,721	–	1.14 (1.04, 1.26)^*^	1.19 (1.03, 1.37)^*^
Cerebral palsy^a^	2	[111,120]	458,814	92.21	3.31 (0.84, 17.26)	2.17 (0.56, 8.36)
Intellectual disability^a^	0	–	–	–	–	–
Global developmental delay^a^	3	[74,81,131]	29,120	73.29	1.22 (0.79, 1.88)	1.16 (0.79, 1.69)
Intelligence quotient (IQ)^b^	4	[46,136,146]	1,122,808	99.35	−1.72 (−2.90, −0.54)	−2.01 (−3.37, −0.64)

^a^Effect size reported as odds ratio (95% confidence interval).

^b^Effect size reported as mean difference (95% confidence interval).

* Estimate obtained from a single paper; not meta-analyzed.

All analyses were conducted in Stata-SE, version 18 (StataCorp LLC) [31].

## Results

### Flow and characteristics of included studies

**Fig 1:**
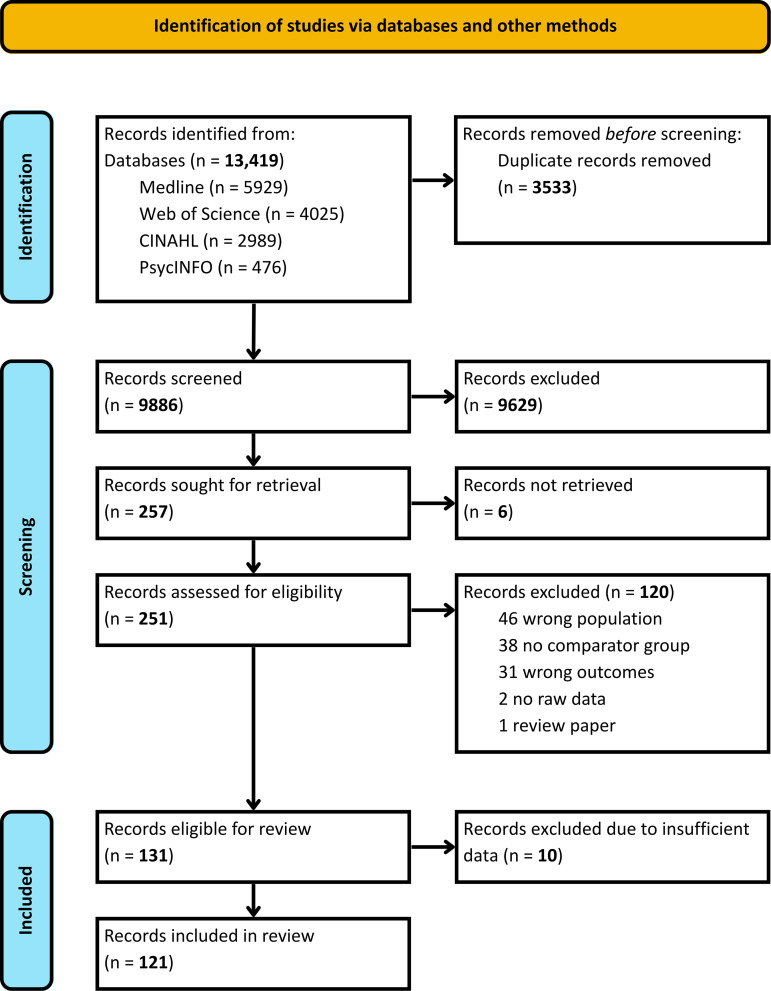
PRISMA flow diagram.

**Fig 2:**
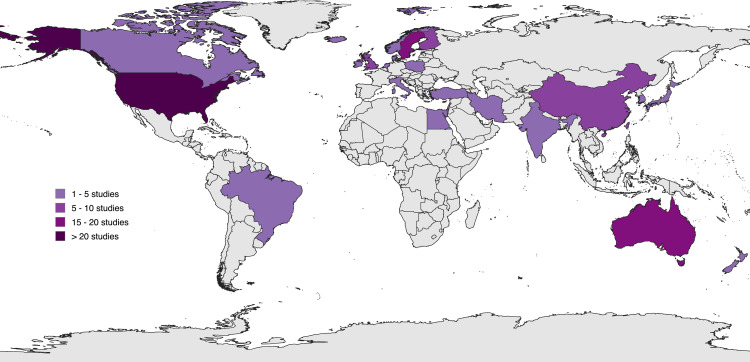
Distribution of included studies. Created with PlaniGlobe, http://www.planiglobe.com, CC BY 2.0.

Our search identified 13,419 studies; of these, 251 full texts were reviewed and 131 were deemed eligible for inclusion (Fig 1) [32–152]. A further 10 studies were excluded due to insufficient available data (authors were contacted to request data; Table D in S1 Appendix) [153–162]. Cumulatively, the 121 included papers reported outcomes of 29,649,667 offspring, including 1,117,193 born following hypertensive disorders of pregnancy (4.0% prevalence). Studies originated from 31 different countries, including 19 studies from eight low- and middle-income countries (Fig 2).

### Autism spectrum disorder

**Fig 3:**
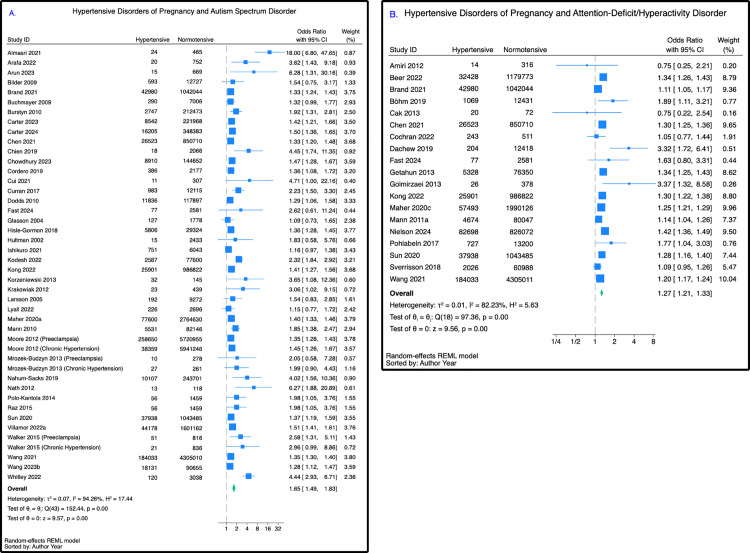
Association between hypertensive disorders of pregnancy and autism spectrum disorder and attention-deficit/hyperactivity disorder. **(A)** Autism spectrum disorder, reported as an unadjusted odds ratio (95% CI). **(B)** Attention-deficit/hyperactivity disorder, reported as an unadjusted odds ratio (95% CI).

Autism spectrum disorder was the most reported neurodevelopmental disorder (41 papers). Pooling these studies, offspring born following a pregnancy affected by hypertension had a 65% increased likelihood of developing autism spectrum disorder, compared with their unexposed peers (unadjusted OR 1.65 (95% CI [1.49,1.83]); *p* < 0.001; *n* = 26,727,500) (Fig 3).

We next investigated this association by type of hypertensive disorder. Children born following a preeclamptic pregnancy had a 73% increased likelihood of developing autism spectrum disorder (OR 1.73 (95% CI [1.47,2.04]); *p* < 0.001; *n* = 16,456,721) compared with those born following a normotensive pregnancy. Both gestational hypertension (OR 1.44 (95% CI [1.24,1.67]); *p* < 0.001; *n* = 4,583,382) and chronic hypertension (OR 1.46 (95% CI [1.14,1.88]); *p* < 0.001; *n* = 11,375,896) were associated with significant but attenuated odds of childhood autism spectrum disorder compared with normotensive pregnancies (Table 1). However, the differences between groups were not deemed to be statistically significant (*p = 0.38)* (Fig C in S1 Appendix).

### Attention-deficit/hyperactivity disorder

Nineteen papers assessed childhood attention-deficit/hyperactivity disorder following hypertensive disorders of pregnancy. Among these, hypertension was associated with a 27% increased likelihood of childhood attention-deficit/hyperactivity disorder, compared with normotensive pregnancies (OR 1.27 (95% CI [1.21,1.33]); *p* < 0.001; *n* = 12,987,737) (Fig 3).

Of the hypertensive subtypes, gestational hypertension was most strongly associated with childhood attention-deficit/hyperactivity disorder (OR 1.49 (95% CI [1.39,1.60]); *p* < 0.001; *n* = 4,345,870), however, only two papers could be included in this meta-analysis. Preeclampsia was also associated with an increased likelihood of attention-deficit/hyperactivity disorder (OR 1.27 (95% CI [1.24,1.31]); *p* < 0.001; *n* = 10,894,995), but there was no association with chronic hypertension (OR 1.06 (95% CI [0.57,1.98]); *p* = 0.85; *n* = 5,199,550) (Table 1). The differences between subgroups were statistically significant (*p* < 0.001) (Fig C in S1 Appendix).

### Cerebral palsy

**Fig 4:**
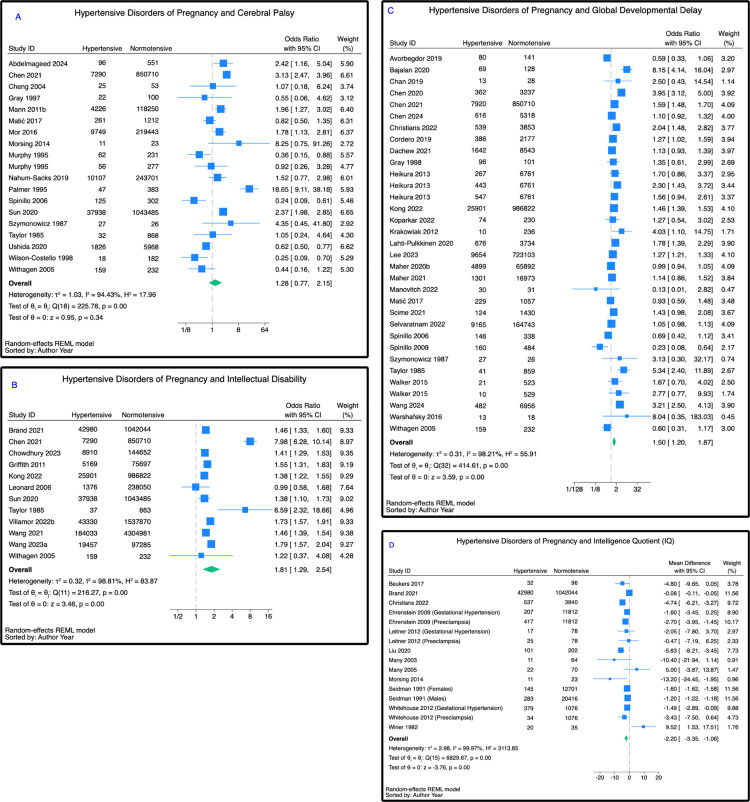
Association between hypertensive disorders of pregnancy and other neurodevelopmental disabilities. **(A)** Global developmental delay, reported as an unadjusted odds ratio (95% CI). **(B)** Cerebral palsy, reported as an unadjusted odds ratio (95% CI). **(C)** Intellectual disability, reported as an unadjusted odds ratio (95% CI). **(D)** Intelligence quotient (IQ), reported as an unadjusted mean difference (95% CI).

Pooling the results of 18 papers, hypertensive disorders of pregnancy were not associated with an increased likelihood of childhood cerebral palsy (OR 1.28 (95% CI [0.76,2.13]); *p* = 0.35; *n* = 2,557,307), compared with normotensive pregnancies (Fig 4). This remained true among preeclamptic (OR 1.58 (95% CI [0.80,3.14]); *p* = 0.19; *n* = 2,558,040) and chronic hypertensive (OR 1.12 (95% CI [0.28,4.50]); *p* = 0.88; *n* = 859,063) pregnancies, and there was no statistically significant difference between the groups *(*p** = 0.44) (Fig D in S1 Appendix). No studies examined the association between gestational hypertension and cerebral palsy.

### Intellectual disability

Twelve papers examined the association between hypertensive disorders of pregnancy and intellectual disability. Pooling these, hypertensive disorders of pregnancy were associated with a 77% increased likelihood of intellectual disability (OR 1.77 (95% CI [1.31,2.38]); *p* < 0.001; *n* = 10,718,504), compared with normotensive pregnancies (Fig 4). There was no statistically significant difference between the types of hypertension and odds of intellectual disability (*p* = 0.73) (Fig D in S1 Appendix).

### Global developmental delay

The association between hypertensive disorders of pregnancy and global developmental delay was reported by 32 papers. Among these, hypertensive disorders of pregnancy were associated with a 77% increased likelihood of global developmental delay (OR 1.77 (95% CI [1.21,2.59]); *p* < 0.001; *n* = 2,961,195) (Fig 4). There was no statistically significant difference between the different types of hypertension and odds of global developmental delay (*p* = 0.27) (Fig D in S1 Appendix).

We also investigated nine components of developmental delay (Table F in S1 Appendix). These were behavior, cognition, communication, fine motor, gross motor, hyperactivity/impulsivity, language, personal-social skills, and problem solving. Pooling studies of comparable outcomes, we found an association between hypertensive disorders of pregnancy and fine motor delay (OR 1.17 (95% CI [1.04,1.32]); *p* < 0.001; *n* = 934,534), gross motor delay (OR 1.23 (95% CI [1.08,1.40]); *p* < 0.001; *n* = 934,536), and personal-social problems (OR 1.21 (95% CI [1.05,1.41]); *p* < 0.001; *n* = 281,709).

### Intelligence quotient (IQ)

Thirteen papers reported the association between hypertensive disorders of pregnancy and mean IQ. Hypertensive disorders of pregnancy were associated with a mean reduction of 2.20 IQ points among affected offspring (MD −2.20 (95% CI [−3.35,-1.06]); *p* < 0.001; *n* = 1,150,664), compared with normotensive pregnancies (Fig 4). There was no statistically significant difference between the different types of hypertension and mean intelligence quotient (*p* = 0.10) (Fig D in S1 Appendix).

### Educational achievement

Pooling studies which examined childhood educational achievement, we found that hypertensive disorders of pregnancy were associated with an increased likelihood of overall poor educational achievement (OR 1.36 (95% CI [1.27,1.45]); *p* < 0.001; *n* = 475,452) and poor reading achievement (OR 1.14 (95% CI [1.05,1.23]); *p* < 0.001; *n* = 64,943), although although only two papers were included in each of these meta-analyses. There was no association between hypertensive disorders of pregnancy and mathematics achievement (OR 1.08 (95% CI [0.85,1.36]); *p* = 0.53; *n* = 64,943) (Table G in S1 Appendix).

### Adjusted analyses

Few studies reported results adjusted for gestational age at birth (13 papers) or birthweight (nine papers). To assess the effect of these key mediating factors, we calculated both the unadjusted and adjusted pooled effects for this limited subgroup of papers (Table 2).

Among three papers which adjusted for gestational age at birth, the association between hypertensive disorders of pregnancy and autism spectrum disorder was no longer significant (OR 1.21 (95% CI [1.02,1.45]); adjusted odds ratio (aOR) 1.17 (95% CI [0.98,1.39]); *p* = 0.08; *n* = 22,100). One study adjusted for birthweight and showed no significant association in either their adjusted or unadjusted analyses (OR 1.83 (95% CI [0.58,5.76]); aOR 1.60 (95% CI [0.90,2.90]); *n* = 2,448) (Fig A in S1 Appendix) [84].

Only one paper reported the odds of attention-deficit/hyperactivity disorder adjusted for gestational age at birth [65]. Among 12,622 children, this association remained significant (OR 3.32 (95% CI [1.72,6.41]); aOR 2.77 (95% CI [1.42,5.39])). Similarly, one paper reported the odds of attention-deficit/hyperactivity disorder adjusted for birthweight and showed a significant association (OR 1.14 (95% CI [1.04,1.26]); aOR 1.19 (95% CI [1.03,1.37]); *n* = 84,721) (Fig A in S1 Appendix) [102].

Hypertensive disorders of pregnancy were not associated with cerebral palsy when adjusting for gestational age at birth (aOR 1.32 (95% CI [0.48,3.67]); *p* = 0.59; *n* = 459,440) or birthweight (aOR 2.17 (95% CI [0.56,8.36]); *p* = 0.26; *n* = 458,814). There was no significant association between hypertensive disorders of pregnancy and global developmental delay among adjusted studies, for either the unadjusted or adjusted pooled OR (Fig B in S1 Appendix).

One paper reported an increased likelihood of intellectual disability following hypertensive disorders of pregnancy, even after adjusting for gestational age at birth (OR 1.55 (95% CI [1.31,1.83]); aOR 1.58 (95% CI [1.31,1.83]); *n* = 80,876) [79]. No papers reporting intellectual disability adjusted for birthweight. Hypertensive disorders of pregnancy were not associated with a reduction in IQ score when including only studies which adjusted for gestational age (MD −1.58 (95% CI [−3.29, 0.14]; aMD −1.81 (95% CI [−3.80, 0.19]). However, hypertensive disorders of pregnancy remained associated with a reduction in IQ score when adjusting for birthweight (MD −1.72 (95% CI [−2.90, −0.54]); aMD −2.01 (95% CI [−3.37, −0.64]), although this association is unlikely to be clinically meaningful (Fig B in S1 Appendix).

### Risk of bias evaluation

Using the NOS, 86 studies were found to be of good methodological quality; 30 were found to be of fair quality; and three were found to be of poor quality (Table E in S1 Appendix). There was significant variation in study design, sampled populations, reported outcomes and method of assessment, and in adjustment for confounding factors.

To investigate the variation in quality further, we performed a sensitivity analysis excluding all studies assessed as poor or fair quality. Excluding these 33 studies, the results did not differ substantially from our main analysis. In this analysis, hypertensive disorders of pregnancy were associated with an increased likelihood of autism (OR 1.42 (95% CI [1.36,1.49]); 31 studies; *n* = 26,180,175), attention-deficit/hyperactivity disorder (OR 1.28 (95% CI [1.21,1.35]); *p* < 0.001; 15 studies; *n* = 12,872,592), intellectual disability (OR 1.87 (95% CI [1.25,2.79]); 8 studies; *n* = 10,304,992), and global developmental delay (OR 1.41 (95% CI [1.15,1.73]); 23 studies; *n* = 2,925,771), as well as a decreased mean intelligence score (MD −2.20 (95% CI [−3.33,-1.07]); 14 studies; *n* = 1,150,518) (Table I in S1 Appendix).

Further, we assessed publication bias via funnel plots for our primary outcomes (Fig E in S1 Appendix). Plots for studies reporting attention-deficit/hyperactivity disorder, cerebral palsy, and IQ were generally symmetrical, indicating low risk of publication bias. However, plots for studies reporting autism spectrum disorder, global developmental delay, and intellectual disability were asymmetrical (clustered to the left), indicating potential publication bias and over-estimation of the true effect (Fig E in S1 Appendix).

## Discussion

In this systematic review of 121 studies and over 29 million children, hypertensive disorders of pregnancy were associated with an unadjusted increased likelihood of autism spectrum disorder, attention-deficit/hyperactivity disorder, intellectual disability, global developmental delay, and reduced mean intelligence scores. These findings suggest that hypertensive disorders of pregnancy may have a harmful effect on fetal neurodevelopment that has been previously overlooked.

To our knowledge, this is the largest and most comprehensive meta-analysis examining offspring neurodevelopment following hypertensive disorders of pregnancy. Our findings are consistent with previous reviews suggesting increased risks of autism spectrum disorder and attention-deficit/hyperactivity disorder [7–15]. We saw no increased likelihood of cerebral palsy, consistent with one previous review [163].

Whilst these findings are important, they must be interpreted with caution given the potential role of mediators in the relationship between hypertension and childhood development. We identified gestational age at birth and birthweight as two key mediators [23–30,164–166]. It is plausible that the observed association between hypertension and poor neurodevelopmental outcomes may in fact be driven by the increased rates of preterm birth and low birthweight in this cohort. To examine this further, we conducted adjusted analyses including only papers which adjusted for (a) gestational age and (b) birthweight. Among these studies, we saw attenuations in the association between hypertension and autism spectrum disorder and global developmental delay.

It is, however, important to note the significantly reduced sample sizes for our adjusted analyses (22,100–1.1 million offspring). It is therefore likely we were underpowered to detect true differences. We were also unable to perform adjusted analyses for other important outcomes or adjust for maternal confounding factors, such as age or body mass index, due to a lack of appropriate studies. It thus remains unclear to what extent the relationship between pregnancy hypertension and neurodevelopmental disability can be explained by external factors. Future studies should consider the use of individual participant data meta-analysis to allow further investigation. There was a paucity of relevant literature which accounted for key mediating and confounding factors, thus highlighting an avenue for future research. Until more robust, multivariable studies become available, the possibility of a direct pathophysiological impact of hypertension on fetal brain development cannot be excluded [167,168].

There was significant heterogeneity among the included studies (*I*^*2*^ ranging from 85.31% for attention-deficit/hyperactivity disorder to 99.97% for IQ). Studies varied in their sampled populations, methods, and assessed neurodevelopmental measures, adding challenge to the comparison of results of included papers. We attempted to explore this heterogeneity through our pre-specified subgroup analyses. We saw modest reductions in heterogeneity when stratifying by type of hypertensive disorder and timing of assessment, though heterogeneity remained high. There was little reduction in heterogeneity when excluding studies at high risk of bias. We anticipate that most of this heterogeneity has arisen from variation in methods and reporting of outcomes between studies, which we were unable to further correct for.

Studies reporting autism spectrum disorder, intellectual disability, and global developmental delay were also at high risk of publication bias. This is likely due to poor methodological design and lack of consistency in measuring tools across studies, which was also highlighted in our risk of bias assessment. Further, less publication bias was seen among studies reporting attention-deficit/hyperactivity disorder, cerebral palsy, and IQ, which largely used validated tools and clinical assessment of outcomes. Future studies will need to use more robust and consistent measures of childhood neurodevelopmental outcomes to limit bias and improve inter-study comparability.

In this pooled meta-analysis of 121 studies and over 29 million offspring, hypertensive disorders of pregnancy were associated with an increased likelihood of autism, attention-deficit/hyperactivity disorder, intellectual disability, global developmental delay, and with reduced mean intelligence and educational achievement. Although the biological basis of these associations remains unclear, our findings highlight a group of children that may benefit from access to early intervention and support to improve long-term neurodevelopmental outcomes.

## Supporting information

S1 Appendix Supplemental Tables and Figures. **Table A.** PRISMA checklist. **Table B.** Search strategy. **Table C.** Characteristics of included studies. **Table D.** Characteristics of excluded studies. **Table E.** Risk of bias assessment. **Table F.** Association between hypertensive disorders of pregnancy and individual components of global developmental delay. **Table G.** Association between hypertensive disorders of pregnancy and educational achievement. **Table H.** Association between hypertensive disorders of pregnancy and neurodevelopmental disability, stratified by age at assessment. **Table I.** Association between hypertensive disorders of pregnancy and neurodevelopmental disability, restricted to papers at low risk of bias. **Fig A.** Association between hypertensive disorders of pregnancy and neurodevelopmental disability, restricted to papers which adjusted for gestational age at birth. **Fig B.** Association between hypertensive disorders of pregnancy and neurodevelopmental disability, restricted to papers which adjusted for birthweight. **Fig C.** Association between hypertensive disorders of pregnancy and autism spectrum disorder and attention-deficit/hyperactivity disorder, stratified by type of hypertension. **Fig D.** Association between hypertensive disorders of pregnancy and other neurodevelopmental disabilities, stratified by type of hypertension. **Fig E.** Funnel plots detailing publication bias in meta-analyses for neurodevelopmental disabilities.
(DOCX)

S2 Appendix Dataset used for unadjusted analyses. (XLSX)

S3 Appendix Dataset used for adjusted analyses. (XLSX)

S4 Appendix Author-generated code for analyses. (DO)

S1 Text References included in S2 and S3 Appendices.
(DOCX)

## Abbreviations

aOR: adjusted odds ratio
CI: confidence interval
MD: mean difference
NOS: Newcastle–Ottawa Scale
OR: odds ratio
REML: restricted maximum likelihood
