# Supplementary material for: Hypertensive disorders of pregnancy and childhood neurodevelopment: A systematic review and meta-analysis
Source: PLoS Med. 2025 Sep 10;22(9):e1004558. doi: 10.1371/journal.pmed.1004558 (PMC12422451; doi:10.1371/journal.pmed.1004558)
Supplement: S1 Appendix — Table A. PRISMA checklist. Table B. Search strategy. Table C. Characteristics of included studies. Table D. Characteristics of excluded studies. Table E. Risk of bias assessment. Table F. Association between hypertensive disorders of pregnancy and individual components of global developmental delay. Table G. Association between hypertensive disorders of pregnancy and educational achievement. Table H. Association between hypertensive disorders of pregnancy and neurodevelopmental disability, stratified by age at assessment. Table I. Association between hypertensive disorders of pregnancy and neurodevelopmental disability, restricted to papers at low risk of bias. Fig A. Association between hypertensive disorders of pregnancy and neurodevelopmental disability, restricted to papers which adjusted for gestational age at birth. Fig B. Association between hypertensive disorders of pregnancy and neurodevelopmental disability, restricted to papers which adjusted for birthweight. Fig C. Association between hypertensive disorders of pregnancy and autism spectrum disorder and attention-deficit/hyperactivity disorder, stratified by type of hypertension. Fig D. Association between hypertensive disorders of pregnancy and other neurodevelopmental disabilities, stratified by type of hypertension. Fig E. Funnel plots detailing publication bias in meta-analyses for neurodevelopmental disabilities. (DOCX) [file pmed.1004558.s001.docx]

**Hypertensive disorders of pregnancy and childhood neurodevelopment: A systematic review and meta-analysis.**

***S1 Appendix.*** *Supplemental Tables & Figures*

**Supplemental Tables**

**Table A.** PRISMA checklist

**Table B.** Search strategy

**Table C.** Characteristics of included studies

**Table D.** Characteristics of excluded studies

**Table E.** Risk of bias assessment

**Table F.** Additional outcome data – components of global developmental delay

**Table G.** Additional outcome date – educational achievement

**Table H.** Subgroup analysis – age of assessment

**Table I.** Sensitivity analysis – risk of bias

**Supplemental Figures**

**Figure A.** Association between hypertensive disorders of pregnancy and neurodevelopmental disability, restricted to papers which adjusted for gestational age at birth

**Figure B.** Association between hypertensive disorders of pregnancy and neurodevelopmental disability, restricted to papers which adjusted for birthweight

**Figure C.** Association between hypertensive disorders of pregnancy and autism spectrum disorder and attention-deficit/hyperactivity disorder, stratified by type of hypertension

**Figure D.** Association between hypertensive disorders of pregnancy and other neurodevelopmental disabilities, stratified by type of hypertension

**Figure E.** Funnel plots detailing publication bias in meta-analyses for neurodevelopmental disabilities

**Table A. PRISMA Checklist**

| **Section and Topic** | **Item #** | **Checklist item** | **Location where item is reported** |
| --- | --- | --- | --- |
| **TITLE** | | |  |
| Title | 1 | Identify the report as a systematic review. | Cover Page, Paragraph 1 (P1) |
| **ABSTRACT** | | |  |
| Abstract | 2 | See the PRISMA 2020 for Abstracts checklist. | Abstract, P1-3 |
| **INTRODUCTION** | | |  |
| Rationale | 3 | Describe the rationale for the review in the context of existing knowledge. | Introduction, P1-3 |
| Objectives | 4 | Provide an explicit statement of the objective(s) or question(s) the review addresses. | Introduction, P3 |
| **METHODS** | | |  |
| Eligibility criteria | 5 | Specify the inclusion and exclusion criteria for the review and how studies were grouped for the syntheses. | Methods, P3 |
| Information sources | 6 | Specify all databases, registers, websites, organisations, reference lists and other sources searched or consulted to identify studies. Specify the date when each source was last searched or consulted. | Methods, P2, P8-11 |
| Search strategy | 7 | Present the full search strategies for all databases, registers and websites, including any filters and limits used. | S1 Appendix  Table 2 |
| Selection process | 8 | Specify the methods used to decide whether a study met the inclusion criteria of the review, including how many reviewers screened each record and each report retrieved, whether they worked independently, and if applicable, details of automation tools used in the process. | Methods, P4-5 |
| Data collection process | 9 | Specify the methods used to collect data from reports, including how many reviewers collected data from each report, whether they worked independently, any processes for obtaining or confirming data from study investigators, and if applicable, details of automation tools used in the process. | Methods, P6-7 |
| Data items | 10a | List and define all outcomes for which data were sought. Specify whether all results that were compatible with each outcome domain in each study were sought (e.g. for all measures, time points, analyses), and if not, the methods used to decide which results to collect. | Methods, P10 |
|  | 10b | List and define all other variables for which data were sought (e.g. participant and intervention characteristics, funding sources). Describe any assumptions made about any missing or unclear information. | Methods, P6 |
| Study risk of bias assessment | 11 | Specify the methods used to assess risk of bias in the included studies, including details of the tool(s) used, how many reviewers assessed each study and whether they worked independently, and if applicable, details of automation tools used in the process. | Methods, P7 |
| Effect measures | 12 | Specify for each outcome the effect measure(s) (e.g. risk ratio, mean difference) used in the synthesis or presentation of results. | Methods, P8 |
| Synthesis methods | 13a | Describe the processes used to decide which studies were eligible for each synthesis (e.g. tabulating the study intervention characteristics and comparing against the planned groups for each synthesis (item #5)). | Methods, P8-11 |
|  | 13b | Describe any methods required to prepare the data for presentation or synthesis, such as handling of missing summary statistics, or data conversions. | Methods, P8-11 |
|  | 13c | Describe any methods used to tabulate or visually display results of individual studies and syntheses. | Methods, P8-11 |
|  | 13d | Describe any methods used to synthesize results and provide a rationale for the choice(s). If meta-analysis was performed, describe the model(s), method(s) to identify the presence and extent of statistical heterogeneity, and software package(s) used. | Methods, P8-11 |
|  | 13e | Describe any methods used to explore possible causes of heterogeneity among study results (e.g. subgroup analysis, meta-regression). | Methods, P12-13 |
|  | 13f | Describe any sensitivity analyses conducted to assess robustness of the synthesized results. | Methods, P12-13 |
| Reporting bias assessment | 14 | Describe any methods used to assess risk of bias due to missing results in a synthesis (arising from reporting biases). | Methods, P13 |
| Certainty assessment | 15 | Describe any methods used to assess certainty (or confidence) in the body of evidence for an outcome. | Methods, P8 |
| **RESULTS** | | |  |
| Study selection | 16a | Describe the results of the search and selection process, from the number of records identified in the search to the number of studies included in the review, ideally using a flow diagram. | Results, P1  Figure 1 |
|  | 16b | Cite studies that might appear to meet the inclusion criteria, but which were excluded, and explain why they were excluded. | Results, P1  S1 Appendix, Table 4 |
| Study characteristics | 17 | Cite each included study and present its characteristics. | Results, P1  S1 Appendix, Table 3 |
| Risk of bias in studies | 18 | Present assessments of risk of bias for each included study. | S1 Appendix, Table 5 |
| Results of individual studies | 19 | For all outcomes, present, for each study: (a) summary statistics for each group (where appropriate) and (b) an effect estimate and its precision (e.g. confidence/credible interval), ideally using structured tables or plots. | Table 3-4  S1 Appendix, Table 6-9  S1 Appendix, Figure 1-4 |
| Results of syntheses | 20a | For each synthesis, briefly summarise the characteristics and risk of bias among contributing studies. | Table 3-4 |
|  | 20b | Present results of all statistical syntheses conducted. If meta-analysis was done, present for each the summary estimate and its precision (e.g. confidence/credible interval) and measures of statistical heterogeneity. If comparing groups, describe the direction of the effect. | Results, P1-20 |
|  | 20c | Present results of all investigations of possible causes of heterogeneity among study results. | Results, P13-20 |
|  | 20d | Present results of all sensitivity analyses conducted to assess the robustness of the synthesized results. | Results, P13-20 |
| Reporting biases | 21 | Present assessments of risk of bias due to missing results (arising from reporting biases) for each synthesis assessed. | Results, P13-20 |
| Certainty of evidence | 22 | Present assessments of certainty (or confidence) in the body of evidence for each outcome assessed. | Results, P1-20 |
| **DISCUSSION** | | |  |
| Discussion | 23a | Provide a general interpretation of the results in the context of other evidence. | Discussion, P1-3 |
|  | 23b | Discuss any limitations of the evidence included in the review. | Discussion, P4-7 |
|  | 23c | Discuss any limitations of the review processes used. | Discussion, P4-7 |
|  | 23d | Discuss implications of the results for practice, policy, and future research. | Discussion, P6-7  Conclusion, P1 |
| **OTHER INFORMATION** | | |  |
| Registration and protocol | 24a | Provide registration information for the review, including register name and registration number, or state that the review was not registered. | Methods, P1 |
|  | 24b | Indicate where the review protocol can be accessed, or state that a protocol was not prepared. | Methods, P1 |
|  | 24c | Describe and explain any amendments to information provided at registration or in the protocol. | N/A |
| Support | 25 | Describe sources of financial or non-financial support for the review, and the role of the funders or sponsors in the review. | Cover Page, P9 |
| Competing interests | 26 | Declare any competing interests of review authors. | Cover Page, P10 |
| Availability of data, code and other materials | 27 | Report which of the following are publicly available and where they can be found: template data collection forms; data extracted from included studies; data used for all analyses; analytic code; any other materials used in the review. | Cover Page, P8 |

**Table B. Search strategy**

| **Order** | **Search Terms** |
| --- | --- |
| 1 | exp Pre-Eclampsia/ or exp Hypertension, Pregnancy Induced/ or exp Eclampsia/ or exp HELLP Syndrome/  or  ((pregnancy AND hypertension) or (gestational hypertension) or pre-eclamp* or preeclamp* or eclamp* or HELLP syndrome).mp |
| 2 | exp Child/ or exp Child Development/ or exp Adolescent/  or  (child* or toddler* or offspring or adolescen* or pre-school or kinder* or school* or teenage* or young).mp |
| 3 | exp Neurodevelopmental Disorders/ or Intelligence/ or exp Cognition/ or exp Educational Measurement/ or exp Psychological Tests/  or  (performance or function* or develop* or cognit* or IQ or intelligence or neurodev* or neurocognit* or neurologic* or behavior* or behaviour* or academic or psychomotor or school or Wechsler scale* or disability).mp |
| 4 | 1 and 2 and 3 |
| 5 | Limit 4 to (case reports or comment or editorial or guideline or letter or retracted publication or "review") |
| 6 | 4 not 5 |
| 7 | Limit 6 to Animals/ |
| 8 | 6 not 7 |

**Table C. Characteristics of Included Studies**

| **Study ID** | **Country** | **Title** | **Study Design** | **Study Size** | **Outcome(s) Assessed** | **Method of Assessment** | **Age at Assessment** | **Maternal Diagnoses** |
| --- | --- | --- | --- | --- | --- | --- | --- | --- |
| Abdelmageed 2024 [1] | Canada | Association Between Maternal Hypertension and Infant Neurodevelopment in Extremely Preterm Infants | Cohort Study (Retrospective) | 647 | Cerebral Palsy; Global Development | Bayley Scales of Infant & Toddler Development | Infant (Age 0-2) | All Hypertensive Disorders/Not Stated |
| Almasri 2021 [2] | United States | Peripartum Antibiotics Exposure and the Risk of Autoimmune and Autism Disorders in the Offspring | Case-Control Study | 489 | Autism | ICD Coding | Primary School (Age 6-11) | Preeclampsia |
| Amiri 2012 [3] | Iran | Pregnancy-Related Maternal Risk Factors of Attention-Deficit Hyperactivity Disorder: A Case-Control Study | Case-Control Study | 330 | AD{HD | Clinical Diagnosis | Primary School (Age 6-11) | Preeclampsia |
| Arafa 2022 [4] | Egypt | Maternal and Neonatal Risk Factors for Autism Spectrum Disorder: A Case-Control Study from Egypt | Case-Control Study | 772 | Autism | Clinical Diagnosis | Primary School (Age 6-11) | All Hypertensive Disorders/Not Stated |
| Arun 2023 [5] | India | A Community-Based Study of Antenatal and Neonatal Risk Factors in Autism Spectrum Disorder[6] | Cross-Sectional Study | 687 | Autism | Clinical Diagnosis | Primary School (Age 6-11) | Gestational Hypertension |
| Avorbegdor 2019 [7] | United States | Health, Physical Growth, and Neurodevelopmental Outcomes in Preterm Infants of Women with Hypertensive Disorders of Pregnancy | Secondary Analysis of a Clinical Trial | 221 | Developmental Delay | Bayley Scales of Infant & Toddler Development | Infant (Age 0-2) | All Hypertensive Disorders/Not Stated |
| Ayala 2021 [8] | United States | Association Between Maternal Hypertensive Disorders, Fetal Growth and Childhood Learning Outcomes | Cohort Study (Retrospective) | 23094 | Education [9] | Standardised Testing | Primary School (Age 6-11) | All Hypertensive Disorders/Not Stated |
| Bajalan 2020 [10] | Iran | Risk Factors of Developmental Delay Among Infants Aged 6-18 Months | Case-Control Study | 197 | Developmental Delay | Ages & Stages Questionnaire | Infant (Age 0-2) | All Hypertensive Disorders/Not Stated |
| Beer 2022 [11] | Sweden | Associations of Preterm Birth, Small-for-Gestational Age, Preeclampsia and Placental Abruption with Attention-Deficit/ Hyperactivity Disorder in the Offspring: Nationwide Cohort and Sibling-Controlled Studies | Cohort Study (Retrospective) | 1212201 | ADHD | ICD Coding | Primary School (Age 6-11) | Preeclampsia |
| Beukers 2017 [12] | Netherlands | Fetal Growth Restriction with Brain Sparing: Neurocognitive and Behavioral Outcomes at 12 Years of Age | Cohort Study (Prospective) | 128 | Overall | Wechsler Intelligence Scale for Children | Adolescent (Age 12-17) | All Hypertensive Disorders/Not Stated |
| Bharadwaj 2018 [13] | India | Oxidative Stress, Antioxidant Status and Neurodevelopmental Outcome in Neonates Born to Pre-Eclamptic Mothers | Cohort Study (Prospective) | 143 | Global Development | Developmental Assessment Scale for Indian Infants | Infant (Age 0-2) | Preeclampsia |
| Bilder 2009 [14] | United States | Prenatal, Perinatal, and Neonatal Factors Associated with Autism Spectrum Disorders | Case-Control Study | 132 | Autism | Screening Tool | Child (Age 3-5) | Gestational Hypertension & Chronic Hypertension |
| Böhm 2019 [15] | United Kingdom | The Effect of Hypertensive Disorders of Pregnancy on the Risk of ADHD in the Offspring | Cohort Study (Prospective) | 13500 | ADHD | Clinical Diagnosis | Primary School (Age 6-11) | All Hypertensive Disorders/Not Stated |
| Bolk 2023 [16] | Sweden | Perinatal Risk Factors for Developmental Coordination Disorder in Children Born Extremely Preterm | Cohort Study (Prospective) | 274 | Global Development | Screening Tool | Child (Age 3-5) | Preeclampsia |
| Brand 2021 [17] | Sweden | Association Between Hypertensive Disorders of Pregnancy and Neurodevelopmental Outcomes Among Offspring | Cohort Study (Retrospective) | 1085024 | Autism, ADHD, Intellectual Disability, Intelligence | ICD Coding | Adult (Age 18+) | All Hypertensive Disorders/Not Stated |
| Buchmayer 2009 [18] | Sweden | Can Association Between Preterm Birth and Autism Be Explained by Maternal or Neonatal Morbidity? | Case-Control Study | 7296 | Autism | ICD Coding | Primary School (Age 6-11) | Preeclampsia & Gestational Hypertension |
| Burstyn 2010 [19] | Canada | Autism Spectrum Disorders, Maternal Characteristics and Obstetric Complications Among Singletons Born in Alberta, Canada | Cohort Study (Retrospective) | 215220 | Autism | Clinical Diagnosis | Multiple Age Groups/Not Stated | Preeclampsia |
| Çak 2013 [20] | Turkey | Attention Deficit Hyperactivity Disorder and Associated Perinatal Risk Factors in Preterm Children | Case-Control Study | 106 | ADHD | Screening Tool | Child (Age 3-5) | All Hypertensive Disorders/Not Stated |
| Carter 2023 [21] | United States | Maternal Obesity, Diabetes, Preeclampsia, and Asthma During Pregnancy and Likelihood of Autism Spectrum Disorder with Gastrointestinal Disturbances in Offspring | Cohort Study (Retrospective) | 308536 | Autism | ICD Coding | Child (Age 3-5) | Preeclampsia |
| Carter 2024 [22] | United States | Preeclampsia Onset, Days to Delivery, and Autism Spectrum Disorders in Offspring: Clinical Birth Cohort Study | Cohort Study (Retrospective) | 364,588 | Autism | ICD Coding | Multiple Age Groups/Not Stated | Preeclampsia |
| Chan 2019 [23] | Canada | Effects of Preeclampsia on Maternal and Pediatric Health at 11 Years Postpartum | Cross-Sectional Study | 41 | Global Development | Vineland-III Assessment | Primary School (Age 6-11) | All Hypertensive Disorders/Not Stated |
| Chang 2023 [24] | Taiwan | Influence of Pre-Eclampsia on 2-Year Neurodevelopmental Outcome of Very-Low-Birth-Weight Infants | Cohort Study (Retrospective) | 482 | Global Development | Bayley Scales of Infant and Toddler Development | Infant (Age 0-2) | Preeclampsia |
| Chen 2020 [25] | China | Impact of Maternal Hypertensive Disorders on Offspring's Neurodevelopment: A Longitudinal Prospective Cohort Study in China | Cohort Study (Prospective) | 4031 | Global Development | Screening Tool | Infant (Age 0-2) | All Hypertensive Disorders/Not Stated |
| Chen 2021 [26] | Taiwan | Childhood Neurodevelopmental Disorders and Maternal Hypertensive Disorder of Pregnancy | Cohort Study (Retrospective) | 877233 | Autism, ADHD, Intellectual Disability, Cerebral Palsy | ICD Coding | Primary School (Age 6-11) | All Hypertensive Disorders/Not Stated |
| Chen 2024 [27] | Japan | Hypertensive Disorders of Pregnancy, Neonatal Outcomes and Offspring Developmental Delay in Japan: The Tohoku Medical Megabank Project Birth and Three-Generation Cohort Study | Cohort Study (Prospective) | 5934 | Global Development | Ages & Stages Questionnaire | Infant (Age 0-2) | All Hypertensive Disorders/Not Stated |
| Cheng 2004 [28] | Taiwan | Delivery Before 32 Weeks of Gestation for Maternal Pre-Eclampsia: Neonatal Outcome and 2-Year Developmental Outcome | Cohort Study (Retrospective) | 89 | Cerebral Palsy & Global Development | Clinical Diagnosis | Infant (Age 0-2) | Preeclampsia |
| Chien 2019 [29] | United States | Prenatal and Perinatal Risk Factors and the Implications on Autism Spectrum Disorder | Cohort Study (Prospective) | 12404 | Autism | Clinical Diagnosis | Multiple Age Groups/Not Stated | Preeclampsia |
| Chowdhury 2023 [30] | United States | Relationship of Nonsteroidal Anti-Inflammatory Drug Use During Pregnancy with Autism Spectrum Disorder and Intellectual Disability Among Offspring | Cohort Study (Retrospective) | 153562 | Autism & Intellectual Disability | Clinical Diagnosis | Multiple Age Groups/Not Stated | Preeclampsia |
| Christians 2022 [31] | United States | Are There Sex Differences in Fetal Growth Strategies and in the Long-Term Effects of Pregnancy Complications on Cognitive Functioning? | Cohort Study (Prospective) | 12404 | Developmental Delay, Intelligence, Education | Bender-Gestalt Test | Primary School (Age 6-11) | Preeclampsia |
| Cochran 2022 [32] | United States | Association of Prenatal Modifiable Risk Factors with Attention-Deficit Hyperactivity Disorder Outcomes at Age 10 and 15 in An Extremely Low Gestational Age Cohort | Cohort Study (Prospective) | 754 | ADHD | Clinical Diagnosis | Adolescent (Age 12-17) | All Hypertensive Disorders/Not Stated |
| Cordero 2019 [33] | United States | Maternal Diabetes and Hypertensive Disorders in Association with Autism Spectrum Disorder | Case-Control Study | 2564 | Autism & Developmental Delay | Screening Tool | Multiple Age Groups/Not Stated | All Hypertensive Disorders/Not Stated |
| Cui 2021 [34] | China | Impact of MicroRNA in Interaction with Environmental Factors on Autism Spectrum Disorder: An Exploratory Pilot Study | Case-Control Study | 318 | Autism | Clinical Diagnosis | Multiple Age Groups/Not Stated | Gestational Hypertension |
| Curran 2017 [35] | United Kingdom | Exposure to Hypertensive Disorders of Pregnancy Increases the Risk of Autism Spectrum Disorder in Affected Offspring | Cohort Study (Prospective) | 13098 | Autism | Clinical Diagnosis | Primary School (Age 6-11) | All Hypertensive Disorders/Not Stated |
| Dachew 2019 [36] | United Kingdom | Pre-Eclampsia and the Risk of Attention-Deficit/Hyperactivity Disorder in Offspring: Findings from the ALSPAC Birth Cohort Study | Cohort Study (Prospective) | 12622 | ADHD | Screening Tool | Primary School (Age 6-11) | Preeclampsia |
| Dachew 2021 [37] | United Kingdom | Maternal Hypertensive Disorders During Pregnancy and the Trajectories of Offspring Emotional and Behavioral Problems: the ALSPAC Birth Cohort Study | Cohort Study (Prospective) | 10185 | Developmental Delay | Strengths & Difficulties Questionnaire | Multiple Age Groups/Not Stated | Preeclampsia & Gestational Hypertension |
| Dodds 2010 [38] | Canada | The Role of Prenatal, Obstetric and Neonatal Factors in the Development of Autism | Cohort Study (Retrospective) | 129733 | Autism | Clinical Diagnosis | Multiple Age Groups/Not Stated | Gestational Hypertension |
| Duko 2024 [39] | Australia | The Effects of Pre-Eclampsia on Social and Emotional Developmental Vulnerability at Age Five in Western Australia: A Population Linkage Study | Cohort Study (Retrospective) | 64392 | Global Development | Australian Early Development Census | Multiple Age Groups/Not Stated | Preeclampsia |
| Ehrenstein 2009 [40] | Denmark | Pregnancy-Associated Hypertensive Disorders and Adult Cognitive Function Among Danish Conscripts | Cohort Study (Retrospective) | 17457 | Intelligence | Danish Draft Board Intelligence Test | Adult (Age 18+) | Preeclampsia & Gestational Hypertension |
| Fast 2024 [41] | Sweden | Prevalence of Attention-Deficit/ Hyperactivity Disorder and Autism in 12-Year-Old Children: A Population-Based Cohort | Cohort Study (Prospective) | 2658 | Autism & ADHD | Database | Adolescent (Age 12-17) | Preeclampsia |
| Fitton 2021 [42] | United Kingdom | Congenital Defects and Early Childhood Outcomes Following in-Utero Exposure to Antihypertensive Medication | Cohort Study (Retrospective) | 265488 | Global Development | Ages & Stages Questionnaire | Child (Age 3-5) | All Hypertensive Disorders/Not Stated |
| Getahun 2013 [43] | United States | In Utero Exposure to Ischemic-Hypoxic Conditions and Attention-Deficit/Hyperactivity Disorder | Case-Control Study | 81678 | ADHD | ICD Coding | Multiple Age Groups/Not Stated | Preeclampsia |
| Girchenko 2018a [44] | Finland | Maternal Early Pregnancy Obesity and Related Pregnancy and Pre-Pregnancy Disorders: Associations with Child Developmental Milestones in the Prospective PREDO Study | Case-Control Study | 81678 | Global Development | Ages & Stages Questionnaire | Multiple Age Groups/Not Stated | All Hypertensive Disorders/Not Stated |
| Girchenko 2018b [45] | Finland | Neonatal Regulatory Behavior Problems Are Predicted by Maternal Early Pregnancy Overweight and Obesity: Findings from the Prospective PREDO Study | Case-Control Study | 2116 | Global Development | Ages & Stages Questionnaire | Infant (Age 0-2) | All Hypertensive Disorders/Not Stated |
| Glasson 2004 [46] | Australia | Perinatal Factors and the Development of Autism: A Population Study | Cohort Study (Retrospective) | 1778 | Autism | Clinical Diagnosis | Multiple Age Groups/Not Stated | Preeclampsia |
| Golmirzaei 2013 [47] | Iran | Evaluation of Attention-Deficit Hyperactivity Disorder Risk Factors | Case-Control Study | 404 | ADHD | Screening Tool | Primary School (Age 6-11) | Preeclampsia |
| Gray 1997 [48] | Australia | Survival and Neurodevelopmental Outcome of 24-26 Week Gestation Infants According to Primary Cause of Preterm Delivery | Cohort Study (Retrospective) | 189 | Cerebral Palsy | Clinical Diagnosis | Infant (Age 0-2) | Preeclampsia |
| Gray 1998 [49] | Australia | Maternal Hypertension and Neurodevelopmental Outcome in Very Preterm Infants | Cohort Study (Prospective) | 214 | Developmental Delay | Griffiths’ Infant Ability Scale | Infant (Age 0-2) | All Hypertensive Disorders/Not Stated |
| Griffith 2011 [50] | United States | The Risk of Intellectual Disability in Children Born to Mothers with Preeclampsia or Eclampsia with Partial Mediation by Low Birth Weight | Cohort Study (Retrospective) | 80866 | Intellectual Disability | Not Stated | Multiple Age Groups/Not Stated | Preeclampsia |
| He 2017 [51] | China | Analysis of High-Risk Factors and Effect of Early Intervention on Preterm Infant Neurodevelopment | Cohort Study (Prospective) | 1337 | Developmental Delay | Screening Tool | Infant (Age 0-2) | All Hypertensive Disorders/Not Stated |
| Heikura 2013 [52] | Finland | Maternal Hypertensive Disorders During Pregnancy and Mild Cognitive Limitations in the Offspring | Cohort Study (Prospective) | 8847 | Global Development | Screening Tool | Primary School (Age 6-11) | All Hypertensive Disorders/Not Stated |
| Hisle-Gormon 2018 [53] | United States | Prenatal, Perinatal, and Neonatal Risk Factors of Autism Spectrum Disorder | Case-Control Study | 35040 | Autism | ICD Coding | Multiple Age Groups/Not Stated | All Hypertensive Disorders/Not Stated |
| Huang 2023 [54] | China | Association of Maternal Hypertensive Disorders in Pregnancy with Infant Neurodevelopment | Cohort Study (Prospective) | 2837 | Global Development | Bayley Scales of Infant and Toddler Development | Infant (Age 0-2) | All Hypertensive Disorders/Not Stated |
| Hultman 2002 [55] | Sweden | Perinatal Risk Factors for Infantile Autism | Case-Control Study | 2448 | Autism | Clinical Diagnosis | Multiple Age Groups/Not Stated | All Hypertensive Disorders/Not Stated |
| Ishikuro 2021 [56] | Japan | Hypertension in Pregnancy as A Possible Factor for Child Autistic Behavior at Two Years Old | Cohort Study (Prospective) | 6794 | Autism | Screening Tool | Infant (Age 0-2) | All Hypertensive Disorders/Not Stated |
| Kodesh 2022 [57] | Israel | Maternal Health Around Pregnancy and Autism Risk: A Diagnosis-Wide, Population-Based Study | Case-Control Study | 80187 | Autism | ICD Coding | Multiple Age Groups/Not Stated | All Hypertensive Disorders/Not Stated |
| Kong 2022 [58] | Finland | Association of Preeclampsia and Perinatal Complications with Offspring Neurodevelopmental and Psychiatric Disorders | Cohort Study (Retrospective) | 1012723 | Autism, ADHD, Intellectual Disability, Developmental Delay | ICD Coding | Multiple Age Groups/Not Stated | Preeclampsia |
| Koparkar 2022 [59] | India | Cognitive Function and Behavioral Problems in Children Born to Mothers with Preeclampsia: An Indian Study | Cohort Study (Prospective) | 308 | Global Development & Developmental Delay | Strengths & Difficulties Questionnaire | Multiple Age Groups/Not Stated | Preeclampsia |
| Korzeniewski 2013 [60] | United States | Association Between Transient Hypothyroxinemia of Pregnancy and Adult Autism Spectrum Disorder in A Low Birthweight Cohort: An Exploratory Study | Cohort Study (Prospective) | 1105 | Autism | Screening Tool | Adult (Age 18+) | All Hypertensive Disorders/Not Stated |
| Krakowiak 2012 [61] | United States | Maternal Metabolic Conditions and Risk for Autism and Other Neurodevelopmental Disorders | Case-Control Study | 1004 | Autism & Developmental Delay | Screening Tool | Child (Age 3-5) | All Hypertensive Disorders/Not Stated |
| Lahti-Pulkkinen 2020 [62] | Finland | Maternal Hypertensive Pregnancy Disorders and Mental Disorders in Children | Cohort Study (Prospective) | 4743 | Developmental Delay | ICD Coding | Primary School (Age 6-11) | All Hypertensive Disorders/Not Stated |
| Larsson 2005 [63] | Denmark | Risk Factors for Autism: Perinatal Factors, Parental Psychiatric History, and Socioeconomic Status | Case-Control Study | 18148 | Autism | Clinical Diagnosis | Multiple Age Groups/Not Stated | Preeclampsia |
| Lee 2023 [64] | South Korea | Impact of Moderate-to-Late Preterm Birth on Neurodevelopmental Outcomes in Young Children: Results from Retrospective Longitudinal Follow-Up with Nationally Representative Data | Cohort Study (Retrospective) | 732,757 | Global Development | Screening Tool | Multiple Age Groups/Not Stated | Gestational Hypertension |
| Leitner 2012 [65] | Israel | The Neurocognitive Outcome of IUGR Children Born to Mothers with and without Preeclampsia | Cohort Study (Prospective) | 120 | Intelligence | Wechsler Intelligence Scale for Children | Primary School (Age 6-11) | Preeclampsia & Gestational Hypertension |
| Leonard 2006 [66] | Australia | Maternal Health in Pregnancy and Intellectual Disability in the Offspring: A Population-Based Study | Cohort Study (Retrospective) | 239426 | Intellectual Disability | Clinical Diagnosis | Multiple Age Groups/Not Stated | All Hypertensive Disorders/Not Stated |
| Liu 2020 [67] | China | Reduced Intellectual Ability in Offspring Born from Preeclamptic Mothers: A Prospective Cohort Study | Cohort Study (Prospective) | 303 | Intelligence | Wechsler Intelligence Scale for Children | Child (Age 3-5) | Preeclampsia |
| Lyall 2022 [68] | United States | Cardiometabolic Pregnancy Complications in Association with Autism-Related Traits as Measured by the Social Responsiveness Scale in ECHO | Cohort Study (Prospective) | 6778 | Autism | Screening Tool | Primary School (Age 6-11) | All Hypertensive Disorders/Not Stated |
| Maher 2020a [69] | Sweden | Association Between Preeclampsia and Autism Spectrum Disorder: A Population-Based Study | Cohort Study (Retrospective) | 2842230 | Autism | ICD Coding | Multiple Age Groups/Not Stated | Preeclampsia |
| Maher 2020b [70] | Ireland | The Association Between Preeclampsia and Childhood Development and Behavioural Outcomes | Cohort Study (Prospective) | 70791 | Global Development & Developmental Delay | Ages & Stages Questionnaire | Infant (Age 0-2) | Preeclampsia |
| Maher 2020c [9] | Sweden | The Association Between Preeclampsia and Attention-Deficit/Hyperactivity Disorder: A Population-Based and Sibling-Matched Cohort Study | Cohort Study (Retrospective) | 2047619 | ADHD | ICD Coding | Multiple Age Groups/Not Stated | Preeclampsia |
| Maher 2021 [71] | United Kingdom | Hypertensive Disorders of Pregnancy and Behavioural Outcomes in the Offspring: Findings from the Millennium Cohort Study | Cohort Study (Prospective) | 18274 | Global Development & Developmental Delay | Strengths & Difficulties Questionnaire | Primary School (Age 6-11) | All Hypertensive Disorders/Not Stated |
| Mann 2010 [72] | United States | Pre-Eclampsia, Birth Weight, and Autism Spectrum Disorders | Cohort Study (Retrospective) | 87677 | Autism | ICD Coding | Multiple Age Groups/Not Stated | Preeclampsia |
| Mann 2011a [73] | United States | Are Maternal Genitourinary Infection and Pre-Eclampsia Associated with ADHD in School-Aged Children? | Cohort Study (Retrospective) | 84721 | ADHD | ICD Coding | Multiple Age Groups/Not Stated | Preeclampsia |
| Mann 2011b [74] | United States | Uncovering the Complex Relationship Between Pre-Eclampsia, Preterm Birth and Cerebral Palsy | Cohort Study (Retrospective) | 122476 | Cerebral Palsy | ICD Coding | Multiple Age Groups/Not Stated | Preeclampsia |
| Manovitch 2022 [75] | Israel | Neurodevelopmental Outcomes of Preterm Infants Born to Preeclamptic Mothers - A Case-Control Study | Case-Control Study | 78 | Developmental Delay | Griffiths’ Mental Development Scales | Infant (Age 0-2) | Preeclampsia |
| Many 2003 [76] | Israel | Neurodevelopmental and Cognitive Assessment of Children Born Growth Restricted to Mothers with and without Preeclampsia | Cohort Study (Prospective) | 75 | Intelligence | Stanford-Binet Test | Child (Age 3-5) | Preeclampsia |
| Many 2005 [77] | Israel | Neurodevelopmental and Cognitive Assessment of 6-Year-Old Children Born Growth Restricted | Cohort Study (Prospective) | 92 | Intelligence | Wechsler Preschool & Primary Scale of Intelligence | Primary School (Age 6-11) | All Hypertensive Disorders/Not Stated |
| Matić 2017 [78] | Australia | Maternal Hypertensive Disorders Are Associated with Increased Use of Respiratory Support but not Chronic Lung Disease or Poorer Neurodevelopmental Outcomes in Preterm Neonates at <29 Weeks of Gestation | Cohort Study (Retrospective) | 2549 | Cerebral Palsy & Developmental Delay | Screening Tool | Child (Age 3-5) | All Hypertensive Disorders/Not Stated |
| McCowan 2002 [79] | New Zealand | Perinatal Predictors of Neurodevelopmental Outcome in Small-for-Gestational-Age Children at 18 Months of Age | Cohort Study (Prospective) | 282 | Global Development | Bayley Scales of Infant and Toddler Development | Infant (Age 0-2) | All Hypertensive Disorders/Not Stated |
| Moore 2012 [80] | United States | Autism Risk in Small- and Large-for-Gestational-Age Infants | Cohort Study (Retrospective) | 5979605 | Autism | Clinical Diagnosis | Multiple Age Groups/Not Stated | Preeclampsia & Chronic Hypertension |
| Mor 2016 [81] | Israel | Early Onset Preeclampsia and Cerebral Palsy: A Double Hit Model? | Cohort Study (Retrospective) | 229192 | Cerebral Palsy | ICD Coding | Child (Age 3-5) | Preeclampsia |
| Morsing 2014 [82] | Sweden | Pre-Eclampsia - An Additional Risk Factor for Cognitive Impairment at School Age After Intrauterine Growth Restriction and Very Preterm Birth | Case-Control Study | 68 | Cerebral Palsy & Intelligence | Not Stated | Infant (Age 0-2) | Preeclampsia |
| Mrozek-Budzyn 2013 [83] | Poland | Prenatal, Perinatal and Neonatal Risk Factors for Autism - Study in Poland | Case-Control Study | 288 | Autism | Clinical Diagnosis | Primary School (Age 6-11) | Preeclampsia & Chronic Hypertension |
| Murphy 1995 [84] | United Kingdom | Case-Control Study of Antenatal and Intrapartum Risk Factors for Cerebral Palsy in Very Preterm Singleton Babies | Case-Control Study | 293 | Cerebral Palsy | Clinical Diagnosis | Child (Age 3-5) | Preeclampsia & Chronic Hypertension |
| Nahum-Sacks 2019 [85] | Israel | Long-Term Neuropsychiatric Morbidity in Children Exposed Prenatally to Preeclampsia | Cohort Study (Retrospective) | 253808 | Autism & Cerebral Palsy | ICD Coding | Multiple Age Groups/Not Stated | Preeclampsia |
| Nath 2012 [86] | India | Perinatal Complications Associated with Autism--A Case Control Study in A Neurodevelopment and Early Intervention Clinic | Case-Control Study | 131 | Autism | Clinical Diagnosis | Child (Age 3-5) | Gestational Hypertension |
| Nielson 2024 [87] | Australia | Association Between Cumulative Maternal Exposures Related to Inflammation and Child Attention-Deficit/Hyperactivity Disorder: A Cohort Study | Cohort Study (Retrospective) | 908770 | ADHD | Database | Multiple Age Groups/Not Stated | All Hypertensive Disorders/Not Stated |
| Noda 2021 [88] | Japan | Association Between Maternal Hypertensive Disorders of Pregnancy and Child Neurodevelopment at 3 Years of Age: A Retrospective Cohort Study | Cohort Study (Retrospective) | 43854 | Global Development | Clinical Diagnosis | Child (Age 3-5) | All Hypertensive Disorders/Not Stated |
| Palatnik 2022 [89] | United States | Association Between Hypertensive Disorders of Pregnancy and Long-Term Neurodevelopmental Outcomes in the Offspring | Secondary Analysis of a Clinical Trial | 1179 | Global Development & Intelligence | Child Behaviour Checklist | Child (Age 3-5) | All Hypertensive Disorders/Not Stated |
| Palmer 1995 [90] | Australia | Antenatal Antecedents of Moderate and Severe Cerebral Palsy | Case-Control Study | 860 | Cerebral Palsy | Database | Multiple Age Groups/Not Stated | Preeclampsia |
| Pohlabeln 2017 [91] | Europe (Multi-Country) | Further Evidence for the Role of Pregnancy-Induced Hypertension and Other Early Life Influences in the Development of ADHD: Results from the IDEFICS Study | Cohort Study (Prospective) | 15577 | ADHD | Screening Tool | Multiple Age Groups/Not Stated | Gestational Hypertension |
| Polo-Kantola 2014 [92] | Finland | Obstetric Risk Factors and Autism Spectrum Disorders in Finland | Case-Control Study | 4713 | Autism | ICD Coding | Multiple Age Groups/Not Stated | All Hypertensive Disorders/Not Stated |
| Raz 2015 [93] | United States | Autism Spectrum Disorder and Particulate Matter Air Pollution Before, During, and After Pregnancy: A Nested Case-Control Analysis within the Nurses' Health Study II Cohort | Case-Control Study | 1767 | Autism | Clinical Diagnosis | Multiple Age Groups/Not Stated | Preeclampsia |
| Robinson 2009 [94] | Australia | Hypertensive Diseases of Pregnancy and the Development of Behavioural Problems in Childhood and Adolescence: The Western Australian Pregnancy Cohort Study | Cohort Study (Prospective) | 2979 | Behaviour | Child Behaviour Checklist | Primary School (Age 6-11) | Preeclampsia & Gestational Hypertension |
| Sabino 2017 [95] | Brazil | High Blood Pressure during Pregnancy is not a Protective Factor for Preterm Infants with Very Low Birth Weight: A Case-Control Study | Case-Control | 93 | Global Development | Denver II Developmental Screening Test | Infant (Age 0-2) | Preeclampsia & Chronic Hypertension |
| Scime 2021 [96] | Canada | Hypertensive Disorders in Pregnancy and Child Development at 36 months in the All Our Families Prospective Cohort Study | Cohort Study (Prospective) | 1554 | Global Development & Developmental Delay | Ages & Stages Questionnaire | Infant (Age 0-2) | All Hypertensive Disorders/Not Stated |
| Seidman 1991 [97] | Israel | Pre-Eclampsia and Offspring's Blood Pressure, Cognitive Ability and Physical Development at 17-Years-of-Age | Cohort Study (Retrospective) | 33545 | Intelligence | Wechsler Adult Intelligence Scale | Adolescent (Age 12-17) | Preeclampsia |
| Selvaratnam 2022 [98] | Australia | Childhood School Outcomes for Infants Born to Women with Hypertensive Disorders During Pregnancy | Cohort Study (Retrospective) | 682386 | Developmental Delay & Education | Australian Early Development Census & Standardised Testing | Child (Age 3-5) | All Hypertensive Disorders/Not Stated |
| Silveira 2007 [99] | Brazil | Growth and Neurodevelopment Outcome of Very Low Birth Weight Infants Delivered by Preeclamptic Mothers | Cohort Study (Prospective) | 86 | Global Development | Screening Tool | Infant (Age 0-2) | Preeclampsia |
| Spinillo 2006 [100] | Italy | Rates of Neonatal Death and Cerebral Palsy Associated with Fetal Growth Restriction Among Very Low Birthweight Infants. A Temporal Analysis | Cohort Study (Retrospective) | 773 | Cerebral Palsy & Developmental Delay | Clinical Diagnosis | Infant (Age 0-2) | Preeclampsia |
| Spinillo 2009 [101] | Italy | Infant Sex, Obstetric Risk Factors, and 2-Year Neurodevelopmental Outcome Among Preterm Infants | Cohort Study (Prospective) | 754 | Developmental Delay | Screening Tool | Infant (Age 0-2) | Preeclampsia |
| Sun 2020 [102] | Norway | Association of Preeclampsia in Term Births with Neurodevelopmental Disorders in Offspring | Cohort Study (Prospective) | 980560 | Autism, ADHD, Intellectual Disability, Cerebral Palsy | ICD Coding | Multiple Age Groups/Not Stated | Preeclampsia |
| Sverrisson 2018 [103] | Iceland | Preeclampsia and Academic Performance in Children: A Nationwide Study from Iceland | Cohort Study (Retrospective) | 63014 | ADHD & Education | Standardised Testing | Multiple Age Groups/Not Stated | Preeclampsia |
| Szymonowicz 1987 [104] | Australia | Severe Pre-Eclampsia and Infants of Very Low Birth Weight | Cohort Study (Retrospective) | 53 | Cerebral Palsy, Developmental Delay, Global Development | Clinical Diagnosis | Infant (Age 0-2) | Preeclampsia |
| Taylor 1985 [105] | United Kingdom | Do Pregnancy Complications Contribute to Neurodevelopmental Disability? | Cohort Study (Prospective) | 4852 | Developmental Delay, Cerebral Palsy, Intellectual Disability | Not Stated | Infant (Age 0-2) | All Hypertensive Disorders/Not Stated |
| Tuovinen 2012 [106] | Finland | Hypertensive Disorders in Pregnancy and Cognitive Decline in the Offspring Up to Old Age | Cohort Study (Prospective) | 1196 | Intelligence | Finnish Defence Forces Basic Ability Test | Adult (Age 18+) | All Hypertensive Disorders/Not Stated |
| Ushida 2020 [107] | Japan | Antenatal Corticosteroids and Preterm Offspring Outcomes in Hypertensive Disorders of Pregnancy: A Japanese Cohort Study | Cohort Study (Retrospective) | 21014 | Cerebral Palsy | Not Stated | Child (Age 3-5) | All Hypertensive Disorders/Not Stated |
| Villamor 2022a [108] | Sweden | Defective Placentation Syndromes and Autism Spectrum Disorder in the Offspring: Population-Based Cohort and Sibling-Controlled Studies | Cohort Study (Retrospective) | 1645455 | Autism | ICD Coding | Multiple Age Groups/Not Stated | Preeclampsia |
| Villamor 2022b [109] | Sweden | Defective Placentation Syndromes and Intellectual Disability in the Offspring: Nationwide Cohort and Sibling-Controlled Studies | Cohort Study (Retrospective) | 1581200 | Intellectual Disability | ICD Coding | Multiple Age Groups/Not Stated | Preeclampsia |
| Walker 2015 [110] | United States | Preeclampsia, Placental Insufficiency, and Autism Spectrum Disorder or Developmental Delay | Case-Control Study | 1061 | Autism & Developmental Delay | ICD Coding | Multiple Age Groups/Not Stated | Preeclampsia & Chronic Hypertension |
| Wang 2021 [111] | Europe (Multi-Country) | Maternal Hypertensive Disorders and Neurodevelopmental Disorders in Offspring: A Population-Based Cohort in Two Nordic Countries | Cohort Study (Retrospective) | 4489044 | Autism, ADHD, Intellectual Disability | ICD Coding | Multiple Age Groups/Not Stated | All Hypertensive Disorders/Not Stated |
| Wang 2023a [112] | Taiwan | Maternal Hypertensive Pregnancy Disorders Increase Childhood Intellectual Disability Hazards Independently from Preterm Birth and Small for Gestational Age | Cohort Study (Retrospective) | 116742 | Intellectual Disability | ICD Coding | Multiple Age Groups/Not Stated | All Hypertensive Disorders/Not Stated |
| Wang 2023b [113] | Taiwan | Preterm Birth and Small for Gestational Age Potentiate the Association Between Maternal Hypertensive Pregnancy and Childhood Autism Spectrum Disorder | Cohort Study (Retrospective) | 108786 | Autism | ICD Coding | Multiple Age Groups/Not Stated | All Hypertensive Disorders/Not Stated |
| Wang 2024 [114] | China | Prenatal Environmental Adversity and Child Neurodevelopmental Delay: The Role of Maternal Low-Grade Systemic Inflammation and Maternal Anti-Inflammatory Diet | Cohort Study (Prospective) | 7438 | Global Development | Database | Multiple Age Groups/Not Stated | All Hypertensive Disorders/Not Stated |
| Warshafsky 2016 [115] | United Kingdom | Prospective Assessment of Neurodevelopment in Children Following A Pregnancy Complicated by Severe Pre-Eclampsia | Cohort Study (Prospective) | 269 | Developmental Delay | Ages & Stages Questionnaire | Primary School (Age 6-11) | Preeclampsia |
| Whitehouse 2012 [116] | Australia | Do Hypertensive Diseases of Pregnancy Disrupt Neurocognitive Development in Offspring? | Cohort Study (Prospective) | 1389 | Intelligence | Peabody Picture Vocabulary Test | Primary School (Age 6-11) | Preeclampsia & Gestational Hypertension |
| Whitely 2022 [117] | Australia | Brief Report: Pregnancy, Birth, and Infant Feeding Practices: A Survey-Based Investigation Into Risk Factors for Autism Spectrum Disorder | Cross-Sectional Study | 3158 | Autism | Parental Assessment | Multiple Age Groups/Not Stated | Preeclampsia |
| Wiggs 2024 [118] | United States | Setting a Research Agenda for Examining Early Risk for Elevated Cognitive Disengagement Syndrome Symptoms Using Data from the ABCD Cohort | Cohort Study (Prospective) | 8096 | Global Development | Child Behavior Checklist | Primary School (Age 6-11) | Preeclampsia & Gestational Hypertension |
| Wilson-Costello 1998 [119] | United States | Perinatal Correlates of Cerebral Palsy and Other Neurologic Impairment Among Very Low Birth Weight Children | Case-Control Study | 144 | Cerebral Palsy | Clinical Diagnosis | Infant (Age 0-2) | All Hypertensive Disorders/Not Stated |
| Winer 1982 [120] | United States | Four- to Seven-Year Evaluation in Two Groups of Small-for-Gestational Age Infants | Cross-Sectional Study | 55 | Intelligence | Wechsler Preschool & Primary Scale of Intelligence | Primary School (Age 6-11) | All Hypertensive Disorders/Not Stated |
| Withagen 2005 [121] | Netherlands | Morbidity and Development in Childhood of Infants Born After Temporising Treatment of Early Onset Pre-Eclampsia | Case-Control Study | 574 | Cerebral Palsy, Developmental Delay, Intellectual Disability, Global Development | Screening Tool | Multiple Age Groups/Not Stated | Preeclampsia |
| Zen 2021 [122] | Australia | Perinatal and Child Factors Mediate the Association Between Preeclampsia and Offspring School Performance | Cohort Study (Retrospective) | 43600 | Education | Standardised Testing | Primary School (Age 6-11) | Preeclampsia |

**Table D. Characteristics of Excluded Studies**

| **Study ID** | **Title** | **Country** | **Design** | **Outcome** | **Reason for Exclusion** |
| --- | --- | --- | --- | --- | --- |
| Barker 1967 [123] | Obstetric Complications and School Performance | United Kingdom | Cohort Study (Retrospective) | Education | Data in incorrect format for meta-analysis; authors could not be contacted. |
| Check 2024 [124] | Preeclampsia, Fetal Growth Restriction, and 24-Month Neurodevelopment in Very Preterm Infants | United States | Cohort Study (Prospective) | Global Development | Data in incorrect format for meta-analysis; authors did not respond. |
| Grace 2014 [125] | Maternal Hypertensive Diseases Negatively Affect Offspring Motor Development | Australia | Cohort Study (Prospective) | Global Development | Data in incorrect format for meta-analysis; authors did not respond. |
| Iwabuchi 2022 [126] | Associations among maternal metabolic conditions, cord serum leptin levels, and autistic symptoms in children | Japan | Cohort Study (Prospective) | Autism Spectrum Disorder | Data in incorrect format for meta-analysis; authors did not respond. |
| Li 2016 [127] | Etiological Subgroups of Small-for-Gestational-Age: Differential Neurodevelopmental Outcomes | United States | Cohort Study (Prospective) | Education | Data in incorrect format for meta-analysis; authors could not be contacted. |
| Ma 2023 [128] | Association of maternal hypertension during pregnancy with brain structure and behavioral problems in early adolescence | United States | Cohort Study (Prospective) | Global Development | Data in incorrect format for meta-analysis; authors did not respond. |
| Ratsep 2016 [129] | Impact of Preeclampsia on Cognitive Function in the Offspring | Canada | Case-Control | Intelligence | Missing data; authors did not respond. |
| Schlapbach 2010 [130] | Impact of Chorioamnionitis and Preeclampsia on Neurodevelopmental Outcome in Preterm Infants Below 32 Weeks Gestational Age | Switzerland | Case-Control | Global Development | Data in incorrect format for meta-analysis; authors did not respond. |
| Spinillo 1993 [131] | Infant Neurodevelopmental Outcome in Pregnancies Complicated by Gestational Hypertension and Intra-Uterine Growth Retardation | Italy | Cohort Study (Prospective) | Global Development | Data in incorrect format for meta-analysis; authors did not respond. |
| Yoneda 2021 [132] | Pre-eclampsia Complicated with Maternal Renal Dysfunction is Associated with Poor Neurological Development at 3 Years Old in Children Born Before 34 Weeks of Gestation | Japan | Cohort Study (Retrospective) | Global Development | Data presented as composite outcome; authors did not respond. |

**Table E. Risk of bias assessment**

**Newcastle-Ottawa Scale (NOS) for assessing the quality of nonrandomized studies in meta-analyses**

| **Study ID** | **Selection (/4)** | **Comparability (/2)** | **Exposure/ Outcome (/3)** | **Overall Rating** |
| --- | --- | --- | --- | --- |
| Abdelmageed 2024 [1] | 3 | 2 | 2 | Good |
| Almasri 2021 [2] | 4 | 1 | 3 | Good |
| Amiri 2012 [3] | 3 | 0 | 0 | Poor |
| Arafa 2022 [4] | 3 | 1 | 1 | Fair |
| Arun 2023 [5] | 4 | 1 | 3 | Good |
| Avorbegdor 2019 [7] | 3 | 1 | 2 | Good |
| Ayala 2021 [8] | 4 | 1 | 3 | Good |
| Bajalan 2020 [10] | 4 | 1 | 1 | Fair |
| Beer 2022 [11] | 4 | 1 | 3 | Good |
| Beukers 2017 [12] | 3 | 1 | 3 | Good |
| Bharadwaj 2018 [13] | 4 | 0 | 3 | Good |
| Bilder 2009 [14] | 4 | 1 | 3 | Good |
| Böhm 2019 [15] | 3 | 0 | 1 | Fair |
| Bolk 2023 [16] | 3 | 0 | 2 | Fair |
| Brand 2021 [17] | 4 | 1 | 3 | Good |
| Buchmayer 2009 [18] | 4 | 1 | 3 | Good |
| Burstyn 2010 [19] | 4 | 1 | 3 | Good |
| Çak 2013 [20] | 4 | 1 | 3 | Good |
| Carter 2023 [21] | 4 | 1 | 3 | Good |
| Carter 2024 [22] | 3 | 1 | 2 | Fair |
| Chan 2019 [23] | 2 | 0 | 3 | Fair |
| Chang 2023 [24] | 4 | 1 | 3 | Good |
| Chen 2020 [25] | 4 | 1 | 3 | Good |
| Chen 2021 [26] | 4 | 1 | 3 | Good |
| Chen 2024 [27] | 3 | 2 | 1 | Fair |
| Cheng 2004 [28] | 2 | 0 | 3 | Fair |
| Chien 2019 [29] | 3 | 0 | 0 | Poor |
| Chowdhury 2023 [30] | 2 | 1 | 2 | Fair |
| Christians 2022 [31] | 4 | 1 | 3 | Good |
| Cochran 2022 [32] | 4 | 1 | 3 | Good |
| Cordero 2019 [33] | 4 | 1 | 2 | Good |
| Cui 2021 [34] | 2 | 0 | 1 | Poor |
| Curran 2017 [35] | 3 | 1 | 3 | Good |
| Dachew 2019 [36] | 4 | 2 | 2 | Good |
| Dachew 2021 [37] | 3 | 1 | 3 | Good |
| Dodds 2010 [38] | 4 | 1 | 3 | Good |
| Duko 2024 [39] | 3 | 1 | 2 | Fair |
| Ehrenstein 2009 [40] | 3 | 1 | 3 | Good |
| Fast 2024 [41] | 4 | 2 | 2 | Good |
| Fitton 2021 [42] | 4 | 1 | 3 | Good |
| Getahun 2013 [43] | 3 | 1 | 2 | Fair |
| Girchenko 2018a [44] | 3 | 1 | 3 | Good |
| Girchenko 2018b [45] | 3 | 1 | 1 | Fair |
| Glasson 2004 [46] | 4 | 1 | 3 | Good |
| Golmirzaei 2013 [47] | 3 | 0 | 1 | Fair |
| Gray 1997 [48] | 4 | 0 | 3 | Good |
| Gray 1998 [49] | 4 | 1 | 3 | Good |
| Griffith 2011 [50] | 4 | 1 | 3 | Good |
| He 2017 [51] | 3 | 1 | 2 | Fair |
| Heikura 2013 [52] | 4 | 1 | 3 | Good |
| Hisle-Gormon 2018 [53] | 3 | 1 | 3 | Good |
| Huang 2023 [54] | 2 | 1 | 2 | Fair |
| Hultman 2002 [55] | 4 | 1 | 3 | Good |
| Ishikuro 2021 [56] | 4 | 1 | 3 | Good |
| Kodesh 2022 [57] | 4 | 1 | 3 | Good |
| Kong 2022 [58] | 4 | 1 | 3 | Good |
| Koparkar 2022 [59] | 4 | 1 | 3 | Good |
| Korzeniewski 2013 [60] | 2 | 0 | 3 | Fair |
| Krakowiak 2012 [61] | 4 | 1 | 1 | Fair |
| Lahti-Pulkkinen 2020 [62] | 4 | 1 | 3 | Good |
| Larsson 2005 [63] | 4 | 1 | 2 | Good |
| Lee 2023 [64] | 4 | 2 | 3 | Good |
| Leitner 2012 [65] | 4 | 0 | 3 | Good |
| Leonard 2006 [66] | 3 | 1 | 1 | Fair |
| Liu 2020 [67] | 4 | 1 | 3 | Good |
| Lyall 2022 [68] | 4 | 1 | 3 | Good |
| Maher 2020a [69] | 4 | 1 | 3 | Good |
| Maher 2020b [70] | 3 | 1 | 3 | Good |
| Maher 2020c [9] | 4 | 1 | 3 | Good |
| Maher 2021 [71] | 3 | 1 | 3 | Good |
| Mann 2010 [72] | 4 | 1 | 3 | Good |
| Mann 2011a [73] | 4 | 1 | 3 | Good |
| Mann 2011b [74] | 4 | 1 | 3 | Good |
| Manovitch 2022 [75] | 4 | 1 | 2 | Good |
| Many 2003 [76] | 4 | 0 | 3 | Good |
| Many 2005 [77] | 1 | 0 | 3 | Fair |
| Matić 2017 [78] | 4 | 1 | 3 | Good |
| McCowan 2002 [79] | 3 | 0 | 3 | Fair |
| Moore 2012 [80] | 4 | 1 | 3 | Good |
| Mor 2016 [81] | 4 | 2 | 3 | Good |
| Morsing 2014 [82] | 4 | 1 | 1 | Fair |
| Mrozek-Budzyn 2013 [83] | 3 | 0 | 1 | Fair |
| Murphy 1995 [84] | 4 | 2 | 2 | Good |
| Nahum-Sacks 2019 [85] | 4 | 1 | 3 | Good |
| Nath 2012 [86] | 3 | 1 | 2 | Fair |
| Nielson 2024 [87] | 4 | 1 | 2 | Good |
| Noda 2021 [88] | 4 | 1 | 3 | Good |
| Palatnik 2022 [89] | 3 | 2 | 3 | Good |
| Palmer 1995 [90] | 3 | 1 | 3 | Good |
| Pohlabeln 2017 [91] | 3 | 1 | 3 | Good |
| Polo-Kantola 2014 [92] | 4 | 1 | 3 | Good |
| Raz 2015 [93] | 3 | 1 | 3 | Good |
| Robinson 2009 [94] | 4 | 1 | 3 | Good |
| Sabino 2017 [95] | 2 | 0 | 2 | Fair |
| Scime 2021 [96] | 4 | 2 | 3 | Good |
| Seidman 1991 [97] | 4 | 1 | 3 | Good |
| Selvaratnam 2022 [98] | 4 | 1 | 3 | Good |
| Silveira 2007 [99] | 3 | 0 | 3 | Fair |
| Spinillo 2006 [100] | 3 | 1 | 3 | Good |
| Spinillo 2009 [101] | 3 | 2 | 3 | Good |
| Sun 2020 [102] | 4 | 1 | 3 | Good |
| Sverrisson 2018 [103] | 4 | 1 | 3 | Good |
| Szymonowicz 1987 [104] | 3 | 1 | 1 | Fair |
| Taylor 1985 [105] | 4 | 1 | 1 | Fair |
| Tuovinen 2012 [106] | 3 | 2 | 3 | Good |
| Ushida 2020 [107] | 3 | 1 | 3 | Good |
| Villamor 2022a [108] | 4 | 1 | 3 | Good |
| Villamor 2022b [109] | 4 | 1 | 3 | Good |
| Walker 2015 [110] | 4 | 1 | 2 | Good |
| Wang 2021 [111] | 4 | 1 | 3 | Good |
| Wang 2023a [112] | 3 | 2 | 3 | Good |
| Wang 2023b [113] | 4 | 2 | 3 | Good |
| Wang 2024 [114] | 4 | 2 | 1 | Good |
| Warshafsky 2016 [115] | 4 | 1 | 3 | Good |
| Whitehouse 2012 [116] | 4 | 2 | 3 | Good |
| Whitely 2022 [117] | 2 | 1 | 3 | Fair |
| Wiggs 2024 [118] | 3 | 0 | 1 | Fair |
| Wilson-Costello 1998 [119] | 4 | 1 | 1 | Fair |
| Winer 1982 [120] | 3 | 1 | 3 | Good |
| Withagen 2005 [121] | 4 | 1 | 1 | Fair |
| Zen 2021 [122] | 4 | 2 | 3 | Good |

**Table F. Association between hypertensive disorders of pregnancy and individual components of global developmental delay.**

| **Components of Global Developmental Delay** | **No. Studies** | **References** | **No. Participants** | **I^2^ (%)** | **Odds Ratio (95% CI)** |
| --- | --- | --- | --- | --- | --- |
| Behaviour problems | 6 | [23, 42, 59, 79, 89, 94] | 119,741 | 76.56 | 0.88 (0.60, 1.29) |
| Cognitive delay | 10 | [1, 13, 24, 28, 54, 79, 88, 89, 96, 99] | 51,325 | 73.13 | 1.30 (0.89, 1.89) |
| Communication delay | 5 | [23, 27, 44, 64, 70] | 776,265 | 76.81 | 1.18 (0.98, 1.41) |
| Fine motor delay | 7 | [25, 27, 42, 44, 54, 64, 70] | 934,534 | 63.27 | 1.17 (1.04, 1.32) |
| Gross motor delay | 7 | [25, 27, 42, 44, 54, 64, 70] | 934,536 | 74.77 | 1.23 (1.08, 1.40) |
| Hyperactivity/impulsivity^a^ | 4 | [42, 59, 71, 89] | 135,628 | 79.89 | 1.43 (0.88, 2.07) |
| Language delay | 5 | [1, 24, 25, 89, 121] | 6298 | 39.96 | 1.75 (1.35, 2.27) |
| Personal-social problems | 9 | [23, 25, 39, 42, 44, 59, 70, 71] | 281,709 | 76.09 | 1.21 (1.05, 1.41) |
| Problem solving delay | 4 | [27, 44, 64, 70] | 811,983 | 88.91 | 1.09 (0.90, 1.33) |

*^a^Not classified as attention-deficit/hyperactivity disorder.*

**Table G. Association between hypertensive disorders of pregnancy and educational achievement.**

| **Educational Achievement** | **No. Studies** | **References** | **No. Participants** | **I^2^ (%)** | **Odds Ratio (95% CI)** |
| --- | --- | --- | --- | --- | --- |
| Overall | 2 | [98, 103] | 475,452 | 0.00 | 1.36 (1.27, 1.45) |
| Mathematics | 2 | [8, 122] | 64,943 | 88.04 | 1.08 (0.85, 1.36) |
| Reading | 2 | [8, 122] | 64,943 | 0.01 | 1.14 (1.05, 1.23) |

**Table H. Association between hypertensive disorders of pregnancy and neurodevelopmental disability, stratified by age at assessment.**

| **Educational Achievement** | **No. Studies** | **References** | **No. Participants** | **I^2^ (%)** | **Odds Ratio (95% CI)** |
| --- | --- | --- | --- | --- | --- |
| **Autism Spectrum Disorder^a^** | | | | | |
| Infant (0 – 2 years) | 1 | – | – | – | – |
| Child (3 – 5 years) | 7 | [14, 21, 22, 30, 34, 61, 86] | 762,891 | 0.01 | 1.49 (1.39, 1.60) |
| Primary School (6 – 11 years) | 9 | [2, 4, 5, 18, 26, 35, 41, 68, 83] | 886,495 | 89.05 | 2.40 (1.45, 3.96) |
| Adolescent (12 – 17 years) | 0 | – | – | – | – |
| Adult (≥18 years) | 2 | [17, 60] | 1,085,201 | 61.80 | 1.82 (0.73, 4.54) |
| **Attention-Deficit/Hyperactivity Disorder^a^** | | | | | |
| Infant (0 – 2 years) | 0 | – | – | – | – |
| Child (3 – 5 years) | 2 | [20, 87] | 908,862 | 5.92 | 1.40 (1.12, 1.74) |
| Primary School (6 – 11 years) | 7 | [3, 11, 15, 26, 36, 41, 47] | 2,099,715 | 87.49 | 1.63 (1.28, 2.09) |
| Adolescent (12 – 17 years) | 1 | – | – | – | – |
| Adult (≥18 years) | 1 | – | – | – | – |
| **Cerebral Palsy^a^** | | | | | |
| Infant (0 – 2 years) | 8 | [1, 28, 48, 82, 100, 104, 105, 119] | 2461 | 69.33 | 0.96 (0.40, 2.31) |
| Child (3 – 5 years) | 4 | [78, 81, 84, 107] | 239,085 | 79.91 | 0.81 (0.47, 1.37) |
| Primary School (6 – 11 years) | 1 | – | – | – | – |
| Adolescent (12 – 17 years) | 0 | – | – | – | – |
| Adult (≥18 years) | 0 | – | – | – | – |
| **Intellectual Disability^a^** | | | | | |
| Infant (0 – 2 years) | 1 | – | – | – | – |
| Child (3 – 5 years) | 1 | – | – | – | – |
| Primary School (6 – 11 years) | 2 | [26, 112] | 974,742 | 99.13 | 3.77 (0.87, 16.29) |
| Adolescent (12 – 17 years) | 0 | – | – | – | – |
| Adult (≥18 years) | 1 | – | – | – | – |
| **Global Developmental Delay^a^** | | | | | |
| Infant (0 – 2 years) | 14 | [7, 10, 25, 27, 49, 51, 70, 75, 96, 100, 101, 104, 105, 114] | 93,410 | 99.21 | 2.07 (0.85, 5.09) |
| Child (3 – 5 years) | 4 | [61, 64, 78, 98] | 1,418,696 | 84.12 | 1.16 (0.98, 1.36) |
| Primary School (6 – 11 years) | 6 | [26, 31, 52, 62, 71, 115] | 921,770 | 50.23 | 1.65 (1.41, 1.95) |
| Adolescent (12 – 17 years) | 0 | – | – | – | – |
| Adult (≥18 years) | 0 | – | – | – | – |
| **Intelligence Quotient (IQ)^b^** | | | | | |
| Infant (0 – 2 years) | 0 | – | – | – | – |
| Child (3 – 5 years) | 2 | [67, 76] | 378 | 0.00 | -6.02 (-8.35, -3.68) |
| Primary School (6 – 11 years) | 5 | [31, 65, 77, 116, 120] | 4524 | 80.88 | -1.05 (-4.00, 1.90) |
| Adolescent (12 – 17 years) | 2 | [12, 97] | 33,673 | 0.00 | -1.42 (-1.82, -1.03) |
| Adult (≥18 years) | 2 | [17, 40] | 1,109,272 | 86.20 | -1.35 (-2.99, 0.30) |

*^a^ Effect size reported as odds ratio (95% confidence interval)
^b^ Effect size reported as mean difference (95% confidence interval)*

**Table I. Association between hypertensive disorders of pregnancy and neurodevelopmental disability, restricted to papers at low risk of bias.**

| **Outcome** | **No. Papers** | **References** | **No. Participants** | **I^2^ (%)** | **Odds Ratio (95% CI) or Mean Difference (95% CI)** |
| --- | --- | --- | --- | --- | --- |
| Autism Spectrum Disorder^a^ | 30 | [3, 5, 14, 17-19, 21, 26, 33, 35, 38, 41, 46, 53, 55-58, 63, 68, 69, 72, 80, 85, 92, 93, 102, 108, 111, 113] | 20,459,760 | 63.83% | 1.42 (1.36, 1.49) |
| Attention-Deficit/ Hyperactivity Disorder^a^ | 15 | [9, 11, 17, 20, 26, 32, 36, 41, 58, 73, 87, 91, 102, 103, 111] | 12,872,592 | 85.31% | 1.27 (1.19, 1.35) |
| Cerebral Palsy^a^ | 12 | [1, 26, 48, 74, 78, 81, 84, 85, 90, 100, 102, 107] | 2,556,187 | 96.16% | 1.41 (0.77, 2.57) |
| Intellectual Disability^a^ | 8 | [17, 26, 50, 58, 102, 109, 111, 112] | 10,304,992 | 99.07% | 1.87 (1.25, 2.79) |
| Developmental Delay^a^ | 22 | [25, 26, 31, 33, 37, 49, 52, 58, 59, 62, 64, 70, 71, 75, 78, 96, 98, 100, 101, 110, 114, 115] | 2,912,809 | 97.58% | 1.41 (1.18, 1.73) |
| Intelligence Quotient (IQ)^b^ | 9 | [12, 17, 31, 40, 65, 67, 76, 97, 116, 120] | 1,123,507 | 99.97% | -2.20 (-3.33, -1.07) |

*^a^ Effect size reported as odds ratio (95% confidence interval)
^b^ Effect size reported as mean difference (95% confidence interval)*


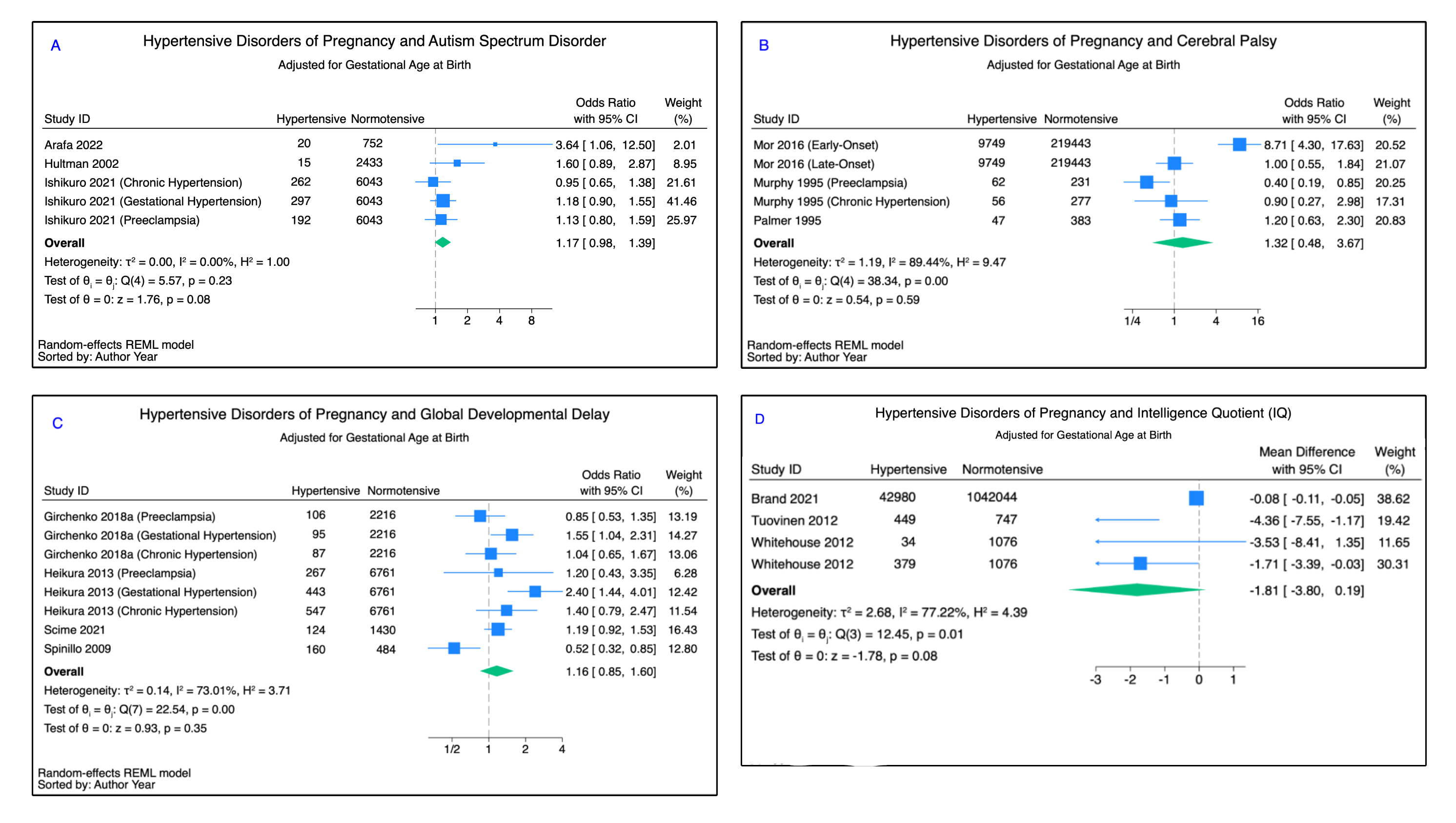


**Figure A. Association between hypertensive disorders of pregnancy and neurodevelopmental disabilities, among studies which adjusted for gestational age at birth. (A)** Autism spectrum disorder, reported as an adjusted odds ratio (95% CI). **(B)** Cerebral palsy, reported as an adjusted odds ratio (95% CI). **(C)** Global developmental delay, reported as an adjusted odds ratio (95% CI). **(D)** Intelligence quotient (IQ), reported as an adjusted mean difference (95% CI).


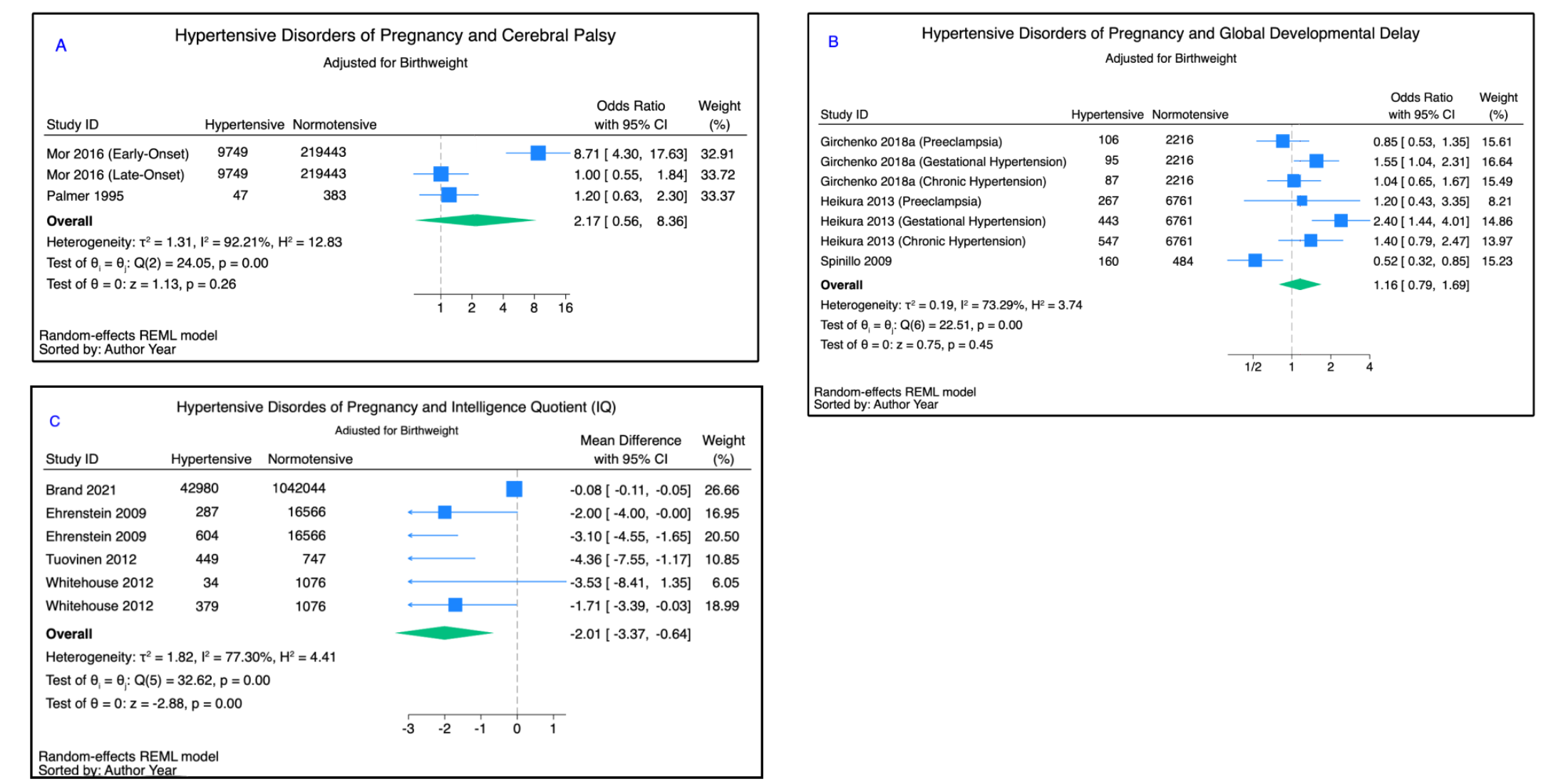


**Figure B. Association between hypertensive disorders of pregnancy and neurodevelopmental disabilities, among studies which adjusted for birthweight. (A)** Cerebral palsy, reported as an adjusted odds ratio (95% CI). **(B)** Global developmental delay, reported as an adjusted odds ratio (95% CI). **(C)** Intelligence quotient (IQ), reported as an adjusted mean difference (95% CI).


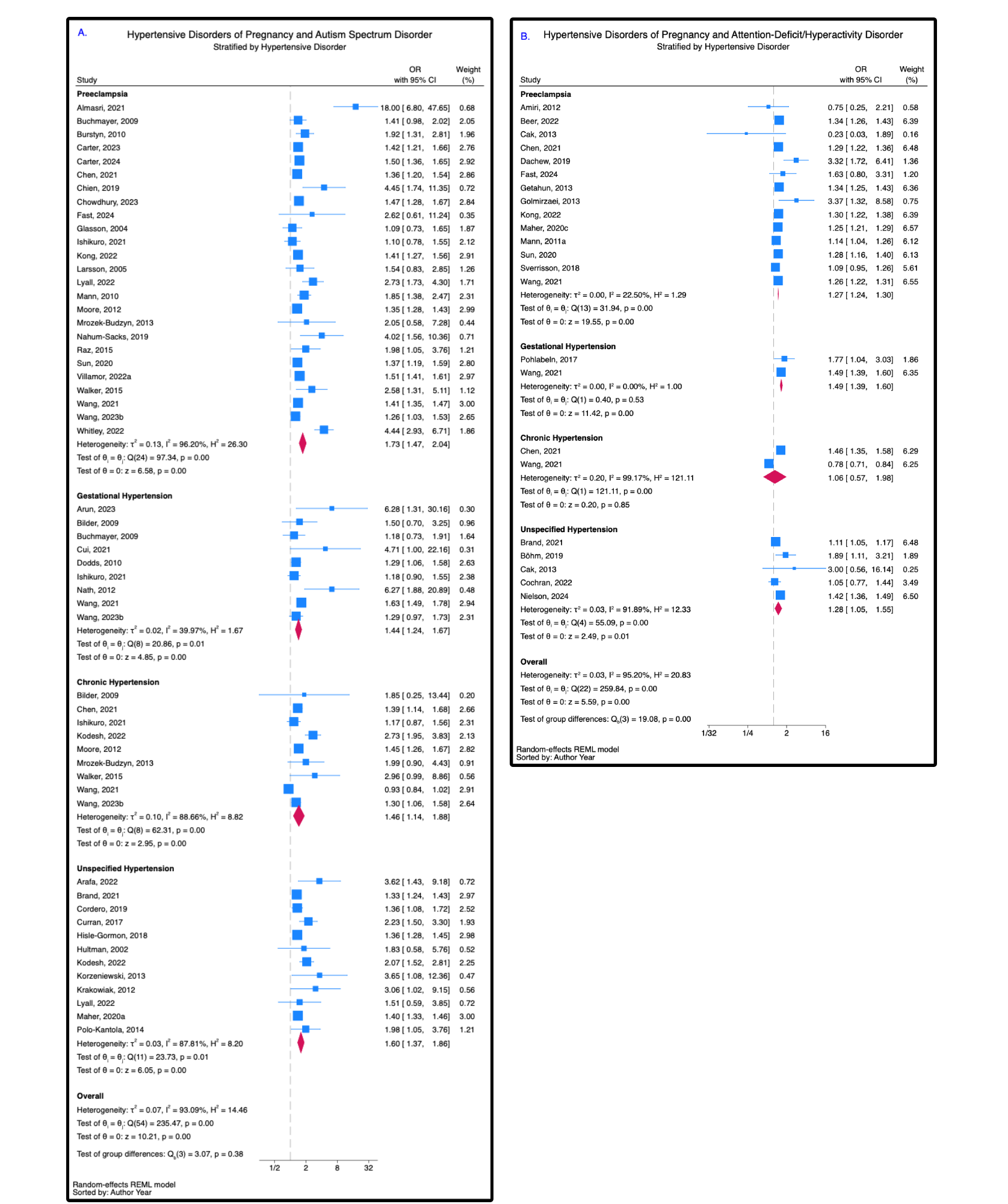


**Figure C. Association between hypertensive disorders of pregnancy and autism spectrum disorder and attention-deficit/hyperactivity disorder, stratified by type of hypertension. (A)** Autism spectrum disorder, reported as an unadjusted odds ratio (95% CI). **(B)** Attention-deficit/hyperactivity disorder, reported as an unadjusted odds ratio (95% CI).


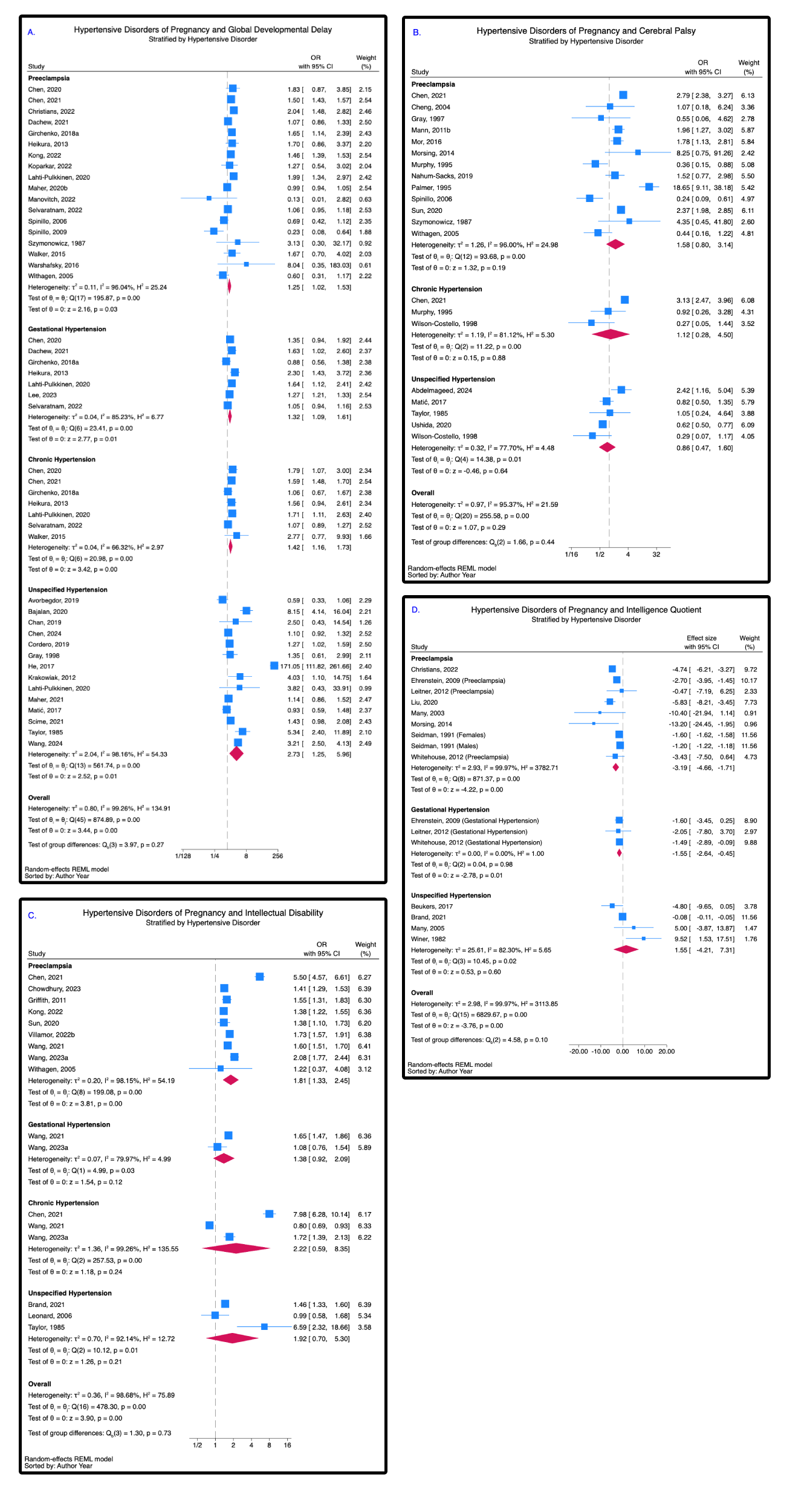


**Figure D. Association between hypertensive disorders of pregnancy and other neurodevelopmental disabilities, stratified by type of hypertension. (A)** Global developmental delay, reported as an unadjusted odds ratio (95% CI). **(B)** Cerebral palsy, reported as an unadjusted odds ratio (95% CI). **(C)** Intellectual disability, reported as an unadjusted odds ratio (95% CI). **(D)** Intelligence quotient, reported as an unadjusted mean difference (95% CI).


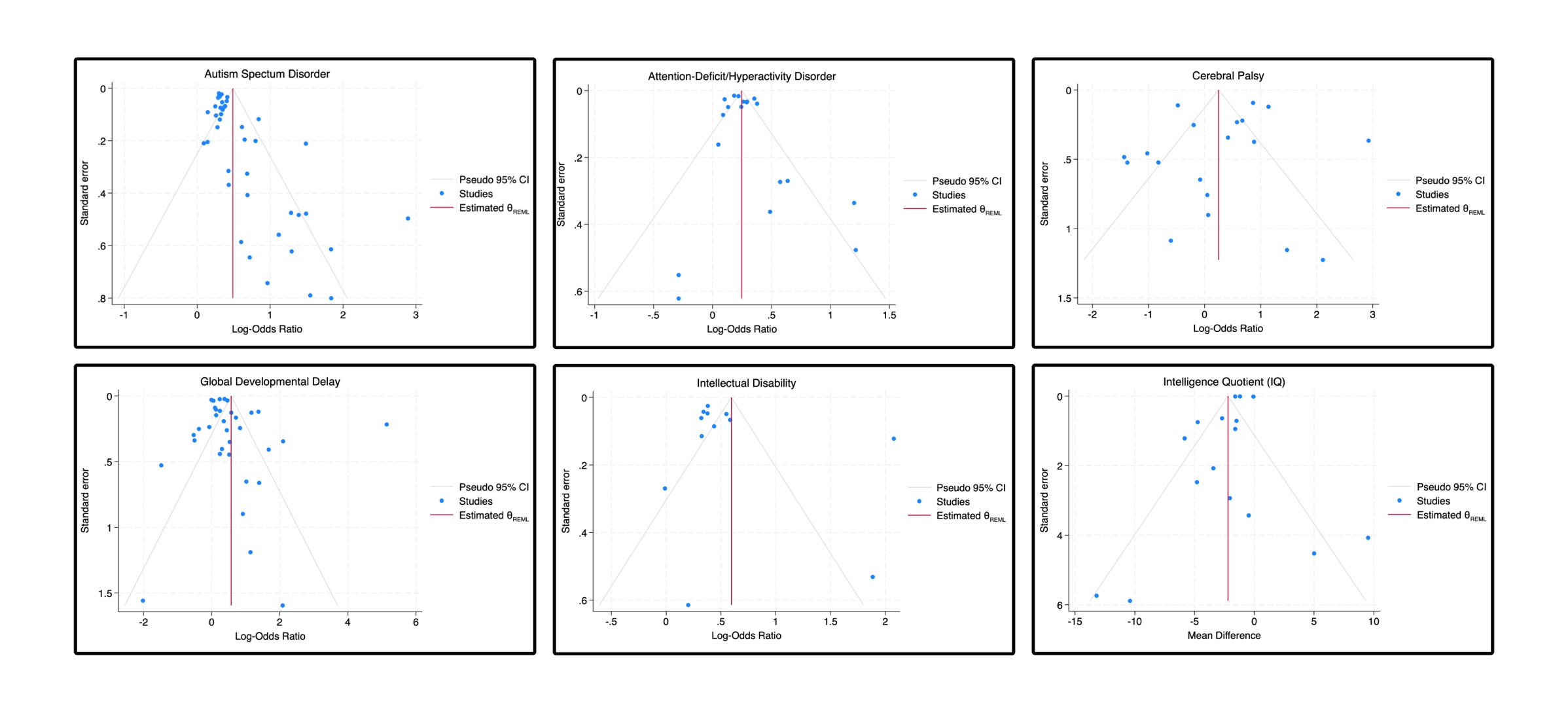


**Figure E. Funnel plots detailing publication bias in meta-analyses for neurodevelopmental disabilities.**

**References**

1. Abdelmageed WA, Lapointe A, Brown R, Gorgos A, Luu TM, Beltempo M, et al. Association between maternal hypertension and infant neurodevelopment in extremely preterm infants. JOURNAL OF PERINATOLOGY. 2024;44(4):539-47. doi: 10.1038/s41372-024-01886-7. PubMed PMID: WOS:001153705100003.

2. Almasri J, Barazi A, King KS, Walther-Antonio MRS, Wang Z, Murad MH, et al. Peripartum Antibiotics Exposure and the Risk of Autoimmune and Autism Disorders in the Offspring. Avicenna journal of medicine. 2021;11(3):118-25. doi: <https://dx.doi.org/10.1055/s-0041-1732485>.

3. Amiri S, Malek A, Sadegfard M, Abdi S. Pregnancy-related maternal risk factors of attention-deficit hyperactivity disorder: a case-control study. ISRN Pediatr. 2012;2012:458064. doi: 10.5402/2012/458064. PubMed PMID: 22720167.

4. Arafa A, Mahmoud O, Salah H, Abdelmonem AA, Senosy S. Maternal and neonatal risk factors for autism spectrum disorder: A case-control study from Egypt. PloS one. 2022;17(6):e0269803. doi: <https://dx.doi.org/10.1371/journal.pone.0269803>.

5. Arun P, Azad C, Kaur G, Sharma P. A Community-Based Study of Antenatal and Neonatal Risk Factors in Autism Spectrum Disorder. The primary care companion for CNS disorders. 2023;25(2). doi: <https://dx.doi.org/10.4088/PCC.22m03339>.

6. Carter S, Lin JC, Chow T, Martinez MP, Qiu C, Feldman RK, et al. Preeclampsia Onset, Days to Delivery, and Autism Spectrum Disorders in Offspring: Clinical Birth Cohort Study. JMIR Public Health Surveill. 2024;10:e47396. doi: 10.2196/47396.

7. Avorgbedor F, Silva S, Merwin E, Blumenthal JA, Holditch-Davis D. Health, Physical Growth, and Neurodevelopmental Outcomes in Preterm Infants of Women With Hypertensive Disorders of Pregnancy. JOGNN: Journal of Obstetric, Gynecologic & Neonatal Nursing. 2019;48(1):69-77. doi: 10.1016/j.jogn.2018.10.003.

8. Ayala NK, Schlichting LE, Kempner M, Clark MA, Vivier PM, Viner-Brown SI, et al. Association between maternal hypertensive disorders, fetal growth and childhood learning outcomes. Pregnancy hypertension. 2021;25(101552483):249-54. doi: <https://dx.doi.org/10.1016/j.preghy.2021.07.242>.

9. Maher GM, Dalman C, O'Keeffe GW, Kearney PM, McCarthy FP, Kenny LC, et al. Association between preeclampsia and attention-deficit hyperactivity disorder: a population-based and sibling-matched cohort study. Acta psychiatrica Scandinavica. 2020;142(4):275-83. doi: <https://dx.doi.org/10.1111/acps.13162>.

10. Bajalan Z, Alimoradi Z. Risk factors of developmental delay among infants aged 6-18 months. EARLY CHILD DEVELOPMENT AND CARE. 2020;190(11):1691-9. doi: 10.1080/03004430.2018.1547714. PubMed PMID: WOS:000576223900003.

11. Beer RJ, Cnattingius S, Susser ES, Villamor E. Associations of preterm birth, small-for-gestational age, preeclampsia and placental abruption with attention-deficit/hyperactivity disorder in the offspring: Nationwide cohort and sibling-controlled studies. Acta paediatrica (Oslo, Norway : 1992). 2022;111(8):1546-55. doi: <https://dx.doi.org/10.1111/apa.16375>.

12. Beukers F, Aarnoudse-Moens CSH, van Weissenbruch MM, Ganzevoort W, van Goudoever JB, van Wassenaer-Leemhuis AG. Fetal Growth Restriction with Brain Sparing: Neurocognitive and Behavioral Outcomes at 12 Years of Age. The Journal of pediatrics. 2017;188(jlz, 0375410):103-9.e2. doi: <https://dx.doi.org/10.1016/j.jpeds.2017.06.003>.

13. Bharadwaj SK, Vishnu Bhat B, Vickneswaran V, Adhisivam B, Bobby Z, Habeebullah S. Oxidative Stress, Antioxidant Status and Neurodevelopmental Outcome in Neonates Born to Pre-eclamptic Mothers. Indian journal of pediatrics. 2018;85(5):351-7. doi: <https://dx.doi.org/10.1007/s12098-017-2560-5>.

14. Bilder D, Pinborough-Zimmerman J, Miller J, McMahon W. Prenatal, perinatal, and neonatal factors associated with autism spectrum disorders. Pediatrics. 2009;123(5):1293-300. doi: 10.1542/peds.2008-0927. PubMed PMID: 19403494.

15. Böhm S, Curran EA, Kenny LC, O'Keeffe GW, Murray D, Khashan AS. The Effect of Hypertensive Disorders of Pregnancy on the Risk of ADHD in the Offspring. J Atten Disord. 2019;23(7):692-701. Epub 20170204. doi: 10.1177/1087054717690230. PubMed PMID: 28162026.

16. Bolk J, Kallen K, Farooqi A, Hafstrom M, Fellman V, Aden U, et al. Perinatal risk factors for developmental coordination disorder in children born extremely preterm. Acta Paediatrica. 2023;112(4):675-85. doi: <https://dx.doi.org/10.1111/apa.16651>.

17. Brand JS, Lawlor DA, Larsson H, Montgomery S. Association Between Hypertensive Disorders of Pregnancy and Neurodevelopmental Outcomes Among Offspring. JAMA pediatrics. 2021;175(6):577-85. doi: <https://dx.doi.org/10.1001/jamapediatrics.2020.6856>.

18. Buchmayer S, Johansson S, Johansson A, Hultman CM, Sparen P, Cnattingius S. Can association between preterm birth and autism be explained by maternal or neonatal morbidity? Pediatrics. 2009;124(5):e817-25. doi: <https://dx.doi.org/10.1542/peds.2008-3582>.

19. Burstyn I, Sithole F, Zwaigenbaum L. Autism spectrum disorders, maternal characteristics and obstetric complications among singletons born in Alberta, Canada. Chronic diseases in Canada. 2010;30(4):125-34. PubMed PMID: 20946713.

20. Çak HT, Gökler B. Attention deficit hyperactivity disorder and associated perinatal risk factors in preterm children. Turkish Archives of Pediatrics. 2013:315-22.

21. Carter SA, Lin JC, Chow T, Yu X, Rahman MM, Martinez MP, et al. Maternal obesity, diabetes, preeclampsia, and asthma during pregnancy and likelihood of autism spectrum disorder with gastrointestinal disturbances in offspring. Autism : the international journal of research and practice. 2023;27(4):916-26. doi: <https://dx.doi.org/10.1177/13623613221118430>.

22. Carter S, Lin JC, Chow T, Martinez MP, Qiu C, Feldman RK, et al. Preeclampsia Onset, Days to Delivery, and Autism Spectrum Disorders in Offspring: Clinical Birth Cohort Study. JMIR public health and surveillance. 2024;10(101669345):e47396. doi: <https://dx.doi.org/10.2196/47396>.

23. Chan SE, Pudwell J, Smith GN. Effects of Preeclampsia on Maternal and Pediatric Health at 11 Years Postpartum. American journal of perinatology. 2019;36(8):806-11. doi: <https://dx.doi.org/10.1055/s-0038-1675374>.

24. Chang H-Y, Chen C-P, Sun F-J, Chen C-Y. Influence of pre-eclampsia on 2-year neurodevelopmental outcome of very-low-birth-weight infants. International journal of gynaecology and obstetrics: the official organ of the International Federation of Gynaecology and Obstetrics. 2023;161(3):979-88. doi: <https://dx.doi.org/10.1002/ijgo.14614>.

25. Chen Z, Li R, Liu H, Duan J, Yao C, Yang R, et al. Impact of maternal hypertensive disorders on offspring's neurodevelopment: a longitudinal prospective cohort study in China. Pediatric research. 2020;88(4):668-75. doi: <https://dx.doi.org/10.1038/s41390-020-0794-9>.

26. Chen K-R, Yu T, Kang L, Lien Y-J, Kuo P-L. Childhood neurodevelopmental disorders and maternal hypertensive disorder of pregnancy. Developmental medicine and child neurology. 2021;63(9):1107-13. doi: <https://dx.doi.org/10.1111/dmcn.14893>.

27. Chen G, Ishikuro M, Ohseto H, Murakami K, Noda A, Shinoda G, et al. Hypertensive disorders of pregnancy, neonatal outcomes and offspring developmental delay in Japan: The Tohoku Medical Megabank Project Birth and Three-Generation Cohort Study. Acta obstetricia et gynecologica Scandinavica. 2024;103(6):1192-200. doi: <https://dx.doi.org/10.1111/aogs.14820>.

28. Cheng S-W, Chou H-C, Tsou K-I, Fang L-J, Tsao P-N. Delivery before 32 weeks of gestation for maternal pre-eclampsia: neonatal outcome and 2-year developmental outcome. Early human development. 2004;76(1):39-46.

29. Chien Y-L, Chou M-C, Chou W-J, Wu Y-Y, Tsai W-C, Chiu Y-N, et al. Prenatal and perinatal risk factors and the clinical implications on autism spectrum disorder. Autism : the international journal of research and practice. 2019;23(3):783-91. doi: <https://dx.doi.org/10.1177/1362361318772813>.

30. Chowdhury MAK, Hardin JW, Love BL, Merchant AT, McDermott S. Relationship of nonsteroidal anti-inflammatory drug use during pregnancy with autism spectrum disorder and intellectual disability among offspring. Journal of Women's Health. 2023;32(3):356-65. doi: <https://dx.doi.org/10.1089/jwh.2022.0113>.

31. Christians JK, Chow NA. Are there sex differences in fetal growth strategies and in the long-term effects of pregnancy complications on cognitive functioning? JOURNAL OF DEVELOPMENTAL ORIGINS OF HEALTH AND DISEASE. 2022;13(6):766-78. doi: 10.1017/S2040174422000204. PubMed PMID: WOS:000783015600001.

32. Cochran DM, Jensen ET, Frazier JA, Jalnapurkar I, Kim S, Roell KR, et al. Association of prenatal modifiable risk factors with attention-deficit hyperactivity disorder outcomes at age 10 and 15 in an extremely low gestational age cohort. Front Hum Neurosci. 2022;16:911098. Epub 20221020. doi: 10.3389/fnhum.2022.911098. PubMed PMID: 36337853; PubMed Central PMCID: PMCPMC9630552.

33. Cordero C, Windham GC, Schieve LA, Fallin MD, Croen LA, Siega-Riz AM, et al. Maternal diabetes and hypertensive disorders in association with autism spectrum disorder. Autism research : official journal of the International Society for Autism Research. 2019;12(6):967-75. doi: <https://dx.doi.org/10.1002/aur.2105>.

34. Cui L, Du W, Xu N, Dong J, Xia B, Ma J, et al. Impact of MicroRNAs in interaction with environmental factors on autism spectrum disorder: An exploratory pilot study. Frontiers in Psychiatry. 2021;12(Beversdorf, DQ, Stevens, HE, & Jones, KL. Prenatal Stress, Maternal immune dysregulation, and their association with autism spectrum disorders. Curr Psychiatry Rep. (2018) 20:76 <https://pubmed.ncbi.nlm.nih.gov/30094645> <https://dx.doi.org/10.1007/s11920-01>). doi: <https://dx.doi.org/10.3389/fpsyt.2021.715481>.

35. Curran EA, O'Keeffe GW, Looney AM, Moloney G, Hegarty SV, Murray DM, et al. Exposure to Hypertensive Disorders of Pregnancy Increases the Risk of Autism Spectrum Disorder in Affected Offspring. Mol Neurobiol. 2018;55(7):5557-64. doi: 10.1007/s12035-017-0794-x. PubMed PMID: 28975539.

36. Dachew BA, Scott JG, Mamun A, Alati R. Pre-eclampsia and the risk of attention-deficit/hyperactivity disorder in offspring: Findings from the ALSPAC birth cohort study. Psychiatry Res. 2019;272:392-7. Epub 20181225. doi: 10.1016/j.psychres.2018.12.123. PubMed PMID: 30605798.

37. Dachew BA, Scott JG, Mamun A, Fetene DM, Alati R. Maternal hypertensive disorders during pregnancy and the trajectories of offspring emotional and behavioral problems: the ALSPAC birth cohort study. Annals of epidemiology. 2021;53(9100013, bx8):63-8.e1. doi: <https://dx.doi.org/10.1016/j.annepidem.2020.08.015>.

38. Dodds L, Fell DB, Shea S, Armson BA, Allen AC, Bryson S. The Role of Prenatal, Obstetric and Neonatal Factors in the Development of Autism. Journal of Autism and Developmental Disorders. 2011;41(7):891-902. doi: 10.1007/s10803-010-1114-8.

39. Duko B, Gebremedhin AT, Tessema GA, Dunne J, Alati R, Pereira G. The effects of pre-eclampsia on social and emotional developmental vulnerability in children at age five in Western Australia: A population data linkage study. Journal of affective disorders. 2024;352(h3v, 7906073):349-56. doi: <https://dx.doi.org/10.1016/j.jad.2024.02.042>.

40. Ehrenstein V, Rothman KJ, Pedersen L, Hatch EE, Sorensen HT. Pregnancy-associated hypertensive disorders and adult cognitive function among Danish conscripts. American journal of epidemiology. 2009;170(8):1025-31. doi: <https://dx.doi.org/10.1093/aje/kwp223>.

41. Fast K, Wentz E, Roswall J, Strandberg M, Bergman S, Dahlgren J. Prevalence of attention-deficit/hyperactivity disorder and autism in 12-year-old children: A population-based cohort. Developmental medicine and child neurology. 2024;66(4):493-500. doi: <https://dx.doi.org/10.1111/dmcn.15757>.

42. Fitton CA, Fleming M, Aucott L, Pell JP, Mackay DF, McLay JS. Congenital defects and early childhood outcomes following in-utero exposure to antihypertensive medication. Journal of hypertension. 2021;39(3):581-8. doi: <https://dx.doi.org/10.1097/HJH.0000000000002670>.

43. Getahun D, Rhoads GG, Demissie K, Lu S-E, Quinn VP, Fassett MJ, et al. In utero exposure to ischemic-hypoxic conditions and attention-deficit/hyperactivity disorder. Pediatrics. 2013;131(1):e53-61. doi: <https://dx.doi.org/10.1542/peds.2012-1298>.

44. Girchenko P, Tuovinen S, Lahti-Pulkkinen M, Lahti J, Savolainen K, Heinonen K, et al. Maternal early pregnancy obesity and related pregnancy and pre-pregnancy disorders: associations with child developmental milestones in the prospective PREDO Study. International journal of obesity (2005). 2018;42(5):995-1007. doi: <https://dx.doi.org/10.1038/s41366-018-0061-x>.

45. Girchenko P, Lahti-Pulkkinen M, Lahti J, Pesonen AK, Hämäläinen E, Villa PM, et al. Neonatal regulatory behavior problems are predicted by maternal early pregnancy overweight and obesity: findings from the prospective PREDO Study. Pediatr Res. 2018;84(6):875-81. Epub 20181010. doi: 10.1038/s41390-018-0199-1. PubMed PMID: 30305694.

46. Glasson EJ, Bower C, Petterson B, de Klerk N, Chaney G, Hallmayer JF. Perinatal Factors and the Development of Autism: A Population Study. Archives of General Psychiatry. 2004;61(6):618-27. doi: 10.1001/archpsyc.61.6.618.

47. Golmirzaei J, Namazi S, Amiri S, Zare S, Rastikerdar N, Hesam AA, et al. Evaluation of attention-deficit hyperactivity disorder risk factors. Int J Pediatr. 2013;2013:953103. doi: 10.1155/2013/953103. PubMed PMID: 24319465.

48. Gray PH, Hurley TM, Rogers YM, O'Callaghan MJ, Tudehope DI, Burns YR, et al. Survival and neonatal and neurodevelopmental outcome of 24-29 week gestation infants according to primary cause of preterm delivery. The Australian & New Zealand journal of obstetrics & gynaecology. 1997;37(2):161-8.

49. Gray PH, O'Callaghan MJ, Mohay HA, Burns YR, King JF. Maternal hypertension and neurodevelopmental outcome in very preterm infants. Archives of disease in childhood Fetal and neonatal edition. 1998;79(2):F88-93.

50. Griffith MI, Mann JR, McDermott S. The risk of intellectual disability in children born to mothers with preeclampsia or eclampsia with partial mediation by low birth weight. Hypertension in pregnancy. 2011;30(1):108-15. doi: <https://dx.doi.org/10.3109/10641955.2010.507837>.

51. He F, Li QP, Li NP, Yao L, Ma XW, Feng ZC. Analysis of high-risk factors and effect of early intervention on preterm infant neurodevelopment. INTERNATIONAL JOURNAL OF CLINICAL AND EXPERIMENTAL MEDICINE. 2017;10(3):5372-80. PubMed PMID: WOS:000400552900133.

52. Heikura U, Hartikainen A-L, Nordstrom T, Pouta A, Taanila A, Jarvelin M-R. Maternal hypertensive disorders during pregnancy and mild cognitive limitations in the offspring. Paediatric and perinatal epidemiology. 2013;27(2):188-98. doi: <https://dx.doi.org/10.1111/ppe.12028>.

53. Hisle-Gorman E, Susi A, Stokes T, Gorman G, Erdie-Lalena C, Nylund CM. Prenatal, perinatal, and neonatal risk factors of autism spectrum disorder. Pediatric research. 2018;84(2):190-8. doi: <https://dx.doi.org/10.1038/pr.2018.23>.

54. Huang B, Wang Y, Jiang Y, Lv H, Jiang T, Qiu Y, et al. Association of maternal hypertensive disorders in pregnancy with infant neurodevelopment. Journal of biomedical research. 2023;37(6):479-91. doi: <https://dx.doi.org/10.7555/JBR.37.20230074>.

55. Hultman CM, Sparén P, Cnattingius S. Perinatal risk factors for infantile autism. Epidemiology. 2002;13(4):417-23. doi: 10.1097/00001648-200207000-00009. PubMed PMID: 12094096.

56. Ishikuro M, Murakami K, Yokozeki F, Onuma T, Noda A, Ueno F, et al. Hypertension in pregnancy as a possible factor for child autistic behavior at two years old. Pregnancy hypertension. 2021;25(101552483):88-90. doi: <https://dx.doi.org/10.1016/j.preghy.2021.05.020>.

57. Kodesh A, Levine SZ, Khachadourian V, Rahman R, Schlessinger A, O'Reilly PF, et al. Maternal health around pregnancy and autism risk: A diagnosis-wide, population-based study. Psychological Medicine. 2022;52(16):4076-84. doi: <https://dx.doi.org/10.1017/S0033291721001021>.

58. Kong L, Chen X, Liang Y, Forsell Y, Gissler M, Lavebratt C. Association of Preeclampsia and Perinatal Complications With Offspring Neurodevelopmental and Psychiatric Disorders. JAMA network open. 2022;5(1):e2145719. doi: <https://dx.doi.org/10.1001/jamanetworkopen.2021.45719>.

59. Koparkar S, Srivastava L, Randhir K, Dangat K, Pisal H, Kadam V, et al. Cognitive function and behavioral problems in children born to mothers with preeclampsia: an Indian study. Child neuropsychology : a journal on normal and abnormal development in childhood and adolescence. 2022;28(3):337-54. doi: <https://dx.doi.org/10.1080/09297049.2021.1978418>.

60. Korzeniewski SJ, Pinto-Martin JA, Whitaker AH, Feldman JF, Lorenz JM, Levy SE, et al. Association between transient hypothyroxinaemia of prematurity and adult autism spectrum disorder in a low-birthweight cohort: an exploratory study. Paediatric and perinatal epidemiology. 2013;27(2):182-7. doi: <https://dx.doi.org/10.1111/ppe.12034>.

61. Krakowiak P, Walker CK, Bremer AA, Baker AS, Ozonoff S, Hansen RL, et al. Maternal metabolic conditions and risk for autism and other neurodevelopmental disorders. Pediatrics. 2012;129(5):e1121-8. Epub 20120409. doi: 10.1542/peds.2011-2583. PubMed PMID: 22492772; PubMed Central PMCID: PMCPMC3340592.

62. Lahti-Pulkkinen M, Girchenko P, Tuovinen S, Sammallahti S, Reynolds RM, Lahti J, et al. Maternal Hypertensive Pregnancy Disorders and Mental Disorders in Children. Hypertension (Dallas, Tex : 1979). 2020;75(6):1429-38. doi: <https://dx.doi.org/10.1161/HYPERTENSIONAHA.119.14140>.

63. Larsson HJ, Eaton WW, Madsen KM, Vestergaard M, Olesen AV, Agerbo E, et al. Risk factors for autism: perinatal factors, parental psychiatric history, and socioeconomic status. Am J Epidemiol. 2005;161(10):916-25; discussion 26. doi: 10.1093/aje/kwi123. PubMed PMID: 15870155.

64. Lee S, Han Y, Lim MK, Lee HJ. Impact of moderate-to-late preterm birth on neurodevelopmental outcomes in young children: Results from retrospective longitudinal follow-up with nationally representative data. PloS one. 2023;18(11):e0294435. doi: <https://dx.doi.org/10.1371/journal.pone.0294435>.

65. Leitner Y, Harel S, Geva R, Eshel R, Yaffo A, Many A. The neurocognitive outcome of IUGR children born to mothers with and without preeclampsia. The journal of maternal-fetal & neonatal medicine : the official journal of the European Association of Perinatal Medicine, the Federation of Asia and Oceania Perinatal Societies, the International Society of Perinatal Obstetricians. 2012;25(11):2206-8. doi: <https://dx.doi.org/10.3109/14767058.2012.684164>.

66. Leonard H, de Klerk N, Bourke J, Bower C. Maternal Health in Pregnancy and Intellectual Disability in the Offspring: A Population-Based Study. Annals of Epidemiology. 2006;16(6):448-54. doi: <https://doi.org/10.1016/j.annepidem.2005.05.002>.

67. Liu L, Lin Z, Zheng B, Wang L, Zou J, Wu S, et al. Reduced Intellectual Ability in Offspring Born from Preeclamptic Mothers: A Prospective Cohort Study. Risk management and healthcare policy. 2020;13(101566264):2037-46. doi: <https://dx.doi.org/10.2147/RMHP.S277521>.

68. Lyall K, Ning X, Aschner JL, Avalos LA, Bennett DH, Bilder DA, et al. Cardiometabolic Pregnancy Complications in Association With Autism-Related Traits as Measured by the Social Responsiveness Scale in ECHO. American journal of epidemiology. 2022;191(8):1407-19. doi: <https://dx.doi.org/10.1093/aje/kwac061>.

69. Maher GM, O'Keeffe GW, Dalman C, Kearney PM, McCarthy FP, Kenny LC, et al. Association between preeclampsia and autism spectrum disorder: a population-based study. Journal of child psychology and psychiatry, and allied disciplines. 2020;61(2):131-9. doi: <https://dx.doi.org/10.1111/jcpp.13127>.

70. Maher GM, O'Keeffe GW, O'Keeffe LM, Matvienko-Sikar K, Dalman C, Kearney PM, et al. The Association Between Preeclampsia and Childhood Development and Behavioural Outcomes. Maternal and child health journal. 2020;24(6):727-38. doi: <https://dx.doi.org/10.1007/s10995-020-02921-7>.

71. Maher GM, McCarthy FP, Khashan AS. Hypertensive Disorders of Pregnancy and Behavioural Outcomes in the Offspring: Findings from the Millennium Cohort Study. Journal of affective disorders. 2021;287(h3v, 7906073):222-8. doi: <https://dx.doi.org/10.1016/j.jad.2021.03.040>.

72. Mann JR, McDermott S, Bao H, Hardin J, Gregg A. Pre-eclampsia, birth weight, and autism spectrum disorders. Journal of autism and developmental disorders. 2010;40(5):548-54. doi: <https://dx.doi.org/10.1007/s10803-009-0903-4>.

73. Mann JR, McDermott S. Are maternal genitourinary infection and pre-eclampsia associated with ADHD in school-aged children? Journal of attention disorders. 2011;15(8):667-73. doi: <https://dx.doi.org/10.1177/1087054710370566>.

74. Mann JR, McDermott S, Griffith MI, Hardin J, Gregg A. Uncovering the complex relationship between pre-eclampsia, preterm birth and cerebral palsy. Paediatric and perinatal epidemiology. 2011;25(2):100-10. doi: <https://dx.doi.org/10.1111/j.1365-3016.2010.01157.x>.

75. Manovitch Z, Morag I, Simchen MJ. Neurodevelopmental outcomes of preterm infants born to preeclamptic mothers - A case-control study. EUROPEAN JOURNAL OF OBSTETRICS & GYNECOLOGY AND REPRODUCTIVE BIOLOGY. 2022;270:6-10. doi: 10.1016/j.ejogrb.2021.12.036. PubMed PMID: WOS:000774443600002.

76. Many A, Fattal A, Leitner Y, Kupferminc MJ, Harel S, Jaffa A. Neurodevelopmental and cognitive assessment of children born growth restricted to mothers with and without preeclampsia. Hypertension in pregnancy. 2003;22(1):25-9.

77. Many A, Fattal-Valevski A, Leitner Y. Neurodevelopmental and cognitive assessment of 6-year-old children born growth restricted. International journal of gynaecology and obstetrics: the official organ of the International Federation of Gynaecology and Obstetrics. 2005;89(1):55-6.

78. Matić M, Inati V, Abdel ‐ Latif ME, Kent AL. Maternal hypertensive disorders are associated with increased use of respiratory support but not chronic lung disease or poorer neurodevelopmental outcomes in preterm neonates at <29 weeks of gestation. Journal of Paediatrics & Child Health. 2017;53(4):391-8. doi: 10.1111/jpc.13430.

79. McCowan LME, Pryor J, Harding JE. Perinatal predictors of neurodevelopmental outcome in small-for-gestational-age children at 18 months of age. American journal of obstetrics and gynecology. 2002;186(5):1069-75.

80. Moore GS, Kneitel AW, Walker CK, Gilbert WM, Xing G. Autism risk in small- and large-for-gestational-age infants. Am J Obstet Gynecol. 2012;206(4):314.e1-9. doi: 10.1016/j.ajog.2012.01.044. PubMed PMID: 22464070.

81. Mor O, Stavsky M, Yitshak-Sade M, Mastrolia SA, Beer-Weisel R, Rafaeli-Yehudai T, et al. Early onset preeclampsia and cerebral palsy: a double hit model? American journal of obstetrics and gynecology. 2016;214(1):105.e1-9. doi: <https://dx.doi.org/10.1016/j.ajog.2015.08.020>.

82. Morsing E, Marsal K. Pre-eclampsia- an additional risk factor for cognitive impairment at school age after intrauterine growth restriction and very preterm birth. Early human development. 2014;90(2):99-101. doi: <https://dx.doi.org/10.1016/j.earlhumdev.2013.12.002>.

83. Mrozek-Budzyn D, Majewska R, Kieltyka A. Prenatal, perinatal and neonatal risk factors for autism - study in Poland. Central European Journal of Medicine. 2013;8(4):424-30. doi: 10.2478/s11536-013-0174-5.

84. Murphy DJ, Sellers S, MacKenzie IZ, Yudkin PL, Johnson AM. Case-control study of antenatal and intrapartum risk factors for cerebral palsy in very preterm singleton babies. Lancet. 1995;346(8988):1449-54. doi: 10.1016/s0140-6736(95)92471-x.

85. Nahum Sacks K, Friger M, Shoham-Vardi I, Sergienko R, Spiegel E, Landau D, et al. Long-term neuropsychiatric morbidity in children exposed prenatally to preeclampsia. Early human development. 2019;130(edh, 7708381):96-100. doi: <https://dx.doi.org/10.1016/j.earlhumdev.2019.01.016>.

86. Nath S, Roy R, Mukherjee S. Perinatal complications associated with autism--a case control study in a neurodevelopment and early intervention clinic. Journal of the Indian Medical Association. 2012;110(8):526-9.

87. Nielsen TC, Nassar N, Shand AW, Jones HF, Han VX, Patel S, et al. Association between cumulative maternal exposures related to inflammation and child attention-deficit/hyperactivity disorder: A cohort study. PAEDIATRIC AND PERINATAL EPIDEMIOLOGY. 2024;38(3):241-50. doi: 10.1111/ppe.13022. PubMed PMID: WOS:001108723800001.

88. Noda M, Yoshida S, Mishina H, Matsubayashi K, Kawakami K. Association between maternal hypertensive disorders of pregnancy and child neurodevelopment at 3 years of age: a retrospective cohort study. Journal of developmental origins of health and disease. 2021;12(3):428-35. doi: <https://dx.doi.org/10.1017/S2040174420000586>.

89. Palatnik A, Mele L, Casey BM, Varner MW, Sorokin Y, Reddy UM, et al. Association between Hypertensive Disorders of Pregnancy and Long-Term Neurodevelopmental Outcomes in the Offspring. American journal of perinatology. 2022;39(9):921-9. doi: <https://dx.doi.org/10.1055/a-1692-0659>.

90. Palmer L, Blair E, Petterson B, Burton P. ANTENATAL ANTECEDENTS OF MODERATE AND SEVERE CEREBRAL-PALSY. PAEDIATRIC AND PERINATAL EPIDEMIOLOGY. 1995;9(2):171-84. doi: 10.1111/j.1365-3016.1995.tb00132.x. PubMed PMID: WOS:A1995QR95100007.

91. Pohlabeln H, Rach S, De Henauw S, Eiben G, Gwozdz W, Hadjigeorgiou C, et al. Further evidence for the role of pregnancy-induced hypertension and other early life influences in the development of ADHD: results from the IDEFICS study. European child & adolescent psychiatry. 2017;26(8):957-67. doi: <https://dx.doi.org/10.1007/s00787-017-0966-2>.

92. Polo-Kantola P, Lampi KM, Hinkka-Yli-Salomaki S, Gissler M, Brown AS, Sourander A. Obstetric risk factors and autism spectrum disorders in Finland. The Journal of pediatrics. 2014;164(2):358-65. doi: <https://dx.doi.org/10.1016/j.jpeds.2013.09.044>.

93. Raz R, Roberts AL, Lyall K, Hart JE, Just AC, Laden F, et al. Autism spectrum disorder and particulate matter air pollution before, during, and after pregnancy: a nested case-control analysis within the Nurses' Health Study II Cohort. Environ Health Perspect. 2015;123(3):264-70. doi: 10.1289/ehp.1408133. PubMed PMID: 25522338.

94. Robinson M, Mattes E, Oddy WH, de Klerk NH, Li J, McLean NJ, et al. Hypertensive diseases of pregnancy and the development of behavioral problems in childhood and adolescence: the Western Australian Pregnancy Cohort Study. The Journal of pediatrics. 2009;154(2):218-24. doi: <https://dx.doi.org/10.1016/j.jpeds.2008.07.061>.

95. Sabino AT, Souza E, Goulart AL, Lima AM, Sass N. High Blood Pressure during Pregnancy is not a Protective Factor for Preterm Infants with Very Low Birth Weight. A Case-Control Study. Rev Bras Ginecol Obstet. 2017;39(4):155-61. Epub 20170413. doi: 10.1055/s-0037-1601883. PubMed PMID: 28407656; PubMed Central PMCID: PMCPMC10309464.

96. Scime NV, Hetherington E, Tomfohr-Madsen L, Nettel-Aguirre A, Chaput KH, Tough SC. Hypertensive disorders in pregnancy and child development at 36 months in the All Our Families prospective cohort study. PloS one. 2021;16(12):e0260590. doi: <https://dx.doi.org/10.1371/journal.pone.0260590>.

97. Seidman DS, Laor A, Gale R, Stevenson DK, Mashiach S, Danon YL. Pre-eclampsia and offspring's blood pressure, cognitive ability and physical development at 17-years-of-age. British journal of obstetrics and gynaecology. 1991;98(10):1009-14.

98. Selvaratnam RJ, Wallace EM, Rolnik DL, Davey M-A. Childhood school outcomes for infants born to women with hypertensive disorders during pregnancy. Pregnancy hypertension. 2022;30(101552483):51-8. doi: <https://dx.doi.org/10.1016/j.preghy.2022.08.003>.

99. Silveira RC, Procianoy RS. Growth and neurodevelopment outcome of very low birth weight infants delivered by preeclamptic mothers. ACTA PAEDIATRICA. 2007;96(12):1738-42. doi: 10.1111/j.1651-2227.2007.00552.x. PubMed PMID: WOS:000250915700005.

100. Spinillo A, Gardella B, Preti E, Zanchi S, Stronati M, Fazzi E. Rates of neonatal death and cerebral palsy associated with fetal growth restriction among very low birthweight infants. A temporal analysis. BJOG: An International Journal of Obstetrics & Gynaecology. 2006;113(7):775-80. doi: 10.1111/j.1471-0528.2006.00974.x.

101. Spinillo A, Montanari L, Gardella B, Roccio M, Stronati M, Fazzi E. Infant sex, obstetric risk factors, and 2-year neurodevelopmental outcome among preterm infants. Developmental medicine and child neurology. 2009;51(7):518-25. doi: <https://dx.doi.org/10.1111/j.1469-8749.2009.03273.x>.

102. Sun BZ, Moster D, Harmon QE, Wilcox AJ. Association of Preeclampsia in Term Births With Neurodevelopmental Disorders in Offspring. JAMA psychiatry. 2020;77(8):823-9. doi: <https://dx.doi.org/10.1001/jamapsychiatry.2020.0306>.

103. Sverrisson FA, Bateman BT, Aspelund T, Skulason S, Zoega H. Preeclampsia and academic performance in children: A nationwide study from Iceland. PloS one. 2018;13(11):e0207884. doi: <https://dx.doi.org/10.1371/journal.pone.0207884>.

104. Szymonowicz W, Yu VY. Severe pre-eclampsia and infants of very low birth weight. Arch Dis Child. 1987;62(7):712-6. doi: 10.1136/adc.62.7.712. PubMed PMID: 3632019; PubMed Central PMCID: PMCPMC1779252.

105. Taylor DJ, Howie PW, Davidson J, Davidson D, Drillien CM. Do pregnancy complications contribute to neurodevelopmental disability? Lancet (London, England). 1985;1(8431):713-6.

106. Tuovinen S, Raikkonen K, Kajantie E, Henriksson M, Leskinen JT, Pesonen A-K, et al. Hypertensive disorders in pregnancy and cognitive decline in the offspring up to old age. Neurology. 2012;79(15):1578-82. doi: <https://dx.doi.org/10.1212/WNL.0b013e31826e2606>.

107. Ushida T, Kotani T, Hayakawa M, Hirakawa A, Sadachi R, Nakamura N, et al. Antenatal corticosteroids and preterm offspring outcomes in hypertensive disorders of pregnancy: A Japanese cohort study. Scientific reports. 2020;10(1):9312. doi: <https://dx.doi.org/10.1038/s41598-020-66242-z>.

108. Villamor E, Susser ES, Cnattingius S. Defective placentation syndromes and autism spectrum disorder in the offspring: population-based cohort and sibling-controlled studies. European journal of epidemiology. 2022;37(8):827-36. doi: <https://dx.doi.org/10.1007/s10654-022-00884-3>.

109. Villamor E, Susser ES, Cnattingius S. Defective Placentation Syndromes and Intellectual Disability in the Offspring: Nationwide Cohort and Sibling-Controlled Studies. American journal of epidemiology. 2022;191(9):1557-67. doi: <https://dx.doi.org/10.1093/aje/kwac068>.

110. Walker CK, Krakowiak P, Baker A, Hansen RL, Ozonoff S, Hertz-Picciotto I. Preeclampsia, placental insufficiency, and autism spectrum disorder or developmental delay. JAMA pediatrics. 2015;169(2):154-62. doi: <https://dx.doi.org/10.1001/jamapediatrics.2014.2645>.

111. Wang H, Laszlo KD, Gissler M, Li F, Zhang J, Yu Y, et al. Maternal hypertensive disorders and neurodevelopmental disorders in offspring: a population-based cohort in two Nordic countries. European journal of epidemiology. 2021;36(5):519-30. doi: <https://dx.doi.org/10.1007/s10654-021-00756-2>.

112. Wang L-W, Lin H-C, Tsai M-L, Chang Y-T, Chang Y-C. Maternal hypertensive pregnancy disorders increase childhood intellectual disability hazards independently from preterm birth and small for gestational age. Early human development. 2023;185(edh, 7708381):105856. doi: <https://dx.doi.org/10.1016/j.earlhumdev.2023.105856>.

113. Wang L-W, Lin H-C, Tsai M-L, Chang Y-T, Chang Y-C. Preterm birth and small for gestational age potentiate the association between maternal hypertensive pregnancy and childhood autism spectrum disorder. Scientific reports. 2023;13(1):9606. doi: <https://dx.doi.org/10.1038/s41598-023-36787-w>.

114. Wang H, Yin W, Ma S, Wang P, Zhang L, Li P, et al. Prenatal environmental adversity and child neurodevelopmental delay: the role of maternal low-grade systemic inflammation and maternal anti-inflammatory diet. European Child & Adolescent Psychiatry. 2024;33(6):1771-81. doi: 10.1007/s00787-023-02267-9.

115. Warshafsky C, Pudwell J, Walker M, Wen SW, Smith GN. Prospective assessment of neurodevelopment in children following a pregnancy complicated by severe pre-eclampsia. BMJ Open. 2016;6(7):e010884. Epub 20160707. doi: 10.1136/bmjopen-2015-010884. PubMed PMID: 27388354; PubMed Central PMCID: PMCPMC4947739.

116. Whitehouse AJO, Robinson M, Newnham JP, Pennell CE. Do hypertensive diseases of pregnancy disrupt neurocognitive development in offspring? Paediatric and perinatal epidemiology. 2012;26(2):101-8. doi: <https://dx.doi.org/10.1111/j.1365-3016.2011.01257.x>.

117. Whitely A, Shandley K, Huynh M, Brown CM, Austin DW, Bhowmik J. Brief Report: Pregnancy, Birth and Infant Feeding Practices: A Survey-Based Investigation into Risk Factors for Autism Spectrum Disorder. Journal of autism and developmental disorders. 2022;52(11):5072-8. doi: <https://dx.doi.org/10.1007/s10803-021-05348-3>.

118. Wiggs KK, Cook TE, Lodhawala I, Cleary EN, Yolton K, Becker SP. Setting a research agenda for examining early risk for elevated cognitive disengagement syndrome symptoms using data from the ABCD cohort. Research square. 2024;(101768035). doi: <https://dx.doi.org/10.21203/rs.3.rs-4468007/v1>.

119. Wilson-Costello D, Borawski E, Friedman H, Redline R, Fanaroff AA, Hack M. Perinatal correlates of cerebral palsy and other neurologic impairment among very low birth weight children. Pediatrics. 1998;102(2 Pt 1):315-22.

120. Winer EK, Tejani NA, Atluru V, DiGiuseppe R, Borofsky LG. Four- to seven-year evaluation in two groups of small-for-gestational age infants. Am J Obstet Gynecol. 1982;143(4):425-9. doi: 10.1016/0002-9378(82)90085-0. PubMed PMID: 7091208.

121. Withagen MIJ, Wallenburg HCS, Steegers EAP, Hop WCJ, Visser W. Morbidity and development in childhood of infants born after temporising treatment of early onset pre-eclampsia. BJOG : an international journal of obstetrics and gynaecology. 2005;112(7):910-4.

122. Zen M, Schneuer F, Alahakoon TI, Nassar N, Lee VW. Perinatal and Child Factors Mediate the Association between Preeclampsia and Offspring School Performance. The Journal of pediatrics. 2021;238(jlz, 0375410):153-60.e4. doi: <https://dx.doi.org/10.1016/j.jpeds.2021.06.069>.

123. Barker DJ, Edwards JH. Obstetric complications and school performance. British medical journal. 1967;3(5567):695-9.

124. Check J, Shuster C, Hofheimer J, Camerota M, Dansereau LM, Smith LM, et al. Preeclampsia, Fetal Growth Restriction, and 24-Month Neurodevelopment in Very Preterm Infants. JAMA Network Open. 2024;7(7):e2420382-e. doi: 10.1001/jamanetworkopen.2024.20382.

125. Grace T, Bulsara M, Pennell C, Hands B. Maternal hypertensive diseases negatively affect offspring motor development. Pregnancy hypertension. 2014;4(3):209-14. doi: <https://dx.doi.org/10.1016/j.preghy.2014.04.003>.

126. Iwabuchi T, Takahashi N, Nishimura T, Rahman MS, Harada T, Okumura A, et al. Associations among maternal metabolic conditions, cord serum leptin levels, and autistic symptoms in children. Frontiers in Psychiatry. 2022;12(Agostinelli, C, Marazzi, A, Yohai, VJ, & Randriamiharisoa, A. Robust estimation of the generalized loggamma model: the R package robust log gamma. J Stat Softw. (2016) 70:1-21 <https://dx.doi.org/10.18637/jss.v070.i07Agrawal>, S, Gollapudi, S, Su, H, & Gupt). doi: <https://dx.doi.org/10.3389/fpsyt.2021.816196>.

127. Li X, Eiden RD, Epstein LH, Shenassa ED, Xie C, Wen X. Etiological Subgroups of Small-for-Gestational-Age: Differential Neurodevelopmental Outcomes. PLoS One. 2016;11(8):e0160677. Epub 20160808. doi: 10.1371/journal.pone.0160677. PubMed PMID: 27501456; PubMed Central PMCID: PMCPMC4976943.

128. Ma Q, Cui Y, Han X, Xiong Y, Xu J, Zhao H, et al. Association of maternal hypertension during pregnancy with brain structure and behavioral problems in early adolescence. Eur Child Adolesc Psychiatry. 2024;33(7):2173-87. Epub 20231006. doi: 10.1007/s00787-023-02305-6. PubMed PMID: 37803213.

129. Ratsep MT, Hickman AF, Maser B, Pudwell J, Smith GN, Brien D, et al. Impact of preeclampsia on cognitive function in the offspring. Behavioural brain research. 2016;302(ag3, 8004872):175-81. doi: <https://dx.doi.org/10.1016/j.bbr.2016.01.030>.

130. Schlapbach LJ, Ersch J, Adams M, Bernet V, Bucher HU, Latal B. Impact of chorioamnionitis and preeclampsia on neurodevelopmental outcome in preterm infants below 32 weeks gestational age. Acta Paediatrica. 2010;99(10):1504-9. doi: 10.1111/j.1651-2227.2010.01861.x.

131. Spinillo A, Stronati M, Ometto A, Fazzi E, Lanzi G, Guaschino S. Infant neurodevelopmental outcome in pregnancies complicated by gestational hypertension and intra-uterine growth retardation. J Perinat Med. 1993;21(3):195-203. doi: 10.1515/jpme.1993.21.3.195. PubMed PMID: 8229610.

132. Yoneda N, Yoneda S, Tsuda S, Ito M, Shiozaki A, Niimi H, et al. Pre-eclampsia Complicated With Maternal Renal Dysfunction Is Associated With Poor Neurological Development at 3 Years Old in Children Born Before 34 Weeks of Gestation. Frontiers in pediatrics. 2021;9(101615492):624323. doi: <https://dx.doi.org/10.3389/fped.2021.624323>.
