# Supplementary material for: Hypertensive disorders of pregnancy and childhood neurodevelopment: A systematic review and meta-analysis
Source: PLoS Med. 2025 Sep 10;22(9):e1004558. doi: 10.1371/journal.pmed.1004558 (PMC12422451; doi:10.1371/journal.pmed.1004558)
Supplement: S1 Text — S2 and S3 Appendices. (DOCX) [file pmed.1004558.s005.docx]

**References – S2 and S3 Appendices**

[1-121]

1. Abdelmageed WA, Lapointe A, Brown R, Gorgos A, Luu TM, Beltempo M, et al. Association between maternal hypertension and infant neurodevelopment in extremely preterm infants. JOURNAL OF PERINATOLOGY. 2024;44(4):539-47. doi: 10.1038/s41372-024-01886-7. PubMed PMID: WOS:001153705100003.

2. Almasri J, Barazi A, King KS, Walther-Antonio MRS, Wang Z, Murad MH, et al. Peripartum Antibiotics Exposure and the Risk of Autoimmune and Autism Disorders in the Offspring. Avicenna journal of medicine. 2021;11(3):118-25. doi: <https://dx.doi.org/10.1055/s-0041-1732485>.

3. Amiri S, Malek A, Sadegfard M, Abdi S. Pregnancy-related maternal risk factors of attention-deficit hyperactivity disorder: a case-control study. ISRN Pediatr. 2012;2012:458064. doi: 10.5402/2012/458064. PubMed PMID: 22720167.

4. Arafa A, Mahmoud O, Salah H, Abdelmonem AA, Senosy S. Maternal and neonatal risk factors for autism spectrum disorder: A case-control study from Egypt. PloS one. 2022;17(6):e0269803. doi: <https://dx.doi.org/10.1371/journal.pone.0269803>.

5. Arun P, Azad C, Kaur G, Sharma P. A Community-Based Study of Antenatal and Neonatal Risk Factors in Autism Spectrum Disorder. The primary care companion for CNS disorders. 2023;25(2). doi: <https://dx.doi.org/10.4088/PCC.22m03339>.

6. Avorgbedor F, Silva S, Merwin E, Blumenthal JA, Holditch-Davis D. Health, Physical Growth, and Neurodevelopmental Outcomes in Preterm Infants of Women With Hypertensive Disorders of Pregnancy. JOGNN: Journal of Obstetric, Gynecologic & Neonatal Nursing. 2019;48(1):69-77. doi: 10.1016/j.jogn.2018.10.003.

7. Ayala NK, Schlichting LE, Kempner M, Clark MA, Vivier PM, Viner-Brown SI, et al. Association between maternal hypertensive disorders, fetal growth and childhood learning outcomes. Pregnancy hypertension. 2021;25(101552483):249-54. doi: <https://dx.doi.org/10.1016/j.preghy.2021.07.242>.

8. Bajalan Z, Alimoradi Z. Risk factors of developmental delay among infants aged 6-18 months. EARLY CHILD DEVELOPMENT AND CARE. 2020;190(11):1691-9. doi: 10.1080/03004430.2018.1547714. PubMed PMID: WOS:000576223900003.

9. Beer RJ, Cnattingius S, Susser ES, Villamor E. Associations of preterm birth, small-for-gestational age, preeclampsia and placental abruption with attention-deficit/hyperactivity disorder in the offspring: Nationwide cohort and sibling-controlled studies. Acta paediatrica (Oslo, Norway : 1992). 2022;111(8):1546-55. doi: <https://dx.doi.org/10.1111/apa.16375>.

10. Beukers F, Aarnoudse-Moens CSH, van Weissenbruch MM, Ganzevoort W, van Goudoever JB, van Wassenaer-Leemhuis AG. Fetal Growth Restriction with Brain Sparing: Neurocognitive and Behavioral Outcomes at 12 Years of Age. The Journal of pediatrics. 2017;188(jlz, 0375410):103-9.e2. doi: <https://dx.doi.org/10.1016/j.jpeds.2017.06.003>.

11. Bharadwaj SK, Vishnu Bhat B, Vickneswaran V, Adhisivam B, Bobby Z, Habeebullah S. Oxidative Stress, Antioxidant Status and Neurodevelopmental Outcome in Neonates Born to Pre-eclamptic Mothers. Indian journal of pediatrics. 2018;85(5):351-7. doi: <https://dx.doi.org/10.1007/s12098-017-2560-5>.

12. Bilder D, Pinborough-Zimmerman J, Miller J, McMahon W. Prenatal, perinatal, and neonatal factors associated with autism spectrum disorders. Pediatrics. 2009;123(5):1293-300. doi: 10.1542/peds.2008-0927. PubMed PMID: 19403494.

13. Böhm S, Curran EA, Kenny LC, O'Keeffe GW, Murray D, Khashan AS. The Effect of Hypertensive Disorders of Pregnancy on the Risk of ADHD in the Offspring. J Atten Disord. 2019;23(7):692-701. Epub 20170204. doi: 10.1177/1087054717690230. PubMed PMID: 28162026.

14. Bolk J, Kallen K, Farooqi A, Hafstrom M, Fellman V, Aden U, et al. Perinatal risk factors for developmental coordination disorder in children born extremely preterm. Acta Paediatrica. 2023;112(4):675-85. doi: <https://dx.doi.org/10.1111/apa.16651>.

15. Brand JS, Lawlor DA, Larsson H, Montgomery S. Association Between Hypertensive Disorders of Pregnancy and Neurodevelopmental Outcomes Among Offspring. JAMA pediatrics. 2021;175(6):577-85. doi: <https://dx.doi.org/10.1001/jamapediatrics.2020.6856>.

16. Buchmayer S, Johansson S, Johansson A, Hultman CM, Sparen P, Cnattingius S. Can association between preterm birth and autism be explained by maternal or neonatal morbidity? Pediatrics. 2009;124(5):e817-25. doi: <https://dx.doi.org/10.1542/peds.2008-3582>.

17. Burstyn I, Sithole F, Zwaigenbaum L. Autism spectrum disorders, maternal characteristics and obstetric complications among singletons born in Alberta, Canada. Chronic diseases in Canada. 2010;30(4):125-34. PubMed PMID: 20946713.

18. Çak HT, Gökler B. Attention deficit hyperactivity disorder and associated perinatal risk factors in preterm children. Turkish Archives of Pediatrics. 2013:315-22.

19. Carter S, Lin JC, Chow T, Martinez MP, Qiu C, Feldman RK, et al. Preeclampsia Onset, Days to Delivery, and Autism Spectrum Disorders in Offspring: Clinical Birth Cohort Study. JMIR public health and surveillance. 2024;10(101669345):e47396. doi: <https://dx.doi.org/10.2196/47396>.

20. Carter SA, Lin JC, Chow T, Yu X, Rahman MM, Martinez MP, et al. Maternal obesity, diabetes, preeclampsia, and asthma during pregnancy and likelihood of autism spectrum disorder with gastrointestinal disturbances in offspring. Autism : the international journal of research and practice. 2023;27(4):916-26. doi: <https://dx.doi.org/10.1177/13623613221118430>.

21. Chan SE, Pudwell J, Smith GN. Effects of Preeclampsia on Maternal and Pediatric Health at 11 Years Postpartum. American journal of perinatology. 2019;36(8):806-11. doi: <https://dx.doi.org/10.1055/s-0038-1675374>.

22. Chang H-Y, Chen C-P, Sun F-J, Chen C-Y. Influence of pre-eclampsia on 2-year neurodevelopmental outcome of very-low-birth-weight infants. International journal of gynaecology and obstetrics: the official organ of the International Federation of Gynaecology and Obstetrics. 2023;161(3):979-88. doi: <https://dx.doi.org/10.1002/ijgo.14614>.

23. Chen G, Ishikuro M, Ohseto H, Murakami K, Noda A, Shinoda G, et al. Hypertensive disorders of pregnancy, neonatal outcomes and offspring developmental delay in Japan: The Tohoku Medical Megabank Project Birth and Three-Generation Cohort Study. Acta obstetricia et gynecologica Scandinavica. 2024;103(6):1192-200. doi: <https://dx.doi.org/10.1111/aogs.14820>.

24. Chen K-R, Yu T, Kang L, Lien Y-J, Kuo P-L. Childhood neurodevelopmental disorders and maternal hypertensive disorder of pregnancy. Developmental medicine and child neurology. 2021;63(9):1107-13. doi: <https://dx.doi.org/10.1111/dmcn.14893>.

25. Chen Z, Li R, Liu H, Duan J, Yao C, Yang R, et al. Impact of maternal hypertensive disorders on offspring's neurodevelopment: a longitudinal prospective cohort study in China. Pediatric research. 2020;88(4):668-75. doi: <https://dx.doi.org/10.1038/s41390-020-0794-9>.

26. Cheng S-W, Chou H-C, Tsou K-I, Fang L-J, Tsao P-N. Delivery before 32 weeks of gestation for maternal pre-eclampsia: neonatal outcome and 2-year developmental outcome. Early human development. 2004;76(1):39-46.

27. Chien Y-L, Chou M-C, Chou W-J, Wu Y-Y, Tsai W-C, Chiu Y-N, et al. Prenatal and perinatal risk factors and the clinical implications on autism spectrum disorder. Autism : the international journal of research and practice. 2019;23(3):783-91. doi: <https://dx.doi.org/10.1177/1362361318772813>.

28. Chowdhury MAK, Hardin JW, Love BL, Merchant AT, McDermott S. Relationship of nonsteroidal anti-inflammatory drug use during pregnancy with autism spectrum disorder and intellectual disability among offspring. Journal of Women's Health. 2023;32(3):356-65. doi: <https://dx.doi.org/10.1089/jwh.2022.0113>.

29. Christians JK, Chow NA. Are there sex differences in fetal growth strategies and in the long-term effects of pregnancy complications on cognitive functioning? JOURNAL OF DEVELOPMENTAL ORIGINS OF HEALTH AND DISEASE. 2022;13(6):766-78. doi: 10.1017/S2040174422000204. PubMed PMID: WOS:000783015600001.

30. Cochran DM, Jensen ET, Frazier JA, Jalnapurkar I, Kim S, Roell KR, et al. Association of prenatal modifiable risk factors with attention-deficit hyperactivity disorder outcomes at age 10 and 15 in an extremely low gestational age cohort. Front Hum Neurosci. 2022;16:911098. Epub 20221020. doi: 10.3389/fnhum.2022.911098. PubMed PMID: 36337853; PubMed Central PMCID: PMCPMC9630552.

31. Cordero C, Windham GC, Schieve LA, Fallin MD, Croen LA, Siega-Riz AM, et al. Maternal diabetes and hypertensive disorders in association with autism spectrum disorder. Autism research : official journal of the International Society for Autism Research. 2019;12(6):967-75. doi: <https://dx.doi.org/10.1002/aur.2105>.

32. Cui L, Du W, Xu N, Dong J, Xia B, Ma J, et al. Impact of MicroRNAs in interaction with environmental factors on autism spectrum disorder: An exploratory pilot study. Frontiers in Psychiatry. 2021;12(Beversdorf, DQ, Stevens, HE, & Jones, KL. Prenatal Stress, Maternal immune dysregulation, and their association with autism spectrum disorders. Curr Psychiatry Rep. (2018) 20:76 <https://pubmed.ncbi.nlm.nih.gov/30094645> <https://dx.doi.org/10.1007/s11920-01>). doi: <https://dx.doi.org/10.3389/fpsyt.2021.715481>.

33. Curran EA, O'Keeffe GW, Looney AM, Moloney G, Hegarty SV, Murray DM, et al. Exposure to Hypertensive Disorders of Pregnancy Increases the Risk of Autism Spectrum Disorder in Affected Offspring. Mol Neurobiol. 2018;55(7):5557-64. doi: 10.1007/s12035-017-0794-x. PubMed PMID: 28975539.

34. Dachew BA, Scott JG, Mamun A, Alati R. Pre-eclampsia and the risk of attention-deficit/hyperactivity disorder in offspring: Findings from the ALSPAC birth cohort study. Psychiatry Res. 2019;272:392-7. Epub 20181225. doi: 10.1016/j.psychres.2018.12.123. PubMed PMID: 30605798.

35. Dachew BA, Scott JG, Mamun A, Fetene DM, Alati R. Maternal hypertensive disorders during pregnancy and the trajectories of offspring emotional and behavioral problems: the ALSPAC birth cohort study. Annals of epidemiology. 2021;53(9100013, bx8):63-8.e1. doi: <https://dx.doi.org/10.1016/j.annepidem.2020.08.015>.

36. Dodds L, Fell DB, Shea S, Armson BA, Allen AC, Bryson S. The Role of Prenatal, Obstetric and Neonatal Factors in the Development of Autism. Journal of Autism and Developmental Disorders. 2011;41(7):891-902. doi: 10.1007/s10803-010-1114-8.

37. Duko B, Gebremedhin AT, Tessema GA, Dunne J, Alati R, Pereira G. The effects of pre-eclampsia on social and emotional developmental vulnerability in children at age five in Western Australia: A population data linkage study. Journal of affective disorders. 2024;352(h3v, 7906073):349-56. doi: <https://dx.doi.org/10.1016/j.jad.2024.02.042>.

38. Ehrenstein V, Rothman KJ, Pedersen L, Hatch EE, Sorensen HT. Pregnancy-associated hypertensive disorders and adult cognitive function among Danish conscripts. American journal of epidemiology. 2009;170(8):1025-31. doi: <https://dx.doi.org/10.1093/aje/kwp223>.

39. Fast K, Wentz E, Roswall J, Strandberg M, Bergman S, Dahlgren J. Prevalence of attention-deficit/hyperactivity disorder and autism in 12-year-old children: A population-based cohort. Developmental medicine and child neurology. 2024;66(4):493-500. doi: <https://dx.doi.org/10.1111/dmcn.15757>.

40. Fitton CA, Fleming M, Aucott L, Pell JP, Mackay DF, McLay JS. Congenital defects and early childhood outcomes following in-utero exposure to antihypertensive medication. Journal of hypertension. 2021;39(3):581-8. doi: <https://dx.doi.org/10.1097/HJH.0000000000002670>.

41. Getahun D, Rhoads GG, Demissie K, Lu S-E, Quinn VP, Fassett MJ, et al. In utero exposure to ischemic-hypoxic conditions and attention-deficit/hyperactivity disorder. Pediatrics. 2013;131(1):e53-61. doi: <https://dx.doi.org/10.1542/peds.2012-1298>.

42. Girchenko P, Lahti-Pulkkinen M, Lahti J, Pesonen AK, Hämäläinen E, Villa PM, et al. Neonatal regulatory behavior problems are predicted by maternal early pregnancy overweight and obesity: findings from the prospective PREDO Study. Pediatr Res. 2018;84(6):875-81. Epub 20181010. doi: 10.1038/s41390-018-0199-1. PubMed PMID: 30305694.

43. Girchenko P, Tuovinen S, Lahti-Pulkkinen M, Lahti J, Savolainen K, Heinonen K, et al. Maternal early pregnancy obesity and related pregnancy and pre-pregnancy disorders: associations with child developmental milestones in the prospective PREDO Study. International journal of obesity (2005). 2018;42(5):995-1007. doi: <https://dx.doi.org/10.1038/s41366-018-0061-x>.

44. Glasson EJ, Bower C, Petterson B, de Klerk N, Chaney G, Hallmayer JF. Perinatal Factors and the Development of Autism: A Population Study. Archives of General Psychiatry. 2004;61(6):618-27. doi: 10.1001/archpsyc.61.6.618.

45. Golmirzaei J, Namazi S, Amiri S, Zare S, Rastikerdar N, Hesam AA, et al. Evaluation of attention-deficit hyperactivity disorder risk factors. Int J Pediatr. 2013;2013:953103. doi: 10.1155/2013/953103. PubMed PMID: 24319465.

46. Gray PH, Hurley TM, Rogers YM, O'Callaghan MJ, Tudehope DI, Burns YR, et al. Survival and neonatal and neurodevelopmental outcome of 24-29 week gestation infants according to primary cause of preterm delivery. The Australian & New Zealand journal of obstetrics & gynaecology. 1997;37(2):161-8.

47. Gray PH, O'Callaghan MJ, Mohay HA, Burns YR, King JF. Maternal hypertension and neurodevelopmental outcome in very preterm infants. Archives of disease in childhood Fetal and neonatal edition. 1998;79(2):F88-93.

48. Griffith MI, Mann JR, McDermott S. The risk of intellectual disability in children born to mothers with preeclampsia or eclampsia with partial mediation by low birth weight. Hypertension in pregnancy. 2011;30(1):108-15. doi: <https://dx.doi.org/10.3109/10641955.2010.507837>.

49. He F, Li QP, Li NP, Yao L, Ma XW, Feng ZC. Analysis of high-risk factors and effect of early intervention on preterm infant neurodevelopment. INTERNATIONAL JOURNAL OF CLINICAL AND EXPERIMENTAL MEDICINE. 2017;10(3):5372-80. PubMed PMID: WOS:000400552900133.

50. Heikura U, Hartikainen A-L, Nordstrom T, Pouta A, Taanila A, Jarvelin M-R. Maternal hypertensive disorders during pregnancy and mild cognitive limitations in the offspring. Paediatric and perinatal epidemiology. 2013;27(2):188-98. doi: <https://dx.doi.org/10.1111/ppe.12028>.

51. Hisle-Gorman E, Susi A, Stokes T, Gorman G, Erdie-Lalena C, Nylund CM. Prenatal, perinatal, and neonatal risk factors of autism spectrum disorder. Pediatric research. 2018;84(2):190-8. doi: <https://dx.doi.org/10.1038/pr.2018.23>.

52. Huang B, Wang Y, Jiang Y, Lv H, Jiang T, Qiu Y, et al. Association of maternal hypertensive disorders in pregnancy with infant neurodevelopment. Journal of biomedical research. 2023;37(6):479-91. doi: <https://dx.doi.org/10.7555/JBR.37.20230074>.

53. Hultman CM, Sparén P, Cnattingius S. Perinatal risk factors for infantile autism. Epidemiology. 2002;13(4):417-23. doi: 10.1097/00001648-200207000-00009. PubMed PMID: 12094096.

54. Ishikuro M, Murakami K, Yokozeki F, Onuma T, Noda A, Ueno F, et al. Hypertension in pregnancy as a possible factor for child autistic behavior at two years old. Pregnancy hypertension. 2021;25(101552483):88-90. doi: <https://dx.doi.org/10.1016/j.preghy.2021.05.020>.

55. Kodesh A, Levine SZ, Khachadourian V, Rahman R, Schlessinger A, O'Reilly PF, et al. Maternal health around pregnancy and autism risk: A diagnosis-wide, population-based study. Psychological Medicine. 2022;52(16):4076-84. doi: <https://dx.doi.org/10.1017/S0033291721001021>.

56. Kong L, Chen X, Liang Y, Forsell Y, Gissler M, Lavebratt C. Association of Preeclampsia and Perinatal Complications With Offspring Neurodevelopmental and Psychiatric Disorders. JAMA network open. 2022;5(1):e2145719. doi: <https://dx.doi.org/10.1001/jamanetworkopen.2021.45719>.

57. Koparkar S, Srivastava L, Randhir K, Dangat K, Pisal H, Kadam V, et al. Cognitive function and behavioral problems in children born to mothers with preeclampsia: an Indian study. Child neuropsychology : a journal on normal and abnormal development in childhood and adolescence. 2022;28(3):337-54. doi: <https://dx.doi.org/10.1080/09297049.2021.1978418>.

58. Korzeniewski SJ, Pinto-Martin JA, Whitaker AH, Feldman JF, Lorenz JM, Levy SE, et al. Association between transient hypothyroxinaemia of prematurity and adult autism spectrum disorder in a low-birthweight cohort: an exploratory study. Paediatric and perinatal epidemiology. 2013;27(2):182-7. doi: <https://dx.doi.org/10.1111/ppe.12034>.

59. Krakowiak P, Walker CK, Bremer AA, Baker AS, Ozonoff S, Hansen RL, et al. Maternal metabolic conditions and risk for autism and other neurodevelopmental disorders. Pediatrics. 2012;129(5):e1121-8. Epub 20120409. doi: 10.1542/peds.2011-2583. PubMed PMID: 22492772; PubMed Central PMCID: PMCPMC3340592.

60. Lahti-Pulkkinen M, Girchenko P, Tuovinen S, Sammallahti S, Reynolds RM, Lahti J, et al. Maternal Hypertensive Pregnancy Disorders and Mental Disorders in Children. Hypertension (Dallas, Tex : 1979). 2020;75(6):1429-38. doi: <https://dx.doi.org/10.1161/HYPERTENSIONAHA.119.14140>.

61. Larsson HJ, Eaton WW, Madsen KM, Vestergaard M, Olesen AV, Agerbo E, et al. Risk factors for autism: perinatal factors, parental psychiatric history, and socioeconomic status. Am J Epidemiol. 2005;161(10):916-25; discussion 26. doi: 10.1093/aje/kwi123. PubMed PMID: 15870155.

62. Lee S, Han Y, Lim MK, Lee HJ. Impact of moderate-to-late preterm birth on neurodevelopmental outcomes in young children: Results from retrospective longitudinal follow-up with nationally representative data. PloS one. 2023;18(11):e0294435. doi: <https://dx.doi.org/10.1371/journal.pone.0294435>.

63. Leitner Y, Harel S, Geva R, Eshel R, Yaffo A, Many A. The neurocognitive outcome of IUGR children born to mothers with and without preeclampsia. The journal of maternal-fetal & neonatal medicine : the official journal of the European Association of Perinatal Medicine, the Federation of Asia and Oceania Perinatal Societies, the International Society of Perinatal Obstetricians. 2012;25(11):2206-8. doi: <https://dx.doi.org/10.3109/14767058.2012.684164>.

64. Leonard H, de Klerk N, Bourke J, Bower C. Maternal Health in Pregnancy and Intellectual Disability in the Offspring: A Population-Based Study. Annals of Epidemiology. 2006;16(6):448-54. doi: <https://doi.org/10.1016/j.annepidem.2005.05.002>.

65. Liu L, Lin Z, Zheng B, Wang L, Zou J, Wu S, et al. Reduced Intellectual Ability in Offspring Born from Preeclamptic Mothers: A Prospective Cohort Study. Risk management and healthcare policy. 2020;13(101566264):2037-46. doi: <https://dx.doi.org/10.2147/RMHP.S277521>.

66. Lyall K, Ning X, Aschner JL, Avalos LA, Bennett DH, Bilder DA, et al. Cardiometabolic Pregnancy Complications in Association With Autism-Related Traits as Measured by the Social Responsiveness Scale in ECHO. American journal of epidemiology. 2022;191(8):1407-19. doi: <https://dx.doi.org/10.1093/aje/kwac061>.

67. Maher GM, Dalman C, O'Keeffe GW, Kearney PM, McCarthy FP, Kenny LC, et al. Association between preeclampsia and attention-deficit hyperactivity disorder: a population-based and sibling-matched cohort study. Acta psychiatrica Scandinavica. 2020;142(4):275-83. doi: <https://dx.doi.org/10.1111/acps.13162>.

68. Maher GM, McCarthy FP, Khashan AS. Hypertensive Disorders of Pregnancy and Behavioural Outcomes in the Offspring: Findings from the Millennium Cohort Study. Journal of affective disorders. 2021;287(h3v, 7906073):222-8. doi: <https://dx.doi.org/10.1016/j.jad.2021.03.040>.

69. Maher GM, O'Keeffe GW, Dalman C, Kearney PM, McCarthy FP, Kenny LC, et al. Association between preeclampsia and autism spectrum disorder: a population-based study. Journal of child psychology and psychiatry, and allied disciplines. 2020;61(2):131-9. doi: <https://dx.doi.org/10.1111/jcpp.13127>.

70. Maher GM, O'Keeffe GW, O'Keeffe LM, Matvienko-Sikar K, Dalman C, Kearney PM, et al. The Association Between Preeclampsia and Childhood Development and Behavioural Outcomes. Maternal and child health journal. 2020;24(6):727-38. doi: <https://dx.doi.org/10.1007/s10995-020-02921-7>.

71. Mann JR, McDermott S. Are maternal genitourinary infection and pre-eclampsia associated with ADHD in school-aged children? Journal of attention disorders. 2011;15(8):667-73. doi: <https://dx.doi.org/10.1177/1087054710370566>.

72. Mann JR, McDermott S, Bao H, Hardin J, Gregg A. Pre-eclampsia, birth weight, and autism spectrum disorders. Journal of autism and developmental disorders. 2010;40(5):548-54. doi: <https://dx.doi.org/10.1007/s10803-009-0903-4>.

73. Mann JR, McDermott S, Griffith MI, Hardin J, Gregg A. Uncovering the complex relationship between pre-eclampsia, preterm birth and cerebral palsy. Paediatric and perinatal epidemiology. 2011;25(2):100-10. doi: <https://dx.doi.org/10.1111/j.1365-3016.2010.01157.x>.

74. Manovitch Z, Morag I, Simchen MJ. Neurodevelopmental outcomes of preterm infants born to preeclamptic mothers - A case-control study. EUROPEAN JOURNAL OF OBSTETRICS & GYNECOLOGY AND REPRODUCTIVE BIOLOGY. 2022;270:6-10. doi: 10.1016/j.ejogrb.2021.12.036. PubMed PMID: WOS:000774443600002.

75. Many A, Fattal A, Leitner Y, Kupferminc MJ, Harel S, Jaffa A. Neurodevelopmental and cognitive assessment of children born growth restricted to mothers with and without preeclampsia. Hypertension in pregnancy. 2003;22(1):25-9.

76. Many A, Fattal-Valevski A, Leitner Y. Neurodevelopmental and cognitive assessment of 6-year-old children born growth restricted. International journal of gynaecology and obstetrics: the official organ of the International Federation of Gynaecology and Obstetrics. 2005;89(1):55-6.

77. Matić M, Inati V, Abdel ‐ Latif ME, Kent AL. Maternal hypertensive disorders are associated with increased use of respiratory support but not chronic lung disease or poorer neurodevelopmental outcomes in preterm neonates at <29 weeks of gestation. Journal of Paediatrics & Child Health. 2017;53(4):391-8. doi: 10.1111/jpc.13430.

78. McCowan LME, Pryor J, Harding JE. Perinatal predictors of neurodevelopmental outcome in small-for-gestational-age children at 18 months of age. American journal of obstetrics and gynecology. 2002;186(5):1069-75.

79. Moore GS, Kneitel AW, Walker CK, Gilbert WM, Xing G. Autism risk in small- and large-for-gestational-age infants. Am J Obstet Gynecol. 2012;206(4):314.e1-9. doi: 10.1016/j.ajog.2012.01.044. PubMed PMID: 22464070.

80. Mor O, Stavsky M, Yitshak-Sade M, Mastrolia SA, Beer-Weisel R, Rafaeli-Yehudai T, et al. Early onset preeclampsia and cerebral palsy: a double hit model? American journal of obstetrics and gynecology. 2016;214(1):105.e1-9. doi: <https://dx.doi.org/10.1016/j.ajog.2015.08.020>.

81. Morsing E, Marsal K. Pre-eclampsia- an additional risk factor for cognitive impairment at school age after intrauterine growth restriction and very preterm birth. Early human development. 2014;90(2):99-101. doi: <https://dx.doi.org/10.1016/j.earlhumdev.2013.12.002>.

82. Mrozek-Budzyn D, Majewska R, Kieltyka A. Prenatal, perinatal and neonatal risk factors for autism - study in Poland. Central European Journal of Medicine. 2013;8(4):424-30. doi: 10.2478/s11536-013-0174-5.

83. Murphy DJ, Sellers S, MacKenzie IZ, Yudkin PL, Johnson AM. Case-control study of antenatal and intrapartum risk factors for cerebral palsy in very preterm singleton babies. Lancet. 1995;346(8988):1449-54. doi: 10.1016/s0140-6736(95)92471-x.

84. Nahum Sacks K, Friger M, Shoham-Vardi I, Sergienko R, Spiegel E, Landau D, et al. Long-term neuropsychiatric morbidity in children exposed prenatally to preeclampsia. Early human development. 2019;130(edh, 7708381):96-100. doi: <https://dx.doi.org/10.1016/j.earlhumdev.2019.01.016>.

85. Nath S, Roy R, Mukherjee S. Perinatal complications associated with autism--a case control study in a neurodevelopment and early intervention clinic. Journal of the Indian Medical Association. 2012;110(8):526-9.

86. Nielsen TC, Nassar N, Shand AW, Jones HF, Han VX, Patel S, et al. Association between cumulative maternal exposures related to inflammation and child attention-deficit/hyperactivity disorder: A cohort study. PAEDIATRIC AND PERINATAL EPIDEMIOLOGY. 2024;38(3):241-50. doi: 10.1111/ppe.13022. PubMed PMID: WOS:001108723800001.

87. Noda M, Yoshida S, Mishina H, Matsubayashi K, Kawakami K. Association between maternal hypertensive disorders of pregnancy and child neurodevelopment at 3 years of age: a retrospective cohort study. Journal of developmental origins of health and disease. 2021;12(3):428-35. doi: <https://dx.doi.org/10.1017/S2040174420000586>.

88. Palatnik A, Mele L, Casey BM, Varner MW, Sorokin Y, Reddy UM, et al. Association between Hypertensive Disorders of Pregnancy and Long-Term Neurodevelopmental Outcomes in the Offspring. American journal of perinatology. 2022;39(9):921-9. doi: <https://dx.doi.org/10.1055/a-1692-0659>.

89. Palmer L, Blair E, Petterson B, Burton P. ANTENATAL ANTECEDENTS OF MODERATE AND SEVERE CEREBRAL-PALSY. PAEDIATRIC AND PERINATAL EPIDEMIOLOGY. 1995;9(2):171-84. doi: 10.1111/j.1365-3016.1995.tb00132.x. PubMed PMID: WOS:A1995QR95100007.

90. Pohlabeln H, Rach S, De Henauw S, Eiben G, Gwozdz W, Hadjigeorgiou C, et al. Further evidence for the role of pregnancy-induced hypertension and other early life influences in the development of ADHD: results from the IDEFICS study. European child & adolescent psychiatry. 2017;26(8):957-67. doi: <https://dx.doi.org/10.1007/s00787-017-0966-2>.

91. Polo-Kantola P, Lampi KM, Hinkka-Yli-Salomaki S, Gissler M, Brown AS, Sourander A. Obstetric risk factors and autism spectrum disorders in Finland. The Journal of pediatrics. 2014;164(2):358-65. doi: <https://dx.doi.org/10.1016/j.jpeds.2013.09.044>.

92. Raz R, Roberts AL, Lyall K, Hart JE, Just AC, Laden F, et al. Autism spectrum disorder and particulate matter air pollution before, during, and after pregnancy: a nested case-control analysis within the Nurses' Health Study II Cohort. Environ Health Perspect. 2015;123(3):264-70. doi: 10.1289/ehp.1408133. PubMed PMID: 25522338.

93. Robinson M, Mattes E, Oddy WH, de Klerk NH, Li J, McLean NJ, et al. Hypertensive diseases of pregnancy and the development of behavioral problems in childhood and adolescence: the Western Australian Pregnancy Cohort Study. The Journal of pediatrics. 2009;154(2):218-24. doi: <https://dx.doi.org/10.1016/j.jpeds.2008.07.061>.

94. Sabino AT, Souza E, Goulart AL, Lima AM, Sass N. High Blood Pressure during Pregnancy is not a Protective Factor for Preterm Infants with Very Low Birth Weight. A Case-Control Study. Rev Bras Ginecol Obstet. 2017;39(4):155-61. Epub 20170413. doi: 10.1055/s-0037-1601883. PubMed PMID: 28407656; PubMed Central PMCID: PMCPMC10309464.

95. Scime NV, Hetherington E, Tomfohr-Madsen L, Nettel-Aguirre A, Chaput KH, Tough SC. Hypertensive disorders in pregnancy and child development at 36 months in the All Our Families prospective cohort study. PloS one. 2021;16(12):e0260590. doi: <https://dx.doi.org/10.1371/journal.pone.0260590>.

96. Seidman DS, Laor A, Gale R, Stevenson DK, Mashiach S, Danon YL. Pre-eclampsia and offspring's blood pressure, cognitive ability and physical development at 17-years-of-age. British journal of obstetrics and gynaecology. 1991;98(10):1009-14.

97. Selvaratnam RJ, Wallace EM, Rolnik DL, Davey M-A. Childhood school outcomes for infants born to women with hypertensive disorders during pregnancy. Pregnancy hypertension. 2022;30(101552483):51-8. doi: <https://dx.doi.org/10.1016/j.preghy.2022.08.003>.

98. Silveira RC, Procianoy RS. Growth and neurodevelopment outcome of very low birth weight infants delivered by preeclamptic mothers. ACTA PAEDIATRICA. 2007;96(12):1738-42. doi: 10.1111/j.1651-2227.2007.00552.x. PubMed PMID: WOS:000250915700005.

99. Spinillo A, Gardella B, Preti E, Zanchi S, Stronati M, Fazzi E. Rates of neonatal death and cerebral palsy associated with fetal growth restriction among very low birthweight infants. A temporal analysis. BJOG: An International Journal of Obstetrics & Gynaecology. 2006;113(7):775-80. doi: 10.1111/j.1471-0528.2006.00974.x.

100. Spinillo A, Montanari L, Gardella B, Roccio M, Stronati M, Fazzi E. Infant sex, obstetric risk factors, and 2-year neurodevelopmental outcome among preterm infants. Developmental medicine and child neurology. 2009;51(7):518-25. doi: <https://dx.doi.org/10.1111/j.1469-8749.2009.03273.x>.

101. Sun BZ, Moster D, Harmon QE, Wilcox AJ. Association of Preeclampsia in Term Births With Neurodevelopmental Disorders in Offspring. JAMA psychiatry. 2020;77(8):823-9. doi: <https://dx.doi.org/10.1001/jamapsychiatry.2020.0306>.

102. Sverrisson FA, Bateman BT, Aspelund T, Skulason S, Zoega H. Preeclampsia and academic performance in children: A nationwide study from Iceland. PloS one. 2018;13(11):e0207884. doi: <https://dx.doi.org/10.1371/journal.pone.0207884>.

103. Szymonowicz W, Yu VY. Severe pre-eclampsia and infants of very low birth weight. Arch Dis Child. 1987;62(7):712-6. doi: 10.1136/adc.62.7.712. PubMed PMID: 3632019; PubMed Central PMCID: PMCPMC1779252.

104. Taylor DJ, Howie PW, Davidson J, Davidson D, Drillien CM. Do pregnancy complications contribute to neurodevelopmental disability? Lancet (London, England). 1985;1(8431):713-6.

105. Tuovinen S, Raikkonen K, Kajantie E, Henriksson M, Leskinen JT, Pesonen A-K, et al. Hypertensive disorders in pregnancy and cognitive decline in the offspring up to old age. Neurology. 2012;79(15):1578-82. doi: <https://dx.doi.org/10.1212/WNL.0b013e31826e2606>.

106. Ushida T, Kotani T, Hayakawa M, Hirakawa A, Sadachi R, Nakamura N, et al. Antenatal corticosteroids and preterm offspring outcomes in hypertensive disorders of pregnancy: A Japanese cohort study. Scientific reports. 2020;10(1):9312. doi: <https://dx.doi.org/10.1038/s41598-020-66242-z>.

107. Villamor E, Susser ES, Cnattingius S. Defective placentation syndromes and autism spectrum disorder in the offspring: population-based cohort and sibling-controlled studies. European journal of epidemiology. 2022;37(8):827-36. doi: <https://dx.doi.org/10.1007/s10654-022-00884-3>.

108. Villamor E, Susser ES, Cnattingius S. Defective Placentation Syndromes and Intellectual Disability in the Offspring: Nationwide Cohort and Sibling-Controlled Studies. American journal of epidemiology. 2022;191(9):1557-67. doi: <https://dx.doi.org/10.1093/aje/kwac068>.

109. Walker CK, Krakowiak P, Baker A, Hansen RL, Ozonoff S, Hertz-Picciotto I. Preeclampsia, placental insufficiency, and autism spectrum disorder or developmental delay. JAMA pediatrics. 2015;169(2):154-62. doi: <https://dx.doi.org/10.1001/jamapediatrics.2014.2645>.

110. Wang H, Laszlo KD, Gissler M, Li F, Zhang J, Yu Y, et al. Maternal hypertensive disorders and neurodevelopmental disorders in offspring: a population-based cohort in two Nordic countries. European journal of epidemiology. 2021;36(5):519-30. doi: <https://dx.doi.org/10.1007/s10654-021-00756-2>.

111. Wang H, Yin W, Ma S, Wang P, Zhang L, Li P, et al. Prenatal environmental adversity and child neurodevelopmental delay: the role of maternal low-grade systemic inflammation and maternal anti-inflammatory diet. European Child & Adolescent Psychiatry. 2024;33(6):1771-81. doi: 10.1007/s00787-023-02267-9.

112. Wang L-W, Lin H-C, Tsai M-L, Chang Y-T, Chang Y-C. Maternal hypertensive pregnancy disorders increase childhood intellectual disability hazards independently from preterm birth and small for gestational age. Early human development. 2023;185(edh, 7708381):105856. doi: <https://dx.doi.org/10.1016/j.earlhumdev.2023.105856>.

113. Wang L-W, Lin H-C, Tsai M-L, Chang Y-T, Chang Y-C. Preterm birth and small for gestational age potentiate the association between maternal hypertensive pregnancy and childhood autism spectrum disorder. Scientific reports. 2023;13(1):9606. doi: <https://dx.doi.org/10.1038/s41598-023-36787-w>.

114. Warshafsky C, Pudwell J, Walker M, Wen SW, Smith GN. Prospective assessment of neurodevelopment in children following a pregnancy complicated by severe pre-eclampsia. BMJ Open. 2016;6(7):e010884. Epub 20160707. doi: 10.1136/bmjopen-2015-010884. PubMed PMID: 27388354; PubMed Central PMCID: PMCPMC4947739.

115. Whitehouse AJO, Robinson M, Newnham JP, Pennell CE. Do hypertensive diseases of pregnancy disrupt neurocognitive development in offspring? Paediatric and perinatal epidemiology. 2012;26(2):101-8. doi: <https://dx.doi.org/10.1111/j.1365-3016.2011.01257.x>.

116. Whitely A, Shandley K, Huynh M, Brown CM, Austin DW, Bhowmik J. Brief Report: Pregnancy, Birth and Infant Feeding Practices: A Survey-Based Investigation into Risk Factors for Autism Spectrum Disorder. Journal of autism and developmental disorders. 2022;52(11):5072-8. doi: <https://dx.doi.org/10.1007/s10803-021-05348-3>.

117. Wiggs KK, Cook TE, Lodhawala I, Cleary EN, Yolton K, Becker SP. Setting a research agenda for examining early risk for elevated cognitive disengagement syndrome symptoms using data from the ABCD cohort. Research square. 2024;(101768035). doi: <https://dx.doi.org/10.21203/rs.3.rs-4468007/v1>.

118. Wilson-Costello D, Borawski E, Friedman H, Redline R, Fanaroff AA, Hack M. Perinatal correlates of cerebral palsy and other neurologic impairment among very low birth weight children. Pediatrics. 1998;102(2 Pt 1):315-22.

119. Winer EK, Tejani NA, Atluru V, DiGiuseppe R, Borofsky LG. Four- to seven-year evaluation in two groups of small-for-gestational age infants. Am J Obstet Gynecol. 1982;143(4):425-9. doi: 10.1016/0002-9378(82)90085-0. PubMed PMID: 7091208.

120. Withagen MIJ, Wallenburg HCS, Steegers EAP, Hop WCJ, Visser W. Morbidity and development in childhood of infants born after temporising treatment of early onset pre-eclampsia. BJOG : an international journal of obstetrics and gynaecology. 2005;112(7):910-4.

121. Zen M, Schneuer F, Alahakoon TI, Nassar N, Lee VW. Perinatal and Child Factors Mediate the Association between Preeclampsia and Offspring School Performance. The Journal of pediatrics. 2021;238(jlz, 0375410):153-60.e4. doi: <https://dx.doi.org/10.1016/j.jpeds.2021.06.069>.
